# Different diseases, different needs: Patient preferences for gene therapy in lysosomal storage disorders, a probabilistic threshold technique survey

**DOI:** 10.1186/s13023-024-03371-y

**Published:** 2024-10-03

**Authors:** Eleonore M. Corazolla, Eline C. B. Eskes, Jorien Veldwijk, Marion M. M. G. Brands, Hanka Dekker, Erica van de Mheen, Mirjam Langeveld, Carla E. M. Hollak, Barbara Sjouke

**Affiliations:** 1 Department of Endocrinology and Metabolism, Amsterdam UMC, University of Amsterdam, Meibergdreef 9, Amsterdam, The Netherlands; 2 Laboratory Genetic Metabolic Diseases, Amsterdam UMC, University of Amsterdam, Meibergdreef 9, Amsterdam, The Netherlands; 3 Inborn Errors of Metabolism, Research Institute of Amsterdam Gastroenterology Endocrinology and Metabolism, Meibergdreef 9, Amsterdam, The Netherlands; 4 Erasmus School of Health Policy and Management, Erasmus University Rotterdam, Rotterdam, The Netherlands; 5 Erasmus Choice Modelling Centre, Erasmus University Rotterdam, Rotterdam, The Netherlands; 6 Department of Pediatrics, Division of Metabolic Diseases, Emma Children’s Hospital, Amsterdam UMC, Location University of Amsterdam, Meibergdreef 9, Amsterdam, The Netherlands; 7 The Dutch Patient Association for Inherited Metabolic Diseases (VKS), Zwolle, The Netherlands; 8 Fabry Support and Information Group the Netherlands (FSIGN), Drachten, The Netherlands; 9 Department of Internal Medicine, Radboudumc, Nijmegen, The Netherlands

**Keywords:** Lysosomal storage diseases, Gaucher disease type 1, Fabry disease, Mucopolysaccharidosis type III A/B, Gene therapy, Probabilistic threshold technique, Patient preferences

## Abstract

**Background:**

Gene therapy is currently in development for several monogenetic diseases including lysosomal storage disorders. Limited evidence is available on patient preferences for gene therapy in this population. In this study, we compare gene therapy-related risk tolerance between people affected by three lysosomal storage diseases currently faced with different therapeutic options and prognoses.

**Methods:**

A survey including the probabilistic threshold technique was developed in which respondents were asked to choose between gene therapy and the current standard of care. The attributes included to establish participants’ risk tolerance were previously identified in focus groups of affected people or their representatives, namely: risk of mild side effects, severe side effects, the need for additional medication, and the likelihood of long-term effectiveness. The survey was distributed among people receiving outpatient care for type 1 Gaucher disease (good prognosis with current treatment options), Fabry disease (varying prognosis with current treatment options, XY-genotype on average more severely affected than XX), and parents representing people with severe forms of mucopolysaccharidosis type III A/B (poor prognosis, no disease-specific therapy available).

**Results:**

A total of 85 surveys were completed (15 Gaucher disease respondents, 62 Fabry disease respondents (17 self-identifying male), eight parents of ten people with mucopolysaccharidosis type III). Disease groups with higher disease severity trended towards higher risk tolerance: Gaucher disease respondents were most cautious and predominantly preferred the current standard of care as opposed to MPS III representatives who were more risk tolerant. Respondents with Fabry disease were most heterogeneous in their risk tolerance, with male participants being more risk tolerant than female participants. Long-term effectiveness was the attribute in which respondents tolerated the least risk.

**Conclusions:**

People affected by a lysosomal storage disease associated with a poorer prognosis and less effective current treatment options trended towards more risk tolerance when choosing between gene therapy and the current standard of care. This study shows the importance of involvement of patient preferences before and during the development process of new treatment modalities such as gene therapy for rare diseases, to ensure that innovative therapies align with the wishes and needs of people affected by these diseases.

**Supplementary Information:**

The online version contains supplementary material available at 10.1186/s13023-024-03371-y.

## Introduction

Gene therapy (GT) has been under development for three decades based on the hypothesis that monogenetic diseases may be curable by introducing a therapeutic gene [1, 2]. Most of the gene therapy products that have advanced to clinical trials use the in vivo or ex vivo GT approach. In this context, in vivo GT consists of an injection of an adeno(-associated) viral vector containing the therapeutic gene which inserts itself into the host cell genome [1, 3]. Ex vivo GT involves the pharmacologic mobilization and extraction of hematopoietic stem cells, followed by bone marrow eradicating chemotherapy, the introduction of the corrected allele into the extracted stem cells using retroviral vectors, and infusion of the successfully engineered autologous cells [1]. The burden of GT varies greatly depending on the approach.

Both in vivo and ex vivo approaches are being investigated in clinical trials in monogenic lysosomal storage disorders (LSDs) [3]. To date this has resulted in the approval of one GT product for an LSD by the European Medicines Agency (EMA): atidarsagene autostemcel (Libmeldy®; Orchard Therapeutics BV) for specific patient groups with Metachromatic Leukodystrophy (OMIM 250100) [4]. Currently available therapies for LSDs are enzyme replacement therapy (ERT), substrate reduction therapy (SRT), and chaperone therapy [5] (see Suppl Table 1). Ideally, successful GT would solve shortcomings of these current therapeutic options such as the high administration frequency and lack of penetration of the blood–brain barrier. However, GT involves new risks, some of which may yet be unknown, and the long-term effect on disease progression is unclear.

With the current therapeutic strategies in play, two of the factors that simultaneously influence the impact of LSDs are disease severity and the effectiveness of available therapies [6, 7]. This combination encompasses a spectrum within and between each separate LSD. One end of this spectrum is illustrated by Mucopolysaccharidosis III A/B (MPS III; OMIM 252900), a neurometabolic disorder which strongly impacts cognitive development from an early age, reduces quality of life and life expectancy, and for which there is currently no approved therapy [8–10]. At the opposite end of the spectrum is Gaucher disease type 1 (GD; OMIM 230800), a condition which mainly affects the liver, spleen, and bone marrow [11]. The impact of GD is drastically reduced with adequate treatment with ERT or SRT, resulting in near normal life-expectancy [12, 13]. Other LSDs are characterized by a broader range of intra-disease variability, such as Fabry disease (FD; OMIM 301500), which mainly affects the heart, kidneys, and nervous system [14, 15]. In FD, disease severity and therapeutic effectiveness of ERT and chaperone therapy are very variable [15, 16]. Therefore, both the impact of treatment and disease prognosis vary strongly [16, 17]. On average, people with XY-genotype are more severely affected by FD due to its X-linked inheritance, and have a poorer prognosis [14]. In addition, FD can present as the classical form with early-onset and more rapidly progressive disease, or as the generally milder non-classical form [14]. To date, one clinical trial of GT in men with classical FD has been published [18].

Since the disease manifestations, therapeutic options, and GT administration routes all impact recipients very differently, the considerations of people with LSDs regarding GT are not obvious. To date, this has not been a topic of in-depth research, with the current literature on patient preferences for GT in LSDs limited to focus groups of people with GD, FD and MPS III by our research group, which established the attributes for this study, and a recent survey by the International Gaucher Association [19, 20].

This lack of research prior to or at least concurrently with the development of GT for LSDs is especially surprising given the fact that patient preferences differ from those of healthcare professionals, and given the wish of potential therapy recipients and other stakeholders to include patient preferences in regulatory decisions [21–23].

Therefore, in this study we investigated the preferences of people affected by LSDs regarding the risks and benefits associated with GT compared to their current situation. We hypothesized that risk tolerance regarding GT differs between LSDs that have varying impacts on people’s lives, as well as general beliefs about medicine and personal experience [24]. To investigate this, we developed and conducted a survey based on relevant attributes identified in previous research, using the probabilistic threshold technique method to elicit risk tolerance [19, 25, 26].

## Methods

### Expert panel, participant selection and recruitment

**Fig. 1:**
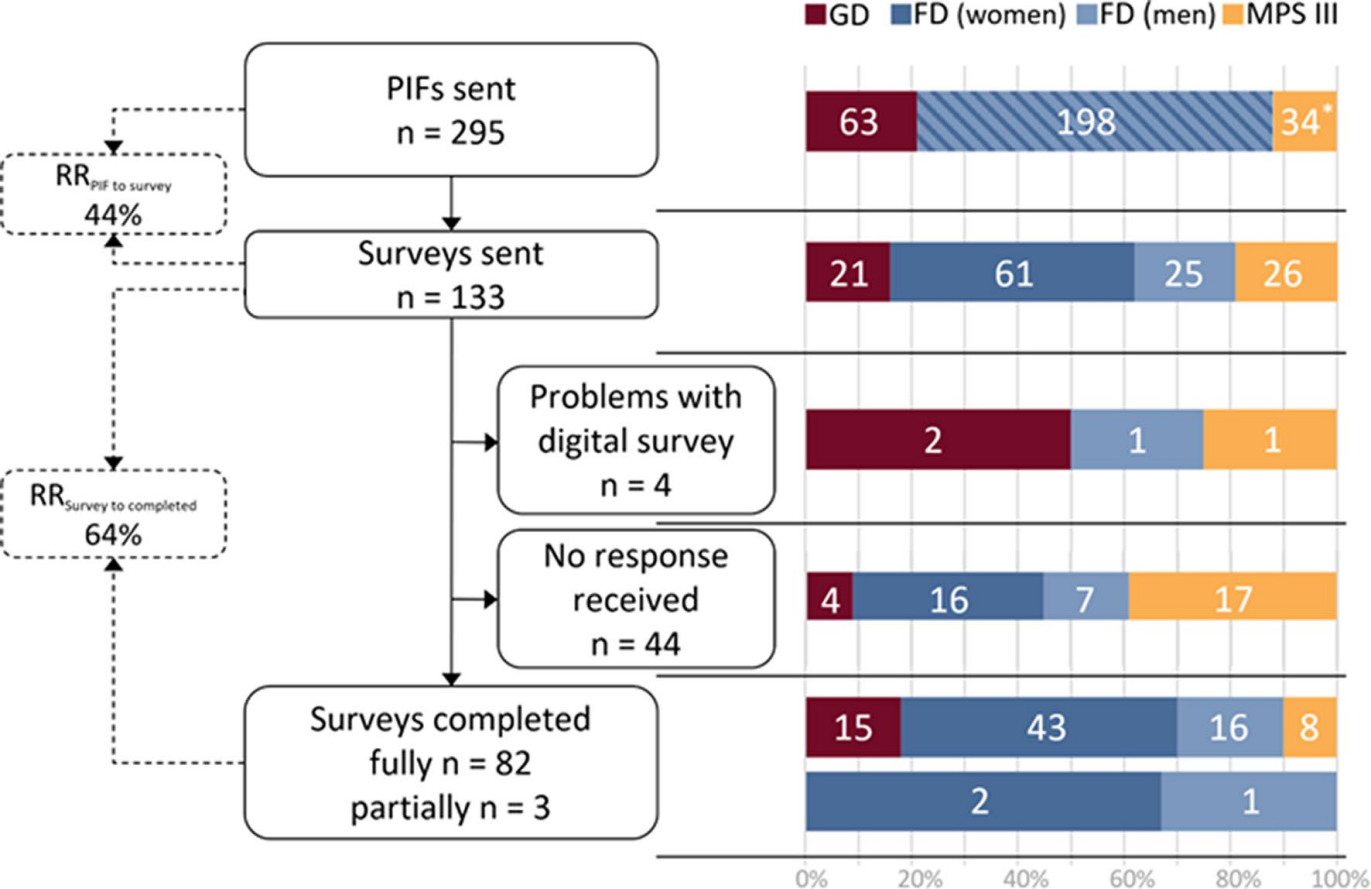
Recruitment of participants. Numbers in the stacked bar graphs represent the absolute number of participants per group, the x-axis represents the percentage of all participants over all groups for the respective step of the recruitment process. * This is the number of people with MPS III whose parents were contacted. In all following rows the MPS III number represents the number of surveys (and thus parents). One MPS III patient died after their parents had given consent for the study but before they received the link to the survey, therefore these parents were not sent a survey link. Abbreviations: *FD* Fabry disease, *GD* Gaucher disease type 1, *MPS III* Mucopolysaccharidosis type III A/B, *PIF* participant information form, *RR* Response rate

An expert panel was formed consisting of representatives of patient associations (HD, EM) and clinical experts in the field of metabolic diseases in adults (ML, CH, BS) and children (MB). The expert panel was involved throughout the study as described below. Three subgroups of people diagnosed with LSDs were selected by the expert panel to represent a spectrum of clinical severity, prognosis and available therapeutic options: adults with Gaucher disease type 1, adults with Fabry disease (male and female, classical and non-classical), and people severely affected by MPS III types A and B. Participants were recruited via the outpatient clinics of the Amsterdam UMC location AMC, the national referral center for all three LSDs, which provides outpatient care to almost all people diagnosed with GD, FD, and MPS III in the Netherlands. For people with MPS III, representatives (one or two parents) were recruited since most people under care were either children and/or cognitively impaired. All people from the selected groups over 18 years of age, or—in the case of people with MPS III—their parents were invited to participate in the survey if they met the inclusion criteria of receiving the diagnosis at least one year previously, sufficient proficiency in Dutch and legal competence (Fig. 1).

### Measuring preferences: probabilistic threshold technique

A probabilistic threshold technique (PTT) was used to elicit the maximum risk that participants (or their representatives) find acceptable for GT compared to the current standard of care (i.e. ERT, SRT, or supportive care) [27]. This is what we refer to as participants’ risk tolerance. This method has been identified as robust to quantify treatment preferences in a healthcare setting [28]. In a PTT, respondents are asked to choose between two treatment profiles, of which one has a higher benefit than the other, but also imposes risks. Subsequently, the level of risk in the profile providing the beneficial treatment is varied until respondents switch their preferred treatment to the alternative treatment profile [26]. This process is repeated varying the risk levels of different aspects of treatments (e.g. side effects), which are called attributes (Box 1).

**Box 1:** Definitions

• **Attributes** are the characteristics used to describe the good of service under study in each choice task, in other words the explicitly named factors that play a role in the choice offered in the PTT [60]—in this case the choice between GT and the current standard of care. To distill which factors respondents consider when making the choice offered in this study, focus groups were previously conducted in which this choice was discussed [19]
• The **attribute level** is the risk of a certain attribute occurring, i.e. if there is a 5% chance of developing severe side effects, “severe side effects” is the attribute and 5% is the attribute level
• The **base case** in this study is the attribute level based on relevant literature; it is an approximation of the true, real-world risk. The other attribute levels used in the survey are fictional
• **Risk tolerance** is defined in this study as the maximum risk that respondents find acceptable for GT compared to current standard of care, expressed as a percentage (i.e. if a respondent states that they choose GT if there is a 5% chance of developing severe side effects, but will choose the current standard of care if there is a 10% chance of developing severe side effects, then their risk tolerance is 5–10% for the attribute “severe side effects”)
• Each **task** within the survey determines the risk tolerance of the participant regarding one attribute. Each task in this study consists of three choices, each with a different attribute level for the attribute being tested in that particular task. All other levels remain identical

### Survey design

**Fig. 2:**
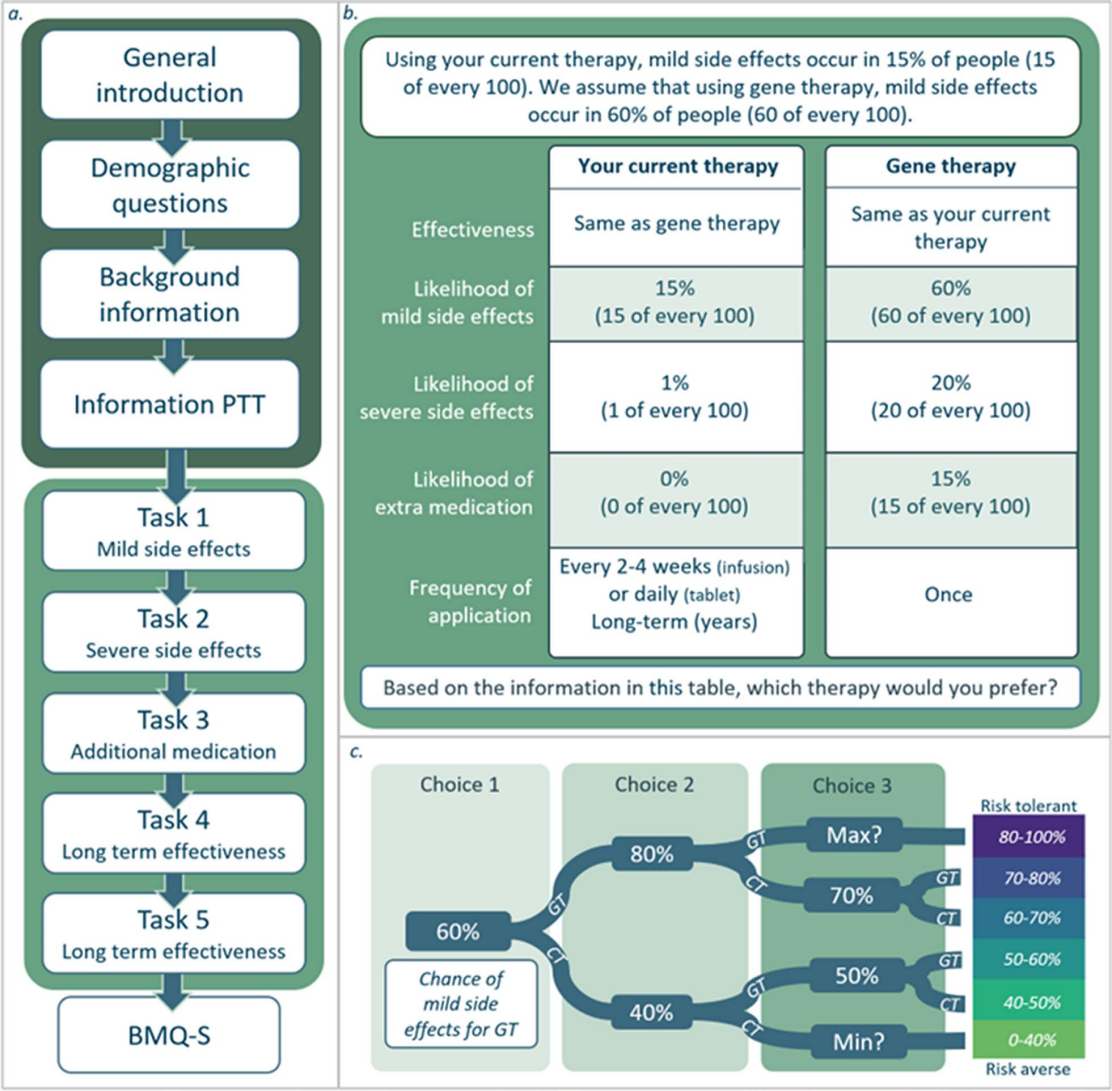
Structure of the survey. **a** *High level structure*. **b** *Example of the presentation of one level of a task.* In every task a table displayed the levels per attribute. In this case the levels are from task 1 (identical in all disease groups), and from a respondent who received therapy (hence the expression “your current therapy”). **c** *Example of flow of levels through a task.* Participants were all given the same initial choice and the levels in subsequent choices varied depending on their answers as illustrated. After three choices, participants fell into one of six risk tolerance categories for this particular attribute. If participants ended up in the highest or lowest risk category after the second choice, they were asked to state respectively the maximum or minimum risk they would tolerate for this attribute. These risk categories are represented in the color scale, ranging from risk aversive (light green) to risk tolerant (dark blue). The numbers shown in this figure are also from task 1 (identical in all disease groups). Abbreviations: *BMQ-S* Beliefs in Medicine Questionnaire specific subscale, *CT* current therapy, *GT *gene therapy, *Max* maximum risk tolerance, *Min* minimum risk tolerance, *PTT* probabilistic threshold technique

The survey was designed in Dutch and consisted of three parts (Fig. 2a; Suppl Material 1). In the first part, respondents were asked to complete demographic questions on age (in decades), sex, and current treatment status. Next, background information on GT was provided based on an extensive review of the literature. Although information was provided about in vivo and the ex vivo GT methods, respondents were instructed to consider both methods as “gene therapy” during the survey.

Respondents were asked to imagine that GT would become available to them. Depending on what participants reported as their current therapy (e.g. currently using ERT or SRT, discontinued ERT or treatment naïve), FD and GD participants were asked to compare GT to ERT or SRT (Table 1). Nobody with FD using chaperone therapy consented to be included in the study, therefore this option was not included. All parents of people affected by MPS III were asked to compare GT to the current situation in which there were no disease specific treatment options (Table 1).

**Table 1:** Treatment options per disease group and comparator

Group	Current therapy	Comparator to GT			Based on			
		ERT	SRT	No therapy	Current situation	Experience prior to stopping ERT	Experience prior to clinical trial	Theoretical information
GD	ERT: imiglucerase	●			●			
	ERT: velaglucerase	●			●			
	SRT: eliglustat		●		●			
FD	ERT: agalsidase alfa	●			●			
	ERT: agalsidase beta	●			●			
	ERT discontinued	●				●		
	ERT discontinued for participation in clinical trial	●					●	
	Therapy naive	●						
MPS III	Therapy naive			●		●		●
	Clinical trial			●				●

*Abbreviations: ERT* enzyme replacement therapy, *FD* Fabry disease, *GD* Gaucher disease type 1, *GT* Gene therapy, *MPS III* Mucopolysaccharidosis type III A/B, *SRT* substrate reduction therapy

**Fig. 3:**
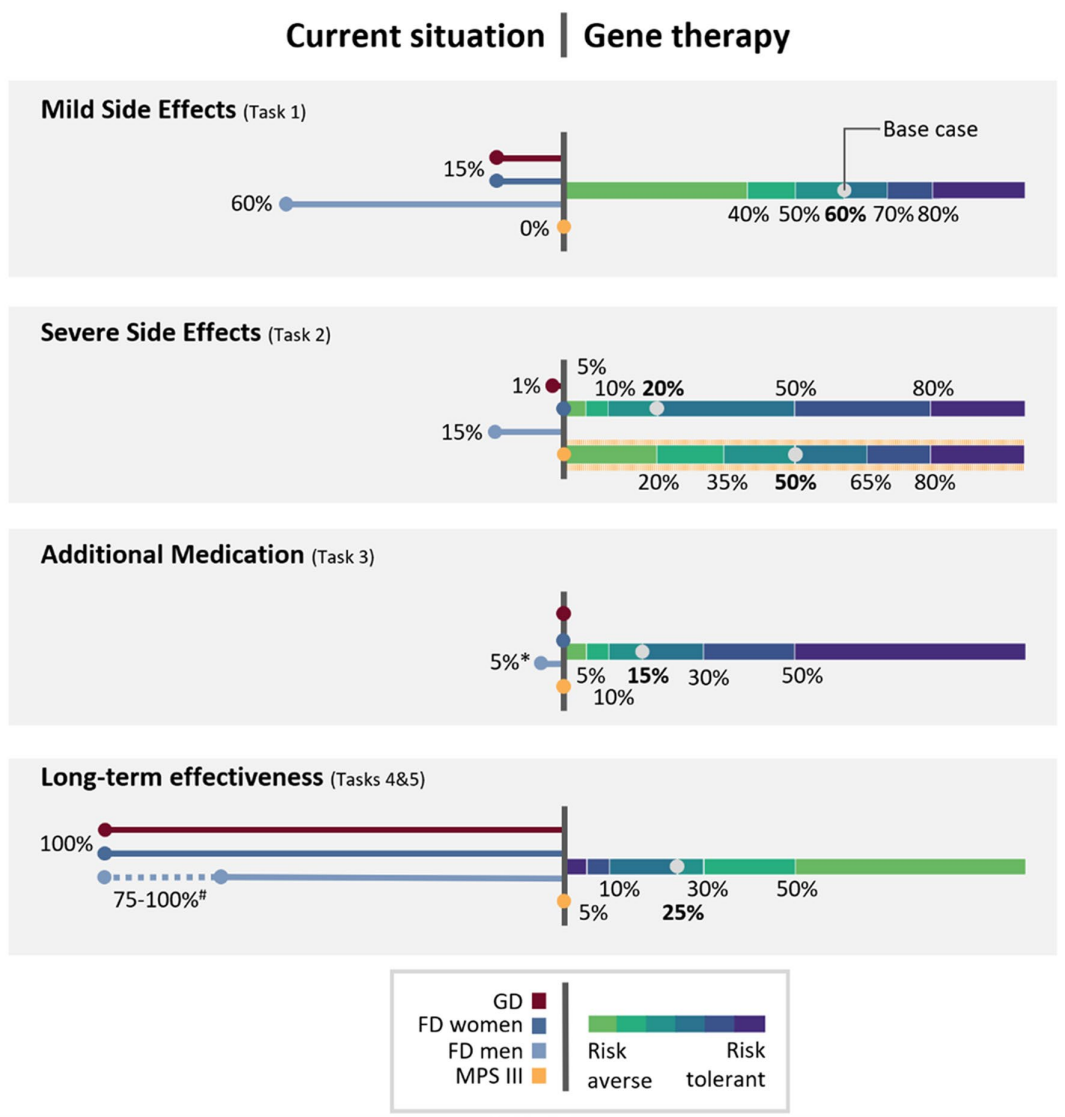
Base case and threshold levels used in the survey. In this figure the levels of the attributes used throughout the survey are stated. In task two, the levels used for MPS III gene therapy differed from those of the other three groups, since the data from intracerebral gene therapy trials, which have a higher risk of severe side effects, were taken into account when determining the base case level for MPS III but not GD and FD. The levels used for MPS III are depicted separately, indicated by the yellow background. In tasks four and five the attribute “Likelihood of long-term effectiveness” was tested—in task four under the assumption of equal effectiveness between the current standard of care and GT, and in task five GT was assumed to be more effective, in both tasks using the same attribute levels. Level ranges of tasks four and five are reversed because in these cases, conversely to the other tasks, a lower likelihood equals a higher risk. See Box 1 for definitions of the terms used throughout this figure, and Fig. 2 for more details on the structure of the questionnaire. The references on which the base cases and current therapy numbers are based are listed in Suppl Table 2. For the gene therapy side, the color scale depicts the categories of risk tolerance towards gene therapy compared to current standard of care ranging from risk aversive (light green) to risk tolerant (dark blue) as explained in Fig. 2c. * Based on data from the Amsterdam UMC patient registry, data not shown. # In the survey for male FD participants, effectiveness was presented as a range since ERT has shown variable effectiveness in this group. Abbreviations: *ERT* enzyme replacement therapy, *FD* Fabry disease, *GD* Gaucher disease type 1, *GT* gene therapy, *MPS III* Mucopolysaccharidosis type III A/B

In the second part, respondents were presented with five PTT tasks focusing on four attributes (Fig. 2b). Attributes included were selected in a stepwise procedure of assessing the literature, performing a qualitative study in a subset of the participants of this survey [19], and internal meetings with both clinical (e.g. the expert panel) and a methodological expert in the field (JV) (Fig. 3). Four attributes were included: [1] the risk of mild side effects (SE), [2] the risk of severe SE, [3] the likelihood of needing to take additional medication, [4] the likelihood of long-term effectiveness. Attribute [4] was tested with two tasks (see description below). In the survey the definitions of mild and severe side effects were explained (see Suppl Material 1). A side effect was considered severe if it led to hospitalization.

The likelihood of one attribute for GT was varied per task starting at a baseline level and either increased or decreased, depending on the participant’s initial response (Fig. 2c). The levels of the other attributes for GT were fixed, as well as all attributes of the current standard of care option. Respondents were instructed to assume both treatments were equally effective and to assume that GT would be administrated once. For MPS III, comparison with a current therapy was not possible. Therefore, GT was assumed to reduce symptoms and slow disease progression.

In the final two tasks (focusing on long term effectiveness) an additional attribute was introduced, namely the likelihood that GT proves safe and effective in the long term. In the fourth task GD and FD respondents were asked to assume equal effectiveness of their current standard of care option and GT for up to two years. MPS III parents were asked to assume that GT would reduce symptoms and slow disease progression. In the fifth task, the same levels were presented, but GD and FD respondents were instructed to assume better effectiveness of GT than the current standard of care. MPS III respondents were asked to assume GT to prevent new symptoms and fully stabilize the disease at the current stage.

After each task, participants were asked whether they had personal experience with the attribute investigated in that task (respectively: mild SE, severe SE, additional medication, or participation in a clinical trial).

In the third part of the survey, respondents completed the specific part of the Beliefs in Medicine Questionnaire (BMQ-S) [29]. The Dutch version of the BMQ-S consists of two subscales: the necessity subscale (a sum of five items with 5-point Likert scale responses; low defined as ≤ 16 points) and the concern subscale (a sum of six items with 5-point Likert scale responses; low defined as ≤ 13 points) [30]. Based on these two subscales, four attitudes of respondents towards their medication were distinguished: acceptant (high on the necessity scale, low on the concern scale), ambivalent (high on the necessity scale and on the concern scale), indifferent (low on the necessity scale and on the concern scale) and skeptical (low on the necessity scale, high on the concern scale) [24].

All members of the expert panel reviewed and pre-tested the survey to refine wording, layout, and programming errors. For FD, separate surveys were developed for men and women because of the large discrepancy in estimated probability of side effects and additional medication, mainly due to risk of anti-drug antibodies in men with classical FD using ERT.

### *Attributes, attribute levels and base case levels (*Fig. 3*)*

Attribute levels for current standards of care were determined based on a literature review of current therapies and remained constant throughout the survey. In MPS III the lack of current therapeutic options was represented by a 0% probability of all attributes. For GT, one attribute per task was varied while all other attribute levels remained fixed at the base case level for that task. The base case was defined as the estimated risk of a particular attribute occurring based upon published GT trials in other inborn errors of metabolism or inherited diseases (cut-off date June 2021; Fig. 3; Suppl Table 2). The base case level was used in the first choice of a task, next fictional levels were presented depending on the participants risk tolerance towards the base case, to estimate the participants´ overall risk tolerance. Level ranges for MPS III differed from the other disease groups in task two (severe side effects), because trials using intracerebral GT application, which involve more severe side effects, were also considered in MPS III but not in the other disease groups (Fig. 3; Suppl Table 2).

To estimate the base case level of the attribute “likelihood of long-term effectiveness”, the likelihood of GT becoming approved as a registered drug for GT served as a surrogate (Fig. 3; Suppl Table 2). This likelihood was approximated based upon the stage of clinical trials on GT in the respective diseases at the time of the survey design (June 2021; Fig. 3; Suppl Table 2), and the likelihood of success of metabolic/endocrinology clinical trials in those stages as described by Wong and colleagues [31]. For GD, no trials were published at the time of cutoff and one phase 1/2 trial of ex vivo GT was ongoing [32, 33, 88, 89]. For FD, one phase 1 trial had been published [18] and one ex vivo [38] and three in vivo  [32, 33, 36, 37] GT trials in phase 1/2 were ongoing. For MPS III, one intracerebral GT trial had been published and was moving to phase 2/3 [39], one ex vivo had been published [35], and three in vivo intravenous [40–43] GT studies were ongoing in phase 1/2 and one intracerebral GT study was ongoing in phase 2/3 [44, 45]. Based on this, the base case was set at 25%, which is the probability of phase 2 trials for metabolic/endocrinology drugs ultimately result in regulatory approval [31] (Suppl Table 2).

### Data collection and analysis

After reading the participant (representatives’) information and signing informed consent, participants were sent a personal hyperlink to the electronic survey via email. In the case of MPS III, two personal links were sent and parents were given the option to fill in the survey separately or together. Technical support was offered to people who required assistance opening the questionnaire. A reminder was sent to participants who had not yet completed the survey after two to four weeks. Data of all participants who had completed at least one task were included in the data analysis. Data from a task were excluded if there was a discrepancy in the internal control of the answers (n = 5). Correlation coefficients between participant characteristics and choices in the PTT (analyzed as a categorical variable as depicted in results section) were calculated using the Spearman rho method. Correlation coefficients are only stated in the results section if subgroups were sufficiently large for formal analysis. Age groups were compared using the mean of midpoints of the decade age brackets respondents were offered. Data analyses and visualization were performed using R Studio (version 4.0.3).

### Ethical approval and privacy

The Medical Ethics Committee of the Amsterdam UMC, location AMC, reviewed the study protocol and waived the need for ethical approval (W20_380 # 20.425). Compliance with data protection regulations under the General Data Protection Regulation was ensured, as assessed by a data protection impact assessment under the supervision of the privacy officer of the Amsterdam UMC. All participants gave written consent to participate in the study after being informed of the aim and method of the study, and background information on GT. The study was performed in accordance with the Declaration of Helsinki.

## Results

### Participants

A total of 295 people (or their representatives) were approached, of which 121 consented to participate in the study (Fig. 1). Surveys were sent to 133 people (107 affected people and 26 parents from 11 families), of which we included a total of 82 completed (15 GD, 43 FD women, 16 FD men, and eight parents of ten people affected by MPS III), and three partially completed surveys (two FD women, one FD man) (Fig. 1, Table 1). The overall response rate (RR) to consent forms was 44% (Fig. 1). The overall RR to surveys was 64% (Fig. 1). Four people who consented could not participate due to problems with the digital questionnaire: three did not have an email address, one participant could not open the questionnaire digitally and was not available for troubleshooting. Ninety-six percent of participants who started the survey also completed it. The age ranges varied between GD and FD. GD respondents’ median age was 65 years (range 50–79 years), while the FD respondents’ median age was 55 years for FD women and 45 years for FD men (range for all FD participants 18–69 years) (Table 2). Most participants with GD or FD were currently treated with ERT, and there were more classical than non-classical FD participants included of both sexes (Table 2). In the MPS III group, a total of eight surveys was completed by nine parents of ten children with MPS III, including two sets of sibling pairs (Table 2).

**Table 2:** Demography of participants

	GD	FD		MPS III^#^
		Women	Men	
*Demographics*				
Number	15	45	17	10*
Female sex (n [%])	8 [53%]	45 [73%]		5 [50%]
*Age*				
< 18 years (n [%])	–	–	–	4 [40%]
> 18 years (n [%])	–	–	–	6 [60%]
18–29 years (n [%])	0 [0%]	8 [18%]	5 [29%]	–
30–39 years (n [%])	0 [0%]	5 [11%]	2 [12%]	–
40–49 years (n [%])	0 [0%]	7 [16%]	2 [12%]	–
50–59 years (n [%])	7 [47%]	14 [31%]	3 [18%]	–
60–69 years (n [%])	5 [33%]	11 [24%]	5 [29%]	–
70–79 years (n [%])	3 [20%]	0 [0%]	0 [0%]	–
*Disease subtype*				
Gaucher disease type 1 (n [%])	15 [100%]	–	–	–
Classical Fabry disease (n [%])	–	36 [80%]	10 [59%]	–
Non–classical Fabry disease (n [%])	–	9 [20%]	7 [41%]	–
MPS III A (n [%])	–	–	–	6 [60%]
MPS III B (n [%])	–	–	–	4 [40%]
*Current treatment*				
ERT (n [%])	13 [87%]	21 [47%]	11 [65%]	–
Imiglucerase	12	–	–	–
Velaglucerase	1	–	–	–
Agalsidase alfa	–	1	0	–
Agalsidase beta	–	20	11	–
SRT (n [%])	2 [13%]	0 [0%]	0 [0%]	–
Eliglustat (n [%])	2	–	–	–
Investigational treatment (clinical trial) (n [%])	0 [0%]	1 [2%]	1 [6%]	3 [30%]
Discontinued standard of care [%])	0 [0%]	4 [9%]	1 [6%]	
Treatment naive (n [%])	0 [0%]	19 [42%]	4 [23%]	7 [70%]
Joined in focus groups (n [%]) [18]	8 [53%]	10 [22%]	3 [18%]	4 [40%]

^#^Characteristics of children with MPS III are depicted, surveys were filled in by their parents or legal representatives. *Eight surveys were completed by nine parents of ten MPS III children (two respondents had two children each with MPS III, in one case the survey was completed by two parents together). *Abbreviations: FD* Fabry disease, *GD* Gaucher disease type 1, *MPS III* Mucopolysaccharidosis type III A/B, *ERT* enzyme replacement therapy, *SRT* substrate reduction therapy

### *Mild side effects (*Fig. 4a*; Suppl Fig. *1)

**Fig. 4:**
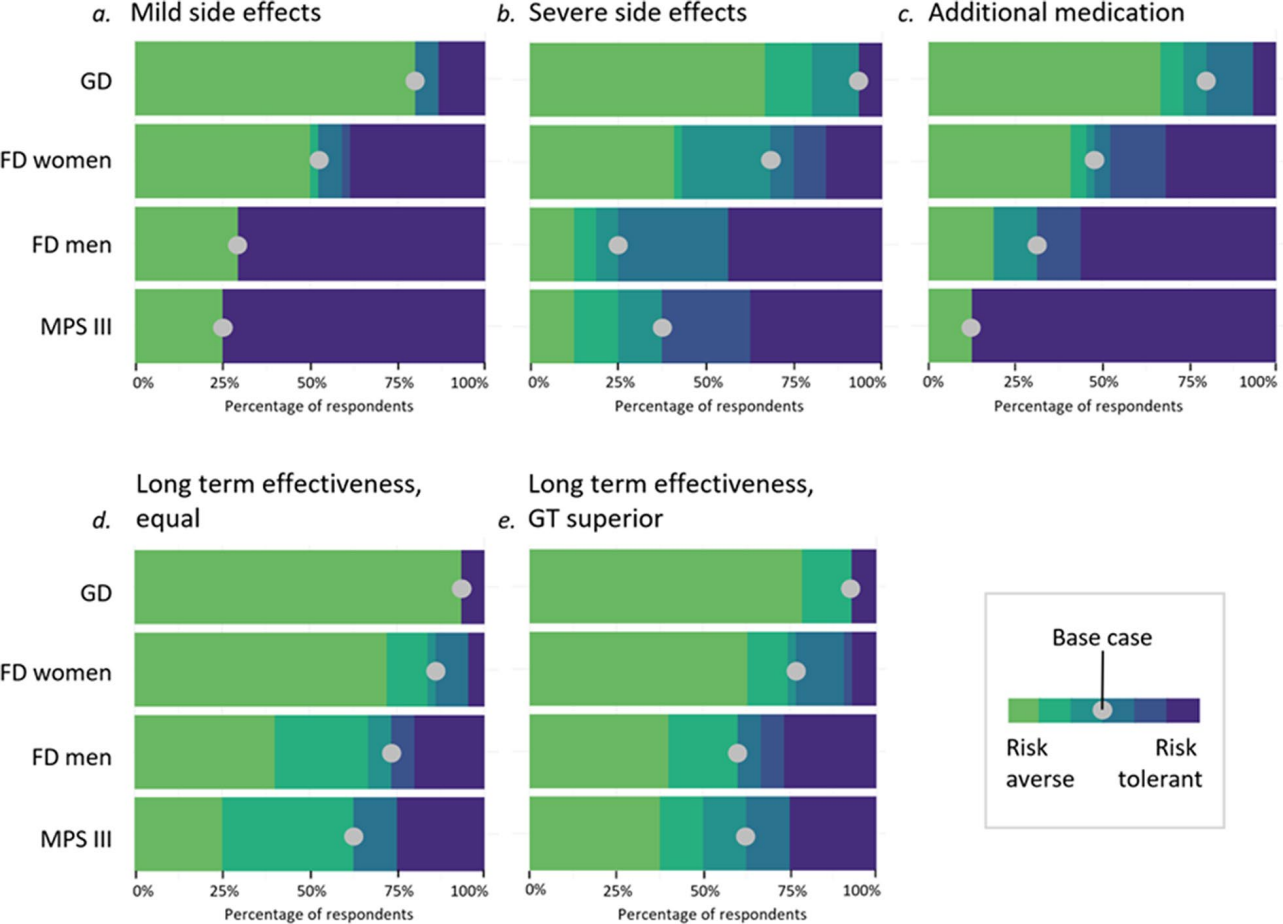
Distribution of risk tolerance categories per attribute and disease group. The base case is depicted to indicate the percentage of respondents whose risk tolerance was higher or lower than the estimated real-world risk (represented by the base case; see Fig. 2 for attribute levels of the base case). The color scale depicts the categories of risk tolerance towards gene therapy compared to current standard of care ranging from risk aversive (light green) to risk tolerant (dark blue) as explained in Fig. 2c. *Abbreviations: FD* Fabry disease, *GD* Gaucher disease type 1, *GT* gene therapy, *MPS III* Mucopolysaccharidosis type III A/B

In GD respondents a minority of 13% (n = 15) always chose GT. Eighty percent of participants always chose the current standard of care. Fourty-two percent of respondents stated they accept no risk of mild side effects at all (0% as minimum risk threshold). In female FD participants (n = 44) there was an almost equal bimodal distribution with 39% and 50% of respondents always choosing either GT or current standard of care, respectively. In male FD respondents (n = 17) a bimodal distribution was also present, however less equally distributed than for FD women. The majority (71%) of FD men always chose GT, and 58% of the respondents were prepared to accept a 100% risk of mild SE. In MPS III (n = 8), this bimodal distribution was shifted even further towards GT: 75% of respondents always chose GT, and 83% of respondents accepted 100% risk of mild SE. The remaining 25% of MPS III participants always chose the current standard of care (i.e., no therapy).

### *Severe side effects (*Fig. 4b*; Suppl Fig. *1)

In GD participants (n = 14) the results for severe SEs were generally similar to those of mild SEs: while 33% chose GT at least once thus stating a risk threshold above 5%, 67% of respondents again consistently chose the current standard of care. In female FD respondents (n = 44), only 16% always chose GT, and overall the risk tolerance was distributed more evenly compared to mild SE for this group. In the male FD group (n = 16) fewer respondents chose GT at every level (44% of respondents). Male FD respondents were more risk averse for severe SEs than for mild SEs but remained more risk tolerant than GD and female FD respondents for this attribute. Of MPS III respondents (n = 8), 38% always chose GT. The distribution of risk categories was wider for severe SEs than for mild SEs: while all MPS III respondents fell into the highest or lowest risk category for mild SE this was only the case for 51% in the severe SE (Fig. 4a, b). Overall, in male FD respondents the largest fraction tolerated risk above the base case level (thus the estimated real-world risk): 7%, 32% and 63% of GD, female FD and MPS III respondents respectively are in the categories in which risk tolerance is higher than the base case (thus the estimated real-world risk) whereas in FD men this is 75%. However, in MPS III the base case risk was higher than in the other disease groups for this attribute (Fig. 3).

### *Additional medication (*Fig. 4c*; Suppl Fig. *1)

Among GD participants (n = 15) only 7% chose GT at every level. The majority (67% of respondents) chose the current standard of care at every level with low risk tolerance (0% or 1%). However, for this attribute 27% of respondents stated a moderate risk tolerance (meaning neither of the most extreme risk tolerance categories), which makes this the most evenly distributed attribute for GD participants. In female FD participants (n = 44) the distribution was also more even than other attributes with 32% always choosing GT and 21% stating a moderate risk threshold. In the male FD group (n = 16) the responses were similar to the mild SE attribute, with 56% of respondents choosing GT. In MPS III (n = 8) the vast majority chose GT (88% of respondents). In MPS III, in contrast to other disease groups, no moderate risk group was present.

### *Uncertainty of long-term effectiveness (*Fig. 4d and e*; Suppl Fig. *1)

In GD respondents (n = 15) the distribution was similar to previous attributes with only 7% of respondents choosing GT and 93% always choosing current standard of care if the effectiveness of GT and current standard of care were equal. In the context of more effective GT, the same 7% always chose GT, however a subset of the group that chose the current standard of care under the assumption of equal effectiveness was slightly more risk tolerant (14% of total respondents). In the female FD group (n = 43) only 5% chose GT if assumed to be equally effective, and more respondents chose the current standard of care than in previous attributes (86% of respondents). Under the assumption of higher effectiveness of GT, 16% chose GT if the likelihood of long-term effectiveness was 5–25%. This made them more risk-tolerant than GD participants in this attribute. In male FD respondents (n = 15), similarly to female FD respondents, fewer participants than in other attributes chose GT in the context of equally effective GT (20%), and more participants than in previous attributes (73% of respondents) chose the current standard of care. However, as in previous attributes, this group seemed markedly more risk-tolerant than female FD participants. In the context of more effective GT than the current standard of care, 27% of respondents chose GT even with only a 5% chance of long-term effectiveness, making them highly risk-tolerant in this context. In MPS III (n = 8), responses were evenly distributed in the context of mildly effective GT, with the majority (63% of respondents) opting for a minimum of 50% chance of long-term effectiveness. In the context of more effective GT, overall risk tolerance increased slightly with 36% of respondents accepting a 25–50% chance of long-term effectiveness and 25% of respondents accepting even a 0% chance.

### *Intra-individual differences in risk threshold per attribute (*Fig. 5*)*

**Fig. 5:**
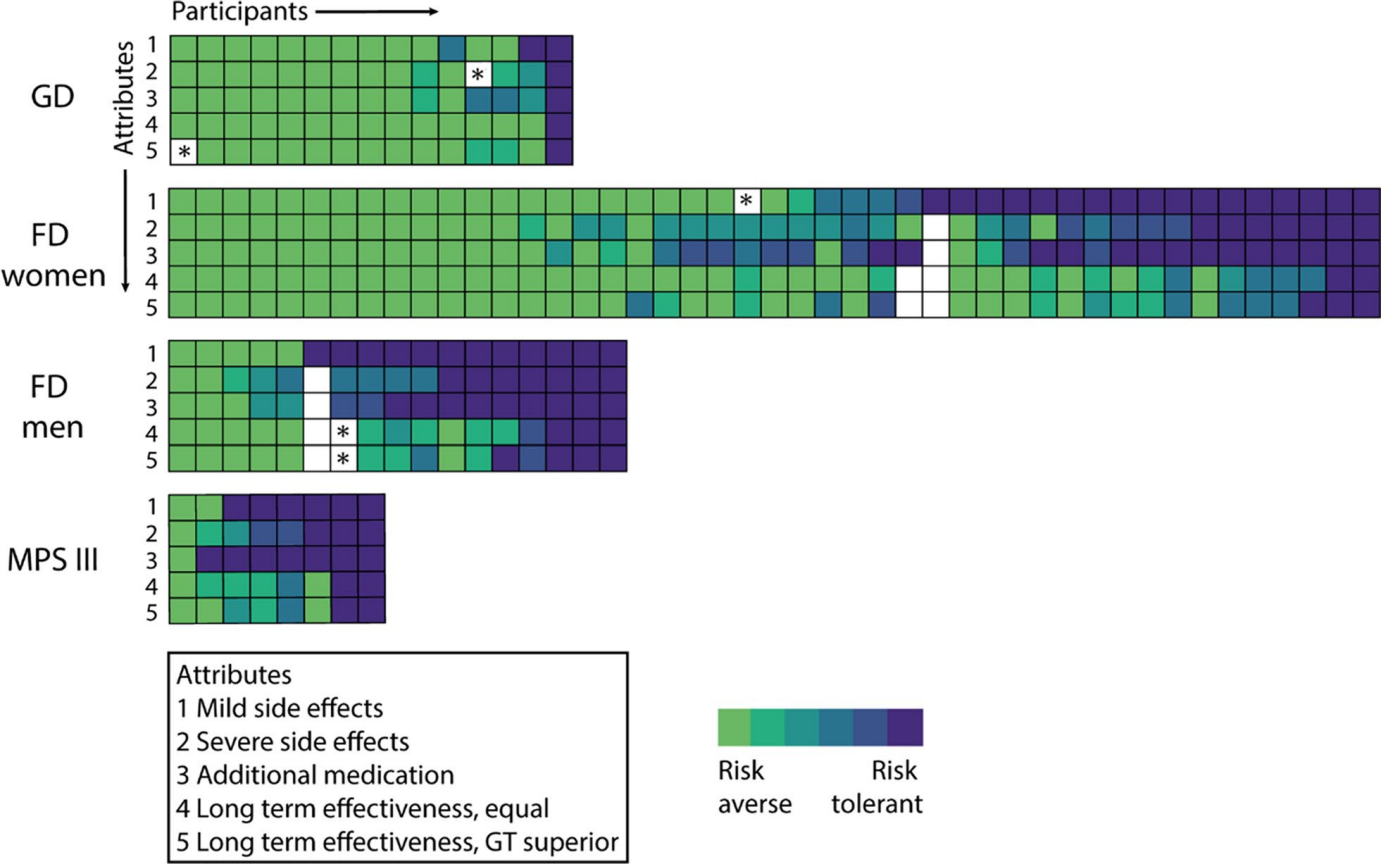
Heatmaps of risk threshold per participant. Each column represents the risk tolerance per task for one respondent. This allows for comparisons of the answers within and between participants. The color scale depicts the categories of risk tolerance towards gene therapy compared to current standard of care ranging from risk aversive (light green) to risk tolerant (dark blue) as explained in Fig. 2c. Fields marked with (*) are tasks excluded due to discrepant data in the internal control of that task. *Abbreviations: GD* Gaucher disease type 1, *GT* gene therapy, *FD* Fabry disease, *MPS III* Mucopolysaccharidosis type III A/B

Most participants consistently chose GT or the current standard of care for every attribute throughout the survey. A subset of respondents (n = 40; 47% of total) stated a lower risk tolerance regarding the uncertainty of effectiveness than for the other three attributes (Fig. 5).

### BMQ-S and risk threshold (Suppl Fig. 2)

The distribution of the four BMQ-S groups differed between the four respondent groups. Most GD participants were classified as *acceptant* and none were classed as *indifferent*, while the male FD participants were mostly *acceptant* or *ambivalent* and the female FD group was evenly distributed over all four categories. Interestingly, all MPS III respondents were classed as *indifferent*. In the male FD group, all respondents who had low risk tolerance consistently for all attributes were classed as *acceptant* (n = 3), while in the female FD and GD group there was no such trend between BMQ-S classification and responses in the tasks (Suppl. Figure 3).

### Current therapy and risk threshold (Suppl Fig. 3)

In the male FD group, all respondents who chose the current standard of care consistently for all attributes currently used ERT (n = 5). Other subgroup analyses (e.g., age, personal experience with attributes) did not show any correlation with responses in the tasks, although in most cases the groups were too small for formal analysis.

## Discussion

This study is the first systematic and quantitative investigation of preferences of people affected by LSDs when comparing GT to the current standard of care. It reveals heterogeneity in risk tolerance both between and within groups of people affected by LSDs with different impacts (i.e. prognosis and current treatment options). In this study, risk tolerance was higher in groups with higher disease severity and impact, albeit without statistical significance potentially due to small group size (Fig. 4). However, within each group of participants with the same disease, the choice between GT and the current standard of care was distributed bimodally: a subset of participants always chose GT, and another subset refused it under all or most of the tested circumstances (Fig. 4). The ratio of participants with a low or high risk tolerance differed between groups, with the highest proportion of participants choosing GT in the most severely affected disease group with the worst current prognosis (MPS III), the lowest proportion of participants choosing GT in the least severely affected disease group (GD), and a more uneven distribution in the groups with more clinical heterogeneity (FD men and women) (Fig. 4). Thus, the higher the current unmet need, the higher the proportion of participants willing to take risks to undergo GT.

Preference heterogeneity, most often bimodally distributed, has previously been described in threshold technique surveys in other congenital diseases with larger sample sizes, for example in two large studies in Belgian and US cohorts of people diagnosed with haemophilia A and B [46, 47]. This heterogeneity was also clearly stated in focus groups, both those conducted by our group as well as those in other populations such as (caregivers of) people affected by Duchenne muscular dystrophy [19, 48]. In each of these studies there was a bimodal distribution of subgroups with a very high and very low risk tolerance for GT. In line with previous studies, this study demonstrates a different ratio of participants choosing for either therapy depending on the underlying disease impact. It is important to note that this bimodal distribution was not a consequence of an over-simplified decision-making process by (caregivers of) participants. On the contrary, people affected by these diseases and their caregivers have demonstrated to be capable of nuanced and complex weighing of potential benefits and risks regarding GT [19, 48].

Perspectives of potential therapy recipients are crucial to successful implementation of new therapies, especially in innovative products and products for rare diseases [49–51]. The field of GT is evolving rapidly, and new methods of administration and gene alteration are quickly being developed [52–56]. Despite the considerable effort invested into the development of GT for LSDs, to the best of our knowledge no study has previously investigated preferences of people living with LSDs concerning GT. Therefore, this study quantifies affected people’s preferences using a design ensuring that results are translatable into real-world choices: preferences were elucidated in a disease-specific manner using attributes derived from participants’ considerations [19], and risk levels based on extensive literature review (Suppl Table 2). This study also takes into account the importance of adequate participant education prior to surveying preferences, as the level of participants’ knowledge impacts survey results and knowledge gaps regarding GT were recently elucidated by a survey among people with GD and their caregivers by the International Gaucher Alliance [20, 46].

This study faces a few limitations: reduction of complexity of the real-life situation leading to missing nuance, participation bias, low sample size, and differences in composition, perspectives and previous experiences that may have been influenced by the length of being diagnosed or treated of the participants representing disease groups. When designing a survey to research such a nuanced topic as this, concessions must be made to balance this nuance with the complexity and length of a survey that can realistically be completed by participants. In the present study, one such concession was the reduction of in vivo and ex vivo GT into a single modality (“gene therapy”). While this does not capture the nuances of the difference in the impact of each GT strategy, the choice of a given GT modality versus standard of care closely resembles the real-life choice people might face in the future. Another concession regarding the complexity of GT is the assumption of a single treatment in GT.

Participation bias might play a role in this survey, since the topic of the survey may have attracted mostly people with strong opinions on GT or who are not satisfied with their current treatment. This may contribute to the clear bimodal distribution in all groups, though this bimodal distribution has been described in literature in larger groups with different diseases. In addition, the relatively low sample size hindered calculation of the maximum acceptable risk per disease group and subgroup analysis, despite all groups being a reasonable size for such rare diseases.

Differences in the composition and perspective of each group may have influenced the results of this study. Participants with GD were markedly older and the views of young adults with GD (18–40 years old) are not represented in the current study, despite efforts to include them. Besides, the MPS III group differed from the others due to their perspective as a parent instead of a patient. To our knowledge, there is no previous literature on the effect of proxy decision making on risk tolerance. However, in a previous study on the experiences of parents of people with MPS III, the authors concluded “Most parents expressed a willingness to ‘try anything’, including treatments with potentially high risk profiles, to maintain their child’s current state” [57]. However, as expressed in focus groups in this population, this high risk tolerance should not be interpreted as a disregard for the attributes tested in the present survey [19]. Rather, it is a sign of desperation stemming from the dire current situation in which there are no therapy options for a disease with a profound impact on each affected person and their family [19].

By presenting these patient-driven research questions using the PTT method and evidence-based attributes and levels, the present study provides a unique, and to the best of our knowledge first, perspective on preferences of people affected by LSDs regarding GT. Therefore, despite some limitations, this study allows conclusions about preferences of people with LSDs concerning GT that closely resemble both the context and choices made in a clinical setting. An open question remains what the exact considerations are behind different choices of respondents, for example, if a respondent chooses current therapy regardless of the attribute, what drives that choice? This could be elucidated by a future study combining a decision aid based on the attributes of this survey with a semi-structured interview during completion of the decision aid. Given the nature of the attributes that emerged from the focus groups, namely personal ethical considerations, and considerations specific to the mode of administration of GT, we consider it crucial that the development of new GT methods includes surveys such as those presented in this study prior to inclusion of trial participants. This will ensure that time- and resource-intensive treatment developments align with preferences of the target group. It also allows development of therapies such as GT to start in the population with the highest unmet need, and offers starting points for patient reported outcome measures to assess in clinical trials. Other aspects must also be considered to ensure access to GT, such as the pricing of such therapies, [2, 58] This was most recently exemplified by exclusion of public reimbursement of atidarsagene autostemcel (Libmeldy®; Orchard Therapeutics BV) for Metachromatic Leukodystrophy in the Netherlands due to its high price [59]. Only by involving all stakeholders in the development of GTs, these can fulfill their potential for people affected by rare, monogenetic diseases.

In conclusion, this study demonstrates the heterogeneity of patient preferences in different LSDs, and thereby highlights the importance of involvement of patient preferences before and during the development process of new treatment modalities such as gene therapy for rare diseases, to ensure that innovative therapies align with the wishes and needs of people affected by these diseases.

## Supplementary Information

Additional file 1.

Additional file 2.

Additional file 3.

Additional file 4.

Additional file 5.

Additional file 6.

## Data Availability

The data that support the findings of this study are not openly available due to reasons of sensitivity. The data used and/or analyzed during the current study are, however, available from the corresponding author and with permission of the participants upon reasonable request.

## Abbreviations

BMQ-S: Specific part of the Beliefs in Medicine Questionnaire
EMA: European Medicines Agency
ERT: Enzyme replacement therapy
FD: Fabry disease
FDA: US Food and Drug Administration
GD: Gaucher disease type 1
GT: Gene therapy
LSD: Lysosomal storage disease
MPS III: Mucopolysaccharidosis type III A/B
PTT: Probabilistic threshold technique
RR: Response rate
SE: Side effect
SRT: Substrate reduction therapy
