# Supplementary material for: Different diseases, different needs: Patient preferences for gene therapy in lysosomal storage disorders, a probabilistic threshold technique survey
Source: Orphanet J Rare Dis. 2024 Oct 3;19:367. doi: 10.1186/s13023-024-03371-y (PMC11451020; doi:10.1186/s13023-024-03371-y)
Supplement: Supplementary file 1 — Additional file 1. [file 13023_2024_3371_MOESM1_ESM.pdf]

PTT survey as encountered by participants

Please note that the original survey was programmed in an online environment, therefore this printed version of the survey does not:

- include the technical online features (e.g. hover features) that were used to flag and highlight information for respondents;
- show the inserted loops in the survey, meaning that all questions are included in the printed version of the survey, while respondents were routed to skip questions depending on their answers to previous questions (e.g. regarding current therapy);
- include the PTT task in the way exactly shown to respondents as the attribute levels presented were dependent on respondents' answers to previous choice of the same task.

The survey was distributed in Dutch, and since much of the nuanced language would be lost in translation the Dutch original is presented here. The authors are available for help with translating and understanding the survey.

PTT survey for participants with Gaucher disease

Volgende

Doel van dit onderzoek

Uit groepsgesprekken en interviews met patiënten met de ziekte van Gaucher is gebleken dat er verschillende factoren zijn die maken of patiënten in de toekomst gentherapie zouden willen ontvangen. De belangrijkste factoren zijn eventuele bijwerkingen van therapie en de te verwachten werkzaamheid (effectiviteit).

Met deze vragenlijst willen wij – samen met VKS, de patiëntenvereniging voor volwassenen en kinderen met een erfelijke stofwisselingsziekte – onderzoeken welke patiënten wanneer gentherapie zouden willen ontvangen als zij hiervoor (in studieverband) in aanmerking zouden komen.

Om te kunnen onderzoeken in hoeverre deze factoren invloed hebben op de keuzes van patiënten stellen we u vragen over:
- uw huidige situatie
- gentherapie
- geneesmiddelen in het algemeen

Wij vragen u om aan te geven of u onder verschillende omstandigheden zou kiezen voor behandeling met gentherapie of uw huidige therapie. Voor deze vragenlijst gaan we ervan uit dat u behandeld kunt worden met gentherapie. Of dit in de toekomst daadwerkelijk zo zal zijn is niet bekend.

U kunt de vragenlijst op elk moment sluiten en later op dezelfde plek verdergaan met invullen. Tijdens het invullen van de vragenlijst kunt u niet terug naar vorige vragen. Als u bij het invullen van de vragenlijst hulp wilt of tegen iets aanloopt, sluit de vragenlijst en stuur een mail naar een van de onderzoekers, Ellie Corazolla (e.m.corazolla@amsterdamumc.nl), met de volgende informatie:
- Waar loopt u tegenaan?
- Wilt u via de mail of telefonisch geholpen worden?
- Als u telefonisch contact wenst: Wanneer bent u in de komende dagen bereikbaar? En op welk telefoonnummer?

Amsterdam UMC
Universitair Medische Centra

Volgende

Achtergrondinformatie

Bij de ziekte van Gaucher zit er een fout in het erfelijk (genetisch) materiaal (DNA) waardoor een bepaald eiwit (ook wel enzym: glucocerebrosidase) niet of niet goed werkt. Daardoor stapelt er een vetachtige stof in de cellen.

Gentherapie is een vorm van therapie waarbij 'nieuw' erfelijk materiaal in cellen wordt ingebracht. Het doel hiervan is om een stukje DNA toe te voegen zodat er een goed werkend enzym gevormd kan worden. Grofweg zijn er twee soorten gentherapie (zie figuur).

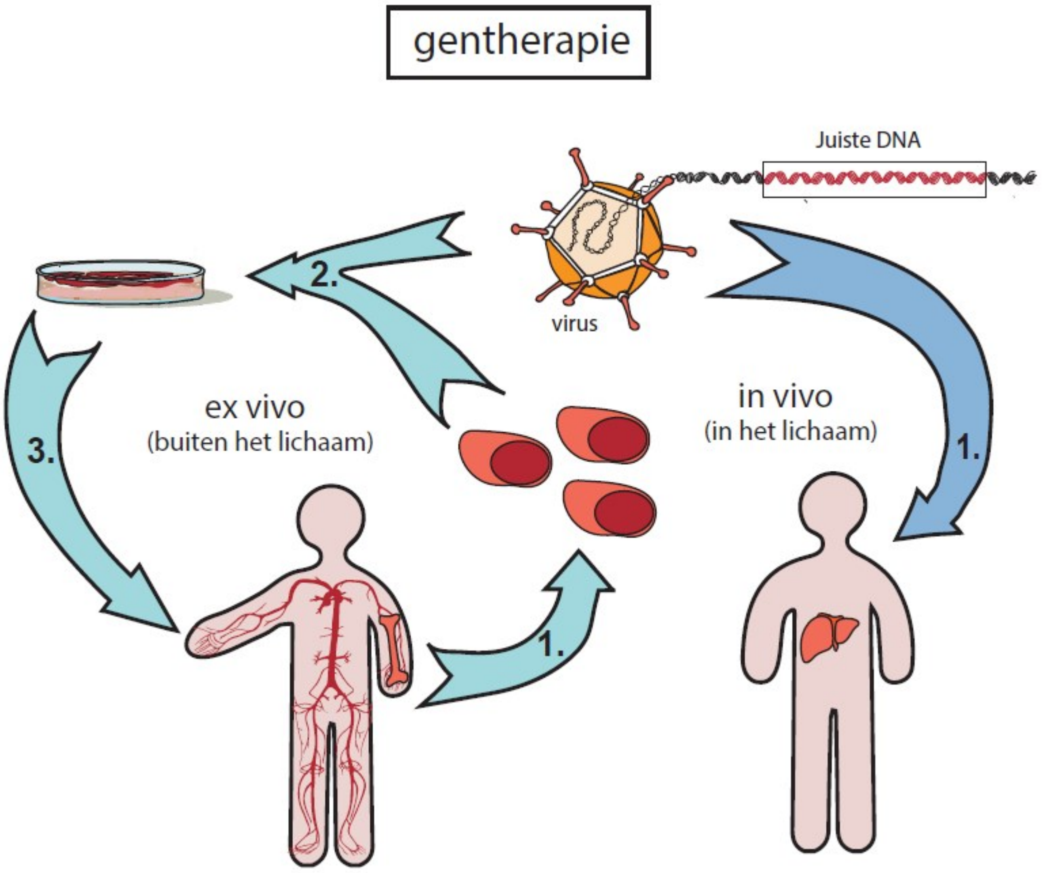

Volgende

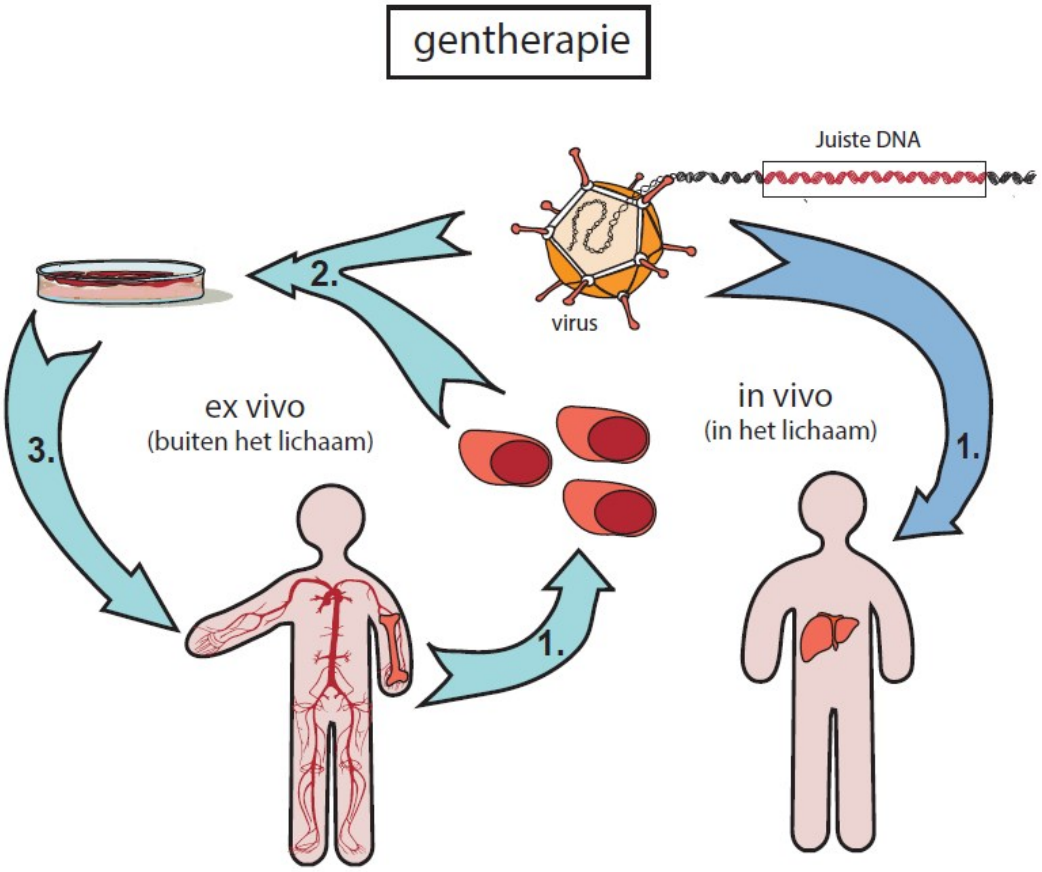

Bij de ene soort ("<u>in vivo</u>") wordt een virusonderdeel waar je niet ziek van wordt in het lichaam gespoten (1.). Dit virusonderdeel heeft het 'nieuwe' DNA bij zich en bouwt dit in bepaalde lichaamscellen in.

- <u>Voordeel van deze vorm van gentherapie</u>: het is een behandeling die gericht in het brein gegeven wordt
- <u>Nadeel van deze vorm van gentherapie</u>: een deel van de mensen hebben het virus al eens 'gehad'. Het lichaam zal het daarom zal herkennen en opruimen. Óf dit bij mensen inderdaad zo is, moet nog worden onderzocht. Áls dit zo is, dan is het de vraag wat het effect van dit 'opruimen' op de werkzaamheid van de therapie is.

De andere soort ("<u>ex vivo</u>") vindt in meerdere stappen plaats:
1. Er worden beenmergcellen uit het lichaam van patiënten gehaald via een bloedafname.
2. Buiten het lichaam worden de cellen behandeld met gentherapie.
3. Daarna worden de behandelde cellen teruggeplaatst in het lichaam.

- <u>Voordelen van deze vorm van gentherapie</u>: er is controle over de hoeveelheid gentherapie waar de specifieke cellen aan worden blootgesteld. Daarnaast kan het lichaam het DNA mogelijk minder makkelijk 'opruimen' omdat het al in de cellen zit.
- <u>Nadelen van deze vorm van gentherapie</u>: er moet eerst ruimte in het beenmerg komen om de 'nieuwe' cellen te laten uitgroeien voordat de cellen teruggeplaatst kunnen worden. Daarom moet er een milde vorm van chemotherapie worden gegeven. Deze behandeling is kortdurend. Daarnaast is er nog geen zekerheid of deze vorm van gentherapie tot blijvende

enzymproductie leidt.

Op dit moment wordt er onderzoek gedaan naar beide soorten gentherapie voor de ziekte van Gaucher. Bij de vragen die we u zullen stellen maken we geen onderscheid tussen de verschillende vormen.

Volgende

Achtergrondinformatie

In welke leeftijdcategorie valt u?

- ◯ 18 jaar of jonger
- ◯ Tussen de 18 - 29 jaar
- ◯ Tussen 30 - 39 jaar
- ◯ Tussen 40 - 49 jaar
- ◯ Tussen 50 - 59 jaar
- ◯ Tussen 60 - 69 jaar
- ◯ Tussen 70 - 79 jaar
- ◯ 80 jaar of ouder

Wat is uw geslacht

- ◯ Man
- ◯ Vrouw
- ◯ Anders

Gebruikt u op dit moment medicijnen voor de ziekte van Gaucher (hierbij wordt bedoeld: **enzymtherapie** en/of **substraatreductietherapie** (Eliglustat/Cerdelga©); het gaat hier **niet** om bijvoorbeeld pijnstillers)?

- ◯ Ja
- ◯ Nee

Volgende

Welk medicijn gebruikt u?

- ◯ Enzymtherapie: Imiglucerase (Cerezyme®)
- ◯ Enzymtherapie: Velaglucerase alfa (VPRIV®)
- ◯ Substraatreductietherapie: Eliglustat (Cerdelga®)

Volgende

Keuzetaken

We vragen u straks steeds een keuze te maken uit twee behandelopties; uw huidige therapie of gentherapie. De keuzes zullen erg op elkaar lijken, toch zijn er kleine verschillen. Het is belangrijk voor ons dat u de informatie op de volgende pagina's goed leest en alle keuzetaken invult, we zullen hier de verschillende kenmerken van de behandelingen toelichten.

We vragen bij de keuzetaken om uw persoonlijke mening, er zijn dus geen goede of foute antwoorden/keuzes.

Het is belangrijk om te vermelden dat het mogelijk is om eerder gebruikte therapie weer te hervatten als gentherapie niet goed werkt.

Volgende

Uitleg over de werkzaamheid van de behandeling

We gaan ervan uit dat de gentherapie even goed werkt als uw huidige therapie. Gentherapie is een eenmalige behandeling; bij uw huidige behandeling is het noodzakelijk om langdurig tweewekelijkse infusen te krijgen of dagelijks tabletten te slikken. Er zijn medicijnen die niet in combinatie met uw huidige medicatie kunnen worden gebruikt. Het ondergaan van gentherapie kan gepaard gaan met milde en/of ernstige bijwerkingen. Ook bestaat er een kans dat er extra medicatie gebruikt moet worden. Tot slot is er nog onzekerheid over of gentherapie goed blijft werken op de lange termijn. In de vragenlijst stellen we u vragen die steeds betrekking hebben op een van deze aspecten. We vragen u bij elke vraag een keuze te maken tussen gentherapie en uw huidige therapie.

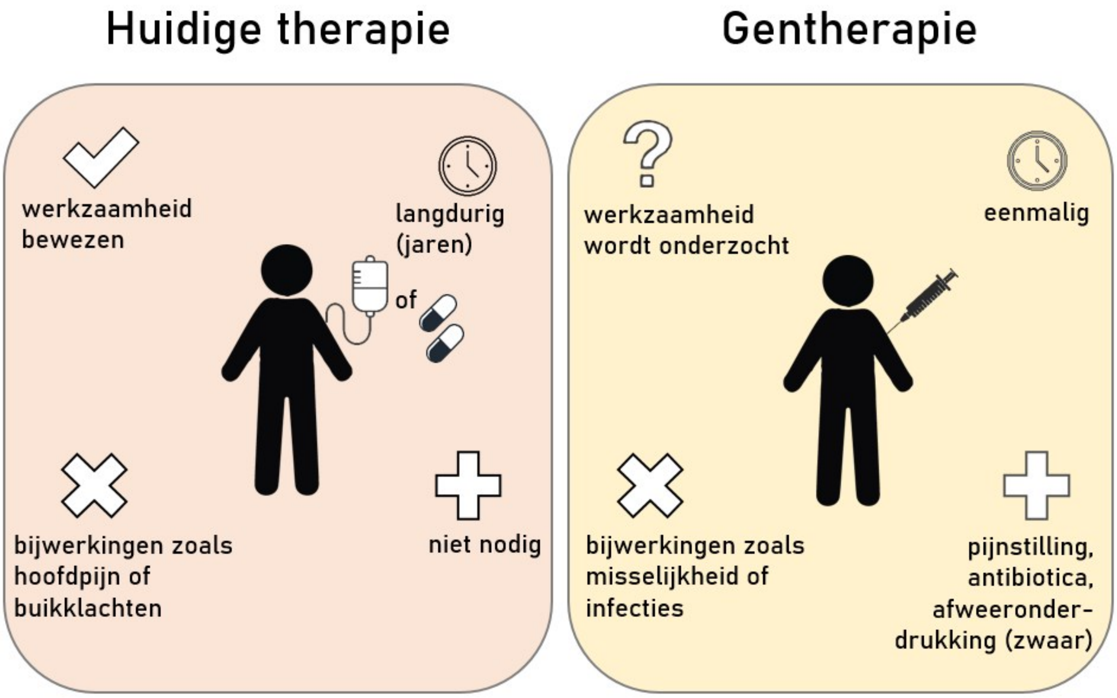

Volgende

Uitleg over mogelijke bijwerkingen van de behandelingen

Milde bijwerkingen: bij het gebruik van enzymtherapie of gentherapie kunnen milde bijwerkingen optreden. Deze bijwerkingen duren kort, zijn zonder blijvende gevolgen en er is geen ziekenhuisopname nodig.

<u>Voor enzymtherapie</u> (Imiglucerase/Cerezyme® of Velaglucerase/VPRIV®)
- Blauwe plekken of bloeduitstortingen - Bij langdurig gebruik (jaren) kan het aanprikken van de vaten zeer moeilijk worden

<u>Voor substraatremming</u> (Eliglustat/Cerdelga®)
- hoofdpijn
- buikpijn
- diarree
- gewrichtspijn
- vermoeidheid

<u>Voor gentherapie</u>:
- het krijgen van een blauwe plek of bloeduitstorting
- tijdelijk algeheel niet lekker zijn (malaise)
- milde griepachtige klachten
- kortdurende misselijkheid of overgeven
- buikpijn of diarree
- ongevaarlijke infecties
- bloedarmoede
- pijnlijke ontstekingen van het mondslijmvlies
- koorts

Ernstige bijwerkingen: bij het gebruik van enzymtherapie of gentherapie kunnen ernstige bijwerkingen optreden. Deze bijwerkingen hebben een ziekenhuisopname tot gevolg.

<u>Voor enzymtherapie</u>:
- allergische reacties
- zenuwpijn
- flauwvallen

<u>Voor gentherapie</u>: ziekenhuisopnames vanwege bijvoorbeeld
- zeer hoge koorts
- ernstige infecties

Bij gentherapie voor aandoeningen die vergelijkbaar zijn met de ziekte van Gaucher overlijden mensen niet aan de gevolgen van de gentherapiebehandeling.

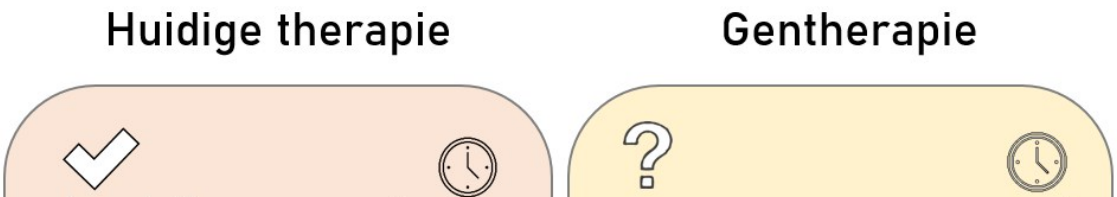

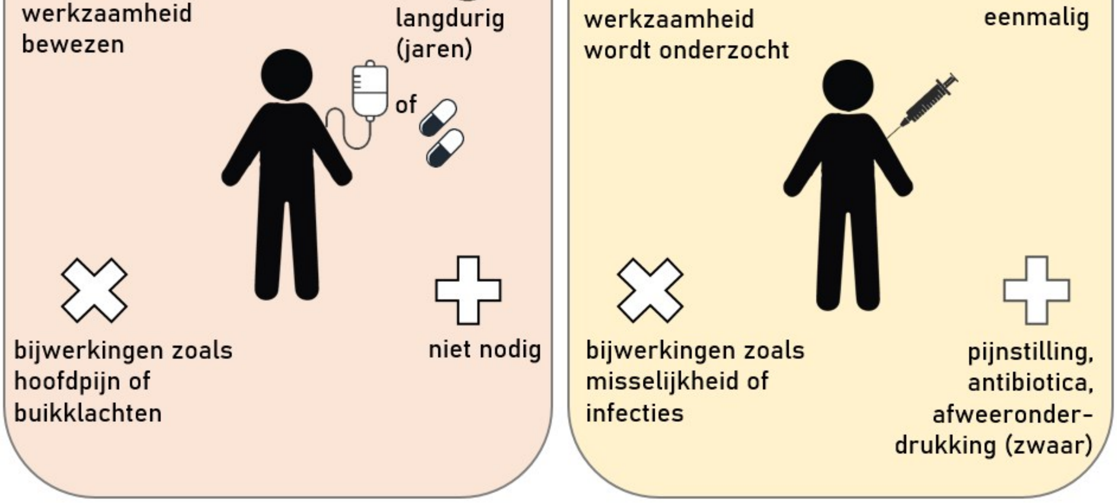
werkzaamheid
bewezen
langdurig
(jaren)
of
bijwerkingen zoals
hoofdpijn of
buikklachten
niet nodig
werkzaamheid
wordt onderzocht
eenmalig
bijwerkingen zoals
misselijkheid of
infecties
pijnstilling,
antibiotica,
afweeronder-
drukking (zwaar)

Volgende

Uitleg over aanvullende medicatie en behandelfrequentie van de behandelingen.

Aanvullende medicatie: het kan nodig zijn om de bijwerkingen van enzymtherapie of gentherapie te onderdrukken met medicatie. Dit kan zowel kortdurend als langdurig zijn. Met deze medicatie bedoelen we geen medicijnen die gebruikt worden om eventuele al bestaande problemen door de ziekte te behandelen (bijvoorbeeld een middel dat nodig is vanwege botpijn), maar medicijnen die verschijnselen die de therapie veroorzaakt tegengaan (bijvoorbeeld misselijkheid door de gentherapie).

Voor uw huidige therapie is aanvullende medicatie over het algemeen niet nodig.

Voor gentherapie: Dit kan gaan om een immuunsysteem onderdrukkend middel, zoals prednison. Dat wordt kortdurend (weken tot maanden) gegeven om een afweerreactie van het lichaam tegen het (ongevaarlijke) virusonderdeel tegen te gaan. Daarnaast kan medicatie nodig zijn om bijwerkingen te verhelpen, zoals een antibioticakuur, pijnstilling, of middelen tegen misselijkheid.

Behandelfrequentie: voor uw huidige therapie geldt dat u langdurig (jaren) elke twee tot vier weken een infuus krijgt of dagelijks een pil slikt, terwijl voor gentherapie geldt dat de behandeling eenmalig is.

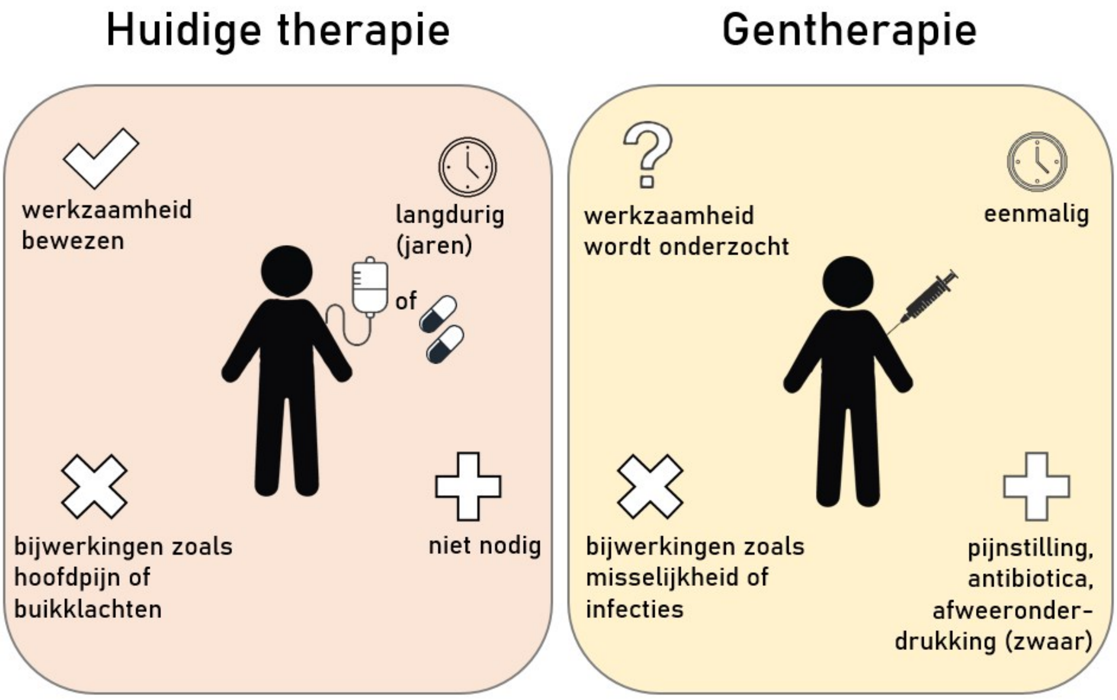

Volgende

Uitleg over waarschijnlijkheid en kansen

Voor verschillende kenmerken van behandelingen wordt straks geproken over 'waarschijnlijkheid'. Hiermee bedoelen we de kans dat de behandeling milde of ernstige bijwerkingen tot gevolg heeft of de kans dat u aanvullende medicatie zou moeten nemen. Er worden verschillende kansen aan u gepresenteerd. Wanneer de waarschijnlijkheid (oftewel kans) op een bijwerking 40% is dan zullen 40 van elke 100 mensen die het medicijn nemen bijwerkingen krijgen terwijl 60 van de 100 mensen die het medicijn nemen geen bijwerkingen zullen ervaren. Dit ziet er als volgt uit:

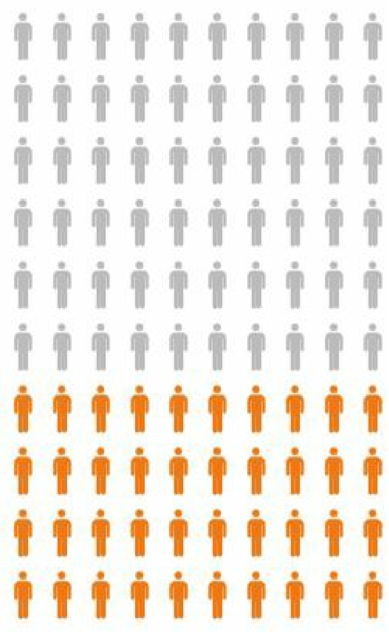

Volgende

Het invullen van de keuzetaken

We vragen u zo meerdere keren een keuze te maken uit twee behandelopties; uw huidige therapie of gentherapie.

De behandelingen zullen erg op elkaar lijken, toch zijn er kleine verschillen.

De keuzes zullen eruit zien zoals in het plaatje hieronder. Aan de linkerkant ziet u de eigenschappen van de behandelingen staan. Wanneer u straks de keuzetaken gaat invullen kunt u hier met uw muis op gaat staan, om de uitleg die hierbij hoort nogmaals lezen.

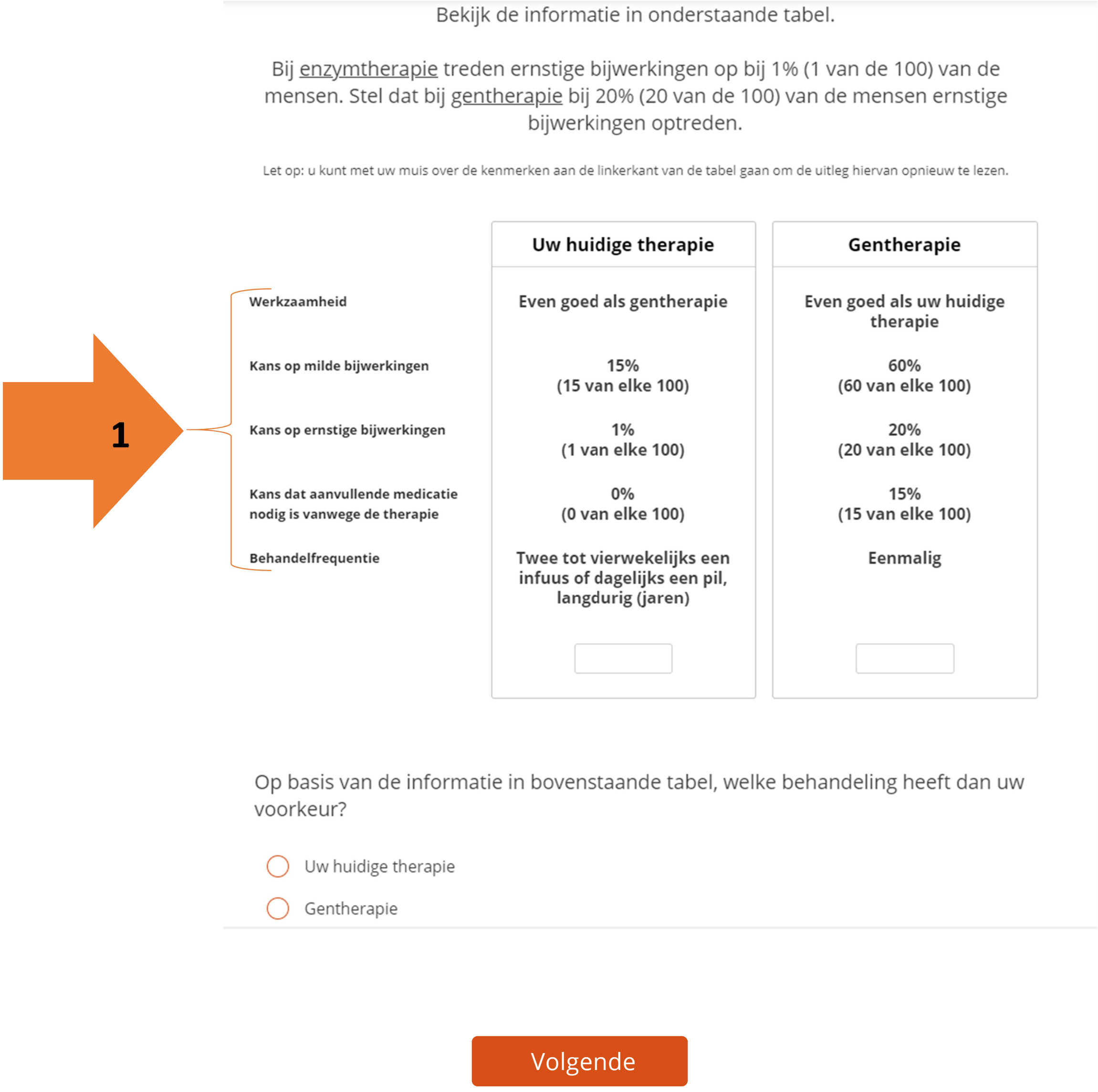
Bekijk de informatie in onderstaande tabel.

Bij enzymtherapie treden ernstige bijwerkingen op bij 1% (1 van de 100) van de mensen. Stel dat bij gentherapie bij 20% (20 van de 100) van de mensen ernstige bijwerkingen optreden.

Let op: u kunt met uw muis over de kenmerken aan de linkerkant van de tabel gaan om de uitleg hiervan opnieuw te lezen.

1

	Uw huidige therapie	Gentherapie
Werkzaamheid	Even goed als gentherapie	Even goed als uw huidige therapie
Kans op milde bijwerkingen	15% (15 van elke 100)	60% (60 van elke 100)
Kans op ernstige bijwerkingen	1% (1 van elke 100)	20% (20 van elke 100)
Kans dat aanvullende medicatie nodig is vanwege de therapie	0% (0 van elke 100)	15% (15 van elke 100)
Behandelfrequentie	Twee tot vierwekelijks een infuus of dagelijks een pil, langdurig (jaren)	Eenmalig

Op basis van de informatie in bovenstaande tabel, welke behandeling heeft dan uw voorkeur?

- Uw huidige therapie
- Gentherapie

Volgende

In het midden van de taak ziet u de twee behandelingen.

Beide behandelingen hebben net iets andere niveaus van hun eigenschappen.

In het onderstaande voorbeeld werken beide behandelingen even goed. U heeft bij de gentherapie meer kans op ernstige bijwerkingen (namelijk 20% in plaats van 15% voor uw huidige therapie), maar gentherapie is een eenmalige behandeling terwijl uw huidige therapie meerdere jaren tweewekelijks moet worden toegediend.

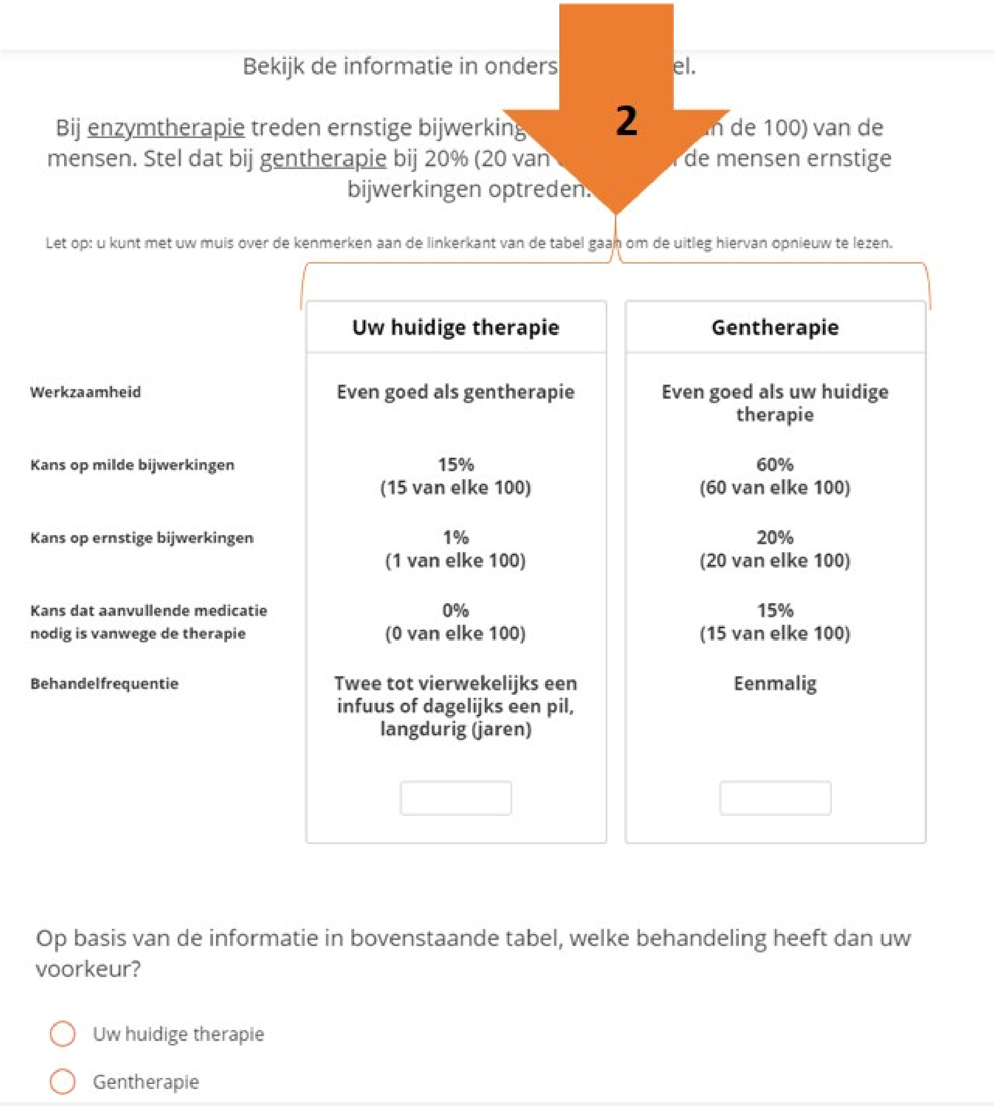

Bekijk de informatie in onders[illegible]el.

Bij enzymtherapie treden ernstige bijwerking[illegible]n de 100) van de mensen. Stel dat bij gentherapie bij 20% (20 van [illegible] de mensen ernstige bijwerkingen optreden.

Let op: u kunt met uw muis over de kenmerken aan de linkerkant van de tabel gaan om de uitleg hiervan opnieuw te lezen.

	Uw huidige therapie	Gentherapie
Werkzaamheid	Even goed als gentherapie	Even goed als uw huidige therapie
Kans op milde bijwerkingen	15% (15 van elke 100)	60% (60 van elke 100)
Kans op ernstige bijwerkingen	1% (1 van elke 100)	20% (20 van elke 100)
Kans dat aanvullende medicatie nodig is vanwege de therapie	0% (0 van elke 100)	15% (15 van elke 100)
Behandelfrequentie	Twee tot vierwekelijks een infuus of dagelijks een pil, langdurig (jaren)	Eenmalig

Op basis van de informatie in bovenstaande tabel, welke behandeling heeft dan uw voorkeur?

- ◯ Uw huidige therapie
- ◯ Gentherapie

Volgende

Vervolgens is het aan u om een afweging te maken tussen deze behandelingen en hun eigenschappen en een keuze te maken.

U kunt uw keuze bevestigen door:
1. In de tabel op de therapie van uw voorkeur te klikken
2. In de vraag onder de tabel dezelfde therapie aan te klikken

Het is belangrijk dat u uw keuze in de tabel nogmaals bevestigt in de vraag onder elke tabel zoals in het voorbeeld hieronder waar de patient voor gentherapie heeft gekozen.

Bekijk de informatie in onderstaande tabel.

Bij enzymtherapie treden ernstige bijwerkingen op bij 1% (1 van de 100) van de mensen. Stel dat bij gentherapie bij 20% (20 van de 100) van de mensen ernstige bijwerkingen optreden.

Let op: u kunt met uw muis over de kenmerken aan de linkerkant van de tabel gaan om de uitleg hiervan opnieuw te lezen.

	**Uw huidige therapie**	**Gentherapie**
**Werkzaamheid**	Even goed als gentherapie	Even goed als uw huidige therapie
**Kans op milde bijwerkingen**	15% (15 van elke 100)	60% (60 van elke 100)
**Kans op ernstige bijwerkingen**	1% (1 van elke 100)	20% (20 van elke 100)
**Kans dat aanvullende medicatie nodig is vanwege de therapie**	0% (0 van elke 100)	15% (15 van elke 100)
**Behandelfrequentie**	Twee tot vierwekelijks een infuus of dagelijks een pil, langdurig (jaren)	Eenmalig

3

Op basis van de informatie in bovenstaande tabel, welke behande[illegible] dan uw voorkeur?

3

- ○ Uw huidige therapie
- ● Gentherapie

Volgende

Deel 1: Milde bijwerkingen

Bij dit onderdeel van de vragenlijst gaat het om milde bijwerkingen die kunnen optreden bij het gebruik van uw huidige therapie of gentherapie. Deze bijwerkingen duren kort, zijn zonder blijvende gevolgen en er is geen ziekenhuisopname nodig.

Volgende

Bekijk de informatie in onderstaande tabel.

Bij uw huidige therapie treden milde bijwerkingen op bij 15% (15 van de 100) van de mensen. Stel dat bij gentherapie bij 60% (60 van de 100) van de mensen milde bijwerkingen optreden.

Let op: u kunt met uw muis over de kenmerken aan de linkerkant van de tabel gaan om de uitleg hiervan opnieuw te lezen.

	Uw huidige therapie	Gentherapie
**Werkzaamheid**	**Even goed als gentherapie**	**Even goed als uw huidige therapie**
**Kans op milde bijwerkingen**	**15%<br>(15 van elke 100)**	**60%<br>(60 van elke 100)**
**Kans op ernstige bijwerkingen**	**1%<br>(1 van elke 100)**	**20%<br>(20 van elke 100)**
**Kans dat aanvullende medicatie nodig is vanwege de therapie**	**0%<br>(0 van elke 100)**	**15%<br>(15 van elke 100)**
**Behandelfrequentie**	**Twee tot vierwekelijks een infuus of dagelijks een pil, langdurig (jaren)**	**Eenmalig**

Op basis van de informatie in bovenstaande tabel, welke behandeling heeft dan uw voorkeur?

- ○ Uw huidige therapie
- ○ Gentherapie

Volgende

Bekijk de informatie in onderstaande tabel.

Bij uw huidige therapie treden milde bijwerkingen op bij 15% (15 van de 100) van de mensen. Stel dat bij gentherapie bij 40% (40 van de 100) van de mensen milde bijwerkingen optreden.

Let op: u kunt met uw muis over de kenmerken aan de linkerkant van de tabel gaan om de uitleg hiervan opnieuw te lezen.

	Uw huidige therapie	Gentherapie
**Werkzaamheid**	**Even goed als gentherapie**	**Even goed als uw huidige therapie**
**Kans op milde bijwerkingen**	**15%<br>(15 van elke 100)**	**40%<br>(40 van elke 100)**
**Kans op ernstige bijwerkingen**	**1%<br>(1 van elke 100)**	**20%<br>(20 van elke 100)**
**Kans dat aanvullende medicatie nodig is vanwege de therapie**	**0%<br>(0 van elke 100)**	**15%<br>(15 van elke 100)**
**Behandelfrequentie**	**Twee tot vierwekelijks een infuus of dagelijks een pil, langdurig (jaren)**	**Eenmalig**

Op basis van de informatie in bovenstaande tabel, welke behandeling heeft dan uw voorkeur?

- ◯ Uw huidige therapie
- ◯ Gentherapie

Volgende

Bekijk de informatie in onderstaande tabel.

Bij uw huidige therapie treden milde bijwerkingen op bij 15% (15 van de 100) van de mensen. Stel dat bij gentherapie bij 50% (50 van de 100) van de mensen milde bijwerkingen optreden.

Let op: u kunt met uw muis over de kenmerken aan de linkerkant van de tabel gaan om de uitleg hiervan opnieuw te lezen.

	Uw huidige therapie	Gentherapie
**Werkzaamheid**	**Even goed als gentherapie**	**Even goed als uw huidige therapie**
**Kans op milde bijwerkingen**	**15% (15 van elke 100)**	**50% (50 van elke 100)**
**Kans op ernstige bijwerkingen**	**1% (1 van elke 100)**	**20% (20 van elke 100)**
**Kans dat aanvullende medicatie nodig is vanwege de therapie**	**0% (0 van elke 100)**	**15% (15 van elke 100)**
**Behandelfrequentie**	**Twee tot vierwekelijks een infuus of dagelijks een pil, langdurig (jaren)**	**Eenmalig**

Op basis van de informatie in bovenstaande tabel, welke behandeling heeft dan uw voorkeur?

- ◯ Uw huidige therapie
- ◯ Gentherapie

Volgende

Bekijk de informatie in onderstaande tabel.

Bij uw huidige therapie treden milde bijwerkingen op bij 15% (15 van de 100) van de mensen. Stel dat bij gentherapie bij 80% (80 van de 100) van de mensen milde bijwerkingen optreden.

Let op: u kunt met uw muis over de kenmerken aan de linkerkant van de tabel gaan om de uitleg hiervan opnieuw te lezen.

	**Uw huidige therapie**	**Gentherapie**
**Werkzaamheid**	**Even goed als gentherapie**	**Even goed als uw huidige therapie**
**Kans op milde bijwerkingen**	**15%<br>(15 van elke 100)**	**80%<br>(80 van elke 100)**
**Kans op ernstige bijwerkingen**	**1%<br>(1 van elke 100)**	**20%<br>(20 van elke 100)**
**Kans dat aanvullende medicatie nodig is vanwege de therapie**	**0%<br>(0 van elke 100)**	**15%<br>(15 van elke 100)**
**Behandelfrequentie**	**Twee tot vierwekelijks een infuus of dagelijks een pil, langdurig (jaren)**	**Eenmalig**

Op basis van de informatie in bovenstaande tabel, welke behandeling heeft dan uw voorkeur?

- ○ Uw huidige therapie
- ○ Gentherapie

Volgende

Bekijk de informatie in onderstaande tabel.

Bij uw huidige therapie treden milde bijwerkingen op bij 15% (15 van de 100) van de mensen. Stel dat bij gentherapie bij 70% (70 van de 100) van de mensen milde bijwerkingen optreden.

Let op: u kunt met uw muis over de kenmerken aan de linkerkant van de tabel gaan om de uitleg hiervan opnieuw te lezen.

	Uw huidige therapie	Gentherapie
**Werkzaamheid**	**Even goed als gentherapie**	**Even goed als uw huidige therapie**
**Kans op milde bijwerkingen**	**15% (15 van elke 100)**	**70% (70 van elke 100)**
**Kans op ernstige bijwerkingen**	**1% (1 van elke 100)**	**20% (20 van elke 100)**
**Kans dat aanvullende medicatie nodig is vanwege de therapie**	**0% (0 van elke 100)**	**15% (15 van elke 100)**
**Behandelfrequentie**	**Twee tot vierwekelijks een infuus of dagelijks een pil, langdurig (jaren)**	**Eenmalig**

Op basis van de informatie in bovenstaande tabel, welke behandeling heeft dan uw voorkeur?

- ○ Uw huidige therapie
- ○ Gentherapie

Volgende

U heeft net aangegeven dat u voor uw huidige therapie zou kiezen als de kans op milde bijwerkingen bij gentherapie 40% is.

Wat is de hoogste kans (in %) op milde bijwerkingen waarbij u toch gentherapie zou kiezen? (let op: dit is dus altijd lager dan 40, maar kan ook 0 zijn indien u bijvoorbeeld helemaal geen gentherapie wil)

Volgende

U heeft net aangegeven dat u voor gentherapie zou kiezen als de kans op milde bijwerkingen 80% is.

Wat is de hoogste kans (in %) op milde bijwerkingen waarbij u nog gentherapie zou kiezen? (let op: dit is dus altijd hoger dan 80, maar kan ook 100 zijn indien u altijd voor gentherapie zou kiezen)

Volgende

Heeft u op dit moment of in het verleden een of meerdere van de **milde bijwerkingen** (gehad) die bij uw huidige therapie kunnen voorkomen?

- ◯ Ja
- ◯ Nee

Volgende

Deel 2: Ernstige bijwerkingen

Bij dit onderdeel van de vragenlijst gaat het om ernstige bijwerkingen die kunnen optreden bij het gebruik van uw huidige of bij gentherapie. Deze bijwerkingen hebben een ziekenhuisopname tot gevolg.

Volgende

Bekijk de informatie in onderstaande tabel.

Bij enzymtherapie treden ernstige bijwerkingen op bij 1% (1 van de 100) van de mensen. Stel dat bij gentherapie bij 20% (20 van de 100) van de mensen ernstige bijwerkingen optreden.

Let op: u kunt met uw muis over de kenmerken aan de linkerkant van de tabel gaan om de uitleg hiervan opnieuw te lezen.

	**Uw huidige therapie**	**Gentherapie**
**Werkzaamheid**	**Even goed als gentherapie**	**Even goed als uw huidige therapie**
**Kans op milde bijwerkingen**	**15% (15 van elke 100)**	**60% (60 van elke 100)**
**Kans op ernstige bijwerkingen**	**1% (1 van elke 100)**	**20% (20 van elke 100)**
**Kans dat aanvullende medicatie nodig is vanwege de therapie**	**0% (0 van elke 100)**	**15% (15 van elke 100)**
**Behandelfrequentie**	**Twee tot vierwekelijks een infuus of dagelijks een pil, langdurig (jaren)**	**Eenmalig**

Op basis van de informatie in bovenstaande tabel, welke behandeling heeft dan uw voorkeur?

- ○ Uw huidige therapie
- ○ Gentherapie

Volgende

Bekijk de informatie in onderstaande tabel.

Bij uw huidige therapie treden ernstige bijwerkingen op bij 1% (1 van de 100) van de mensen. Stel dat bij gentherapie bij 5% (5 van de 100) van de mensen ernstige bijwerkingen optreden.

Let op: u kunt met uw muis over de kenmerken aan de linkerkant van de tabel gaan om de uitleg hiervan opnieuw te lezen.

	**Uw huidige therapie**	**Gentherapie**
**Werkzaamheid**	**Even goed als gentherapie**	**Even goed als uw huidige therapie**
**Kans op milde bijwerkingen**	**15% (15 van elke 100)**	**60% (60 van elke 100)**
**Kans op ernstige bijwerkingen**	**1% (1 van elke 100)**	**5% (5 van elke 100)**
**Kans dat aanvullende medicatie nodig is vanwege de therapie**	**0% (0 van elke 100)**	**15% (15 van elke 100)**
**Behandelfrequentie**	**Twee tot vierwekelijks een infuus of dagelijks een pil, langdurig (jaren)**	**Eenmalig**

Op basis van de informatie in bovenstaande tabel, welke behandeling heeft dan uw voorkeur?

- ○ Uw huidige therapie
- ○ Gentherapie

Volgende

Bekijk de informatie in onderstaande tabel.

Bij uw huidige therapie treden ernstige bijwerkingen op bij 1% (1 van de 100) van de mensen. Stel dat bij gentherapie bij 10% (10 van de 100) van de mensen ernstige bijwerkingen optreden.

Let op: u kunt met uw muis over de kenmerken aan de linkerkant van de tabel gaan om de uitleg hiervan opnieuw te lezen.

	Uw huidige therapie	Gentherapie
**Werkzaamheid**	**Even goed als gentherapie**	**Even goed als uw huidige therapie**
**Kans op milde bijwerkingen**	**15% (15 van elke 100)**	**60% (60 van elke 100)**
**Kans op ernstige bijwerkingen**	**1% (1 van elke 100)**	**10% (10 van elke 100)**
**Kans dat aanvullende medicatie nodig is vanwege de therapie**	**0% (0 van elke 100)**	**15% (15 van elke 100)**
**Behandelfrequentie**	**Twee tot vierwekelijks een infuus of dagelijks een pil, langdurig (jaren)**	**Eenmalig**

Op basis van de informatie in bovenstaande tabel, welke behandeling heeft dan uw voorkeur?

- ◯ Uw huidige therapie
- ◯ Gentherapie

Volgende

Bekijk de informatie in onderstaande tabel.

Bij uw huidige therapie treden ernstige bijwerkingen op bij 1% (1 van de 100) van de mensen. Stel dat bij gentherapie bij 80% (80 van de 100) van de mensen ernstige bijwerkingen optreden.

Let op: u kunt met uw muis over de kenmerken aan de linkerkant van de tabel gaan om de uitleg hiervan opnieuw te lezen.

	Uw huidige therapie	Gentherapie
**Werkzaamheid**	**Even goed als gentherapie**	**Even goed als uw huidige therapie**
**Kans op milde bijwerkingen**	**15% (15 van elke 100)**	**60% (60 van elke 100)**
**Kans op ernstige bijwerkingen**	**1% (1 van elke 100)**	**80% (80 van elke 100)**
**Kans dat aanvullende medicatie nodig is vanwege de therapie**	**0% (0 van elke 100)**	**15% (15 van elke 100)**
**Behandelfrequentie**	**Twee tot vierwekelijks een infuus of dagelijks een pil, langdurig (jaren)**	**Eenmalig**

Op basis van de informatie in bovenstaande tabel, welke behandeling heeft dan uw voorkeur?

- ◯ Uw huidige therapie
- ◯ Gentherapie

Volgende

Bekijk de informatie in onderstaande tabel.

Bij uw huidige therapie treden ernstige bijwerkingen op bij 1% (1 van de 100) van de mensen. Stel dat bij gentherapie bij 50% (50 van de 100) van de mensen ernstige bijwerkingen optreden.

Let op: u kunt met uw muis over de kenmerken aan de linkerkant van de tabel gaan om de uitleg hiervan opnieuw te lezen.

	Uw huidige therapie	Gentherapie
**Werkzaamheid**	**Even goed als gentherapie**	**Even goed als uw huidige therapie**
**Kans op milde bijwerkingen**	**15% (15 van elke 100)**	**60% (60 van elke 100)**
**Kans op ernstige bijwerkingen**	**1% (1 van elke 100)**	**50% (50 van elke 100)**
**Kans dat aanvullende medicatie nodig is vanwege de therapie**	**0% (0 van elke 100)**	**15% (15 van elke 100)**
**Behandelfrequentie**	**Twee tot vierwekelijks een infuus of dagelijks een pil, langdurig (jaren)**	**Eenmalig**

Op basis van de informatie in bovenstaande tabel, welke behandeling heeft dan uw voorkeur?

- ◯ Uw huidige therapie
- ◯ Gentherapie

Volgende

U heeft net aangegeven dat u voor uw huidige therapie zou kiezen als de kans op ernstige bijwerkingen bij gentherapie 5% is.

Wat is de hoogste kans (in %) op ernstige bijwerkingen waarbij u toch gentherapie zou kiezen? (let op: dit is dus altijd lager dan 5, maar kan ook 0 zijn indien u bijvoorbeeld helemaal geen gentherapie wil)

Volgende

U heeft net aangegeven dat u voor gentherapie zou kiezen als de kans op ernstige bijwerkingen 80% is.

Wat is de hoogste kans (in %) op ernstige bijwerkingen waarbij u nog gentherapie zou kiezen? (let op: dit is dus altijd hoger dan 80, maar kan ook 100 zijn indien u altijd voor gentherapie zou kiezen)

Volgende

Heeft u op dit moment of in het verleden een of meerdere van de **ernstige bijwerkingen** (gehad) die bij uw huidige therapie kunnen voorkomen?

- ◯ Ja
- ◯ Nee

Volgende

Deel 3: gebruik van aanvullende medicatie

Bij dit onderdeel van de vragenlijst gaat het om het gebruik van aanvullende medicatie, wat nodig kan zijn om de bijwerkingen van uw huidige therapie of gentherapie te onderdrukken. Dit kan zowel kortdurend als langdurig zijn.

Volgende

Bekijk de informatie in onderstaande tabel.

Bij uw huidige therapie is het gebruik van aanvullende medicatie nodig bij 0% (0 van de 100) van de mensen. Stel dat bij gentherapie bij 15% (15 van de 100) van de mensen aanvullende medicatie nodig zou zijn.

Let op: u kunt met uw muis over de kenmerken aan de linkerkant van de tabel gaan om de uitleg hiervan opnieuw te lezen.

	**Uw huidige therapie**	**Gentherapie**
**Werkzaamheid**	**Even goed als gentherapie**	**Even goed als uw huidige therapie**
**Kans op milde bijwerkingen**	**15% (15 van elke 100)**	**60% (60 van elke 100)**
**Kans op ernstige bijwerkingen**	**1% (1 van elke 100)**	**20% (20 van elke 100)**
**Kans dat aanvullende medicatie nodig is vanwege de therapie**	**0% (0 van elke 100)**	**15% (15 van elke 100)**
**Behandelfrequentie**	**Twee tot vierwekelijks een infuus of dagelijks een pil, langdurig (jaren)**	**Eenmalig**

Op basis van de informatie in bovenstaande tabel, welke behandeling heeft dan uw voorkeur?

- ◯ Uw huidige therapie
- ◯ Gentherapie

Volgende

Bekijk de informatie in onderstaande tabel.

Bij uw huidige therapie is het gebruik van aanvullende medicatie nodig bij 0% (0 van de 100) van de mensen. Stel dat bij gentherapie bij 5% (5 van de 100) van de mensen aanvullende medicatie nodig zou zijn.

Let op: u kunt met uw muis over de kenmerken aan de linkerkant van de tabel gaan om de uitleg hiervan opnieuw te lezen.

	Uw huidige therapie	Gentherapie
**Werkzaamheid**	**Even goed als gentherapie**	**Even goed als uw huidige therapie**
**Kans op milde bijwerkingen**	**15%<br>(15 van elke 100)**	**60%<br>(60 van elke 100)**
**Kans op ernstige bijwerkingen**	**1%<br>(1 van elke 100)**	**20%<br>(20 van elke 100)**
**Kans dat aanvullende medicatie nodig is vanwege de therapie**	**0%<br>(0 van elke 100)**	**5%<br>(5 van elke 100)**
**Behandelfrequentie**	**Twee tot vierwekelijks een infuus of dagelijks een pil, langdurig (jaren)**	**Eenmalig**

Op basis van de informatie in bovenstaande tabel, welke behandeling heeft dan uw voorkeur?

- ◯ Uw huidige therapie
- ◯ Gentherapie

Volgende

Bekijk de informatie in onderstaande tabel.

Bij uw huidige therapie is het gebruik van aanvullende medicatie nodig bij 0% (0 van de 100) van de mensen. Stel dat bij gentherapie bij 10% (10 van de 100) van de mensen aanvullende medicatie nodig zou zijn.

Let op: u kunt met uw muis over de kenmerken aan de linkerkant van de tabel gaan om de uitleg hiervan opnieuw te lezen.

	**Uw huidige therapie**	**Gentherapie**
**Werkzaamheid**	**Even goed als gentherapie**	**Even goed als uw huidige therapie**
**Kans op milde bijwerkingen**	**15%<br>(15 van elke 100)**	**60%<br>(60 van elke 100)**
**Kans op ernstige bijwerkingen**	**1%<br>(1 van elke 100)**	**20%<br>(20 van elke 100)**
**Kans dat aanvullende medicatie nodig is vanwege de therapie**	**0%<br>(0 van elke 100)**	**10%<br>(10 van elke 100)**
**Behandelfrequentie**	**Twee tot vierwekelijks een infuus of dagelijks een pil, langdurig (jaren)**	**Eenmalig**

Op basis van de informatie in bovenstaande tabel, welke behandeling heeft dan uw voorkeur?

- ◯ Uw huidige therapie
- ◯ Gentherapie

Volgende

Bekijk de informatie in onderstaande tabel.

Bij uw huidige therapie is het gebruik van aanvullende medicatie nodig bij 0% (0 van de 100) van de mensen. Stel dat bij gentherapie bij 50% (50 van de 100) van de mensen aanvullende medicatie nodig zou zijn.

Let op: u kunt met uw muis over de kenmerken aan de linkerkant van de tabel gaan om de uitleg hiervan opnieuw te lezen.

	Uw huidige therapie	Gentherapie
**Werkzaamheid**	**Even goed als gentherapie**	**Even goed als uw huidige therapie**
**Kans op milde bijwerkingen**	**15% (15 van elke 100)**	**60% (60 van elke 100)**
**Kans op ernstige bijwerkingen**	**1% (1 van elke 100)**	**20% (20 van elke 100)**
**Kans dat aanvullende medicatie nodig is vanwege de therapie**	**0% (0 van elke 100)**	**50% (50 van elke 100)**
**Behandelfrequentie**	**Twee tot vierwekelijks een infuus of dagelijks een pil, langdurig (jaren)**	**Eenmalig**

Op basis van de informatie in bovenstaande tabel, welke behandeling heeft dan uw voorkeur?

- ◯ Uw huidige therapie
- ◯ Gentherapie

Volgende

Bekijk de informatie in onderstaande tabel.

Bij uw huidige therapie is het gebruik van aanvullende medicatie nodig bij 0% (0 van de 100) van de mensen. Stel dat bij gentherapie bij 30% (30 van de 100) van de mensen aanvullende medicatie nodig zou zijn.

Let op: u kunt met uw muis over de kenmerken aan de linkerkant van de tabel gaan om de uitleg hiervan opnieuw te lezen.

	Uw huidige therapie	Gentherapie
**Werkzaamheid**	**Even goed als gentherapie**	**Even goed als uw huidige therapie**
**Kans op milde bijwerkingen**	**15% (15 van elke 100)**	**60% (60 van elke 100)**
**Kans op ernstige bijwerkingen**	**1% (1 van elke 100)**	**20% (20 van elke 100)**
**Kans dat aanvullende medicatie nodig is vanwege de therapie**	**0% (0 van elke 100)**	**30% (30 van elke 100)**
**Behandelfrequentie**	**Twee tot vierwekelijks een infuus of dagelijks een pil, langdurig (jaren)**	**Eenmalig**

Op basis van de informatie in bovenstaande tabel, welke behandeling heeft dan uw voorkeur?

- Uw huidige therapie
- Gentherapie

Volgende

U heeft net aangegeven dat u voor uw huidige therapie zou kiezen als de kans op aanvullende medicatie bij gentherapie 5% is.

Wat is de hoogste kans (in %) op aanvullende medicatie waarbij u toch gentherapie zou kiezen? (let op: dit is dus altijd lager dan 5, maar kan ook 0 zijn indien u bijvoorbeeld helemaal geen gentherapie wil)

Volgende

U heeft net aangegeven dat u voor gentherapie zou kiezen als de kans op aanvullende medicatie 50% is.

Wat is de hoogste kans (in %) op aanvullende medicatie waarbij u nog gentherapie zou kiezen? (let op: dit is dus altijd hoger dan 50, maar kan ook 100 zijn indien u altijd voor gentherapie zou kiezen)

Volgende

Gebruikt(e) u op dit moment of in het verleden een of meerdere aanvullende medicijnen die door uw therapie nodig zijn?

- ◯ Ja
- ◯ Nee

Volgende

Deel 4: onzekerheidsvraag

Bij de keuze om wel of niet mee te doen aan een studie naar een nieuw geneesmiddel kan ook de onzekerheid over werkzaamheid en bijwerkingen meespelen. Er moet dan gekozen worden tussen therapie waarvan de werkzaamheid (gedeeltelijk) bekend is (enzymtherapie of substraatremmingstherapie) en een nieuwe therapie in studieverband met meer onzekerheden (gentherapie). Deze vraag gaat over die onzekerheid.

We gaan er in dit scenario van uit dat gentherapie – in ieder geval op korte termijn (tot ca. 2 jaar) - even goed werkt als uw huidige therapie.

uw huidige is voldoende veilig bevonden om op recept voorgeschreven te mogen worden. Voor gentherapie is dat nog niet het geval, dus het wordt alleen nog in studieverband gegeven. Of het veilig en werkzaam is moet nog worden vastgesteld.

Voor de ziekte van Gaucher kan pas na jaren worden geconcludeerd of een nieuwe therapie goed werkt omdat de ziekte zich langzaam ontwikkelt.

Volgende

Bekijk de informatie in onderstaande tabel.

Uw huidige therapie is voldoende veilig bevonden om op recept voorgeschreven te mogen worden. Stel dat voor gentherapie de kans dat dit veilig en werkzaam blijkt 25% is.

Let op: u kunt met uw muis over de kenmerken aan de linkerkant van de tabel gaan om de uitleg hiervan opnieuw te lezen.

	Uw huidige therapie	Gentherapie
**Werkzaamheid op korte termijn (ca. 2 jaar)**	Even goed als gentherapie	Even goed als uw huidige therapie
**Kans dat het middel op langere termijn als veilig en effectief wordt beschouwd**	100%	25%
**Kans op milde bijwerkingen**	15%<br>(15 van elke 100)	60%<br>(60 van elke 100)
**Kans op ernstige bijwerkingen**	1%<br>(1 van elke 100)	20%<br>(20 van elke 100)
**Kans dat aanvullende medicatie nodig is vanwege de therapie**	0%<br>(0 van elke 100)	15%<br>(15 van elke 100)
**Behandelfrequentie**	Twee tot vierwekelijks een infuus of dagelijks een pil, langdurig (jaren)	Eenmalig

Op basis van de informatie in bovenstaande tabel, welke behandeling heeft dan uw voorkeur?

- ◯ Uw huidige therapie
- ◯ Gentherapie

Volgende

Bekijk de informatie in onderstaande tabel.

Uw huidige therapie is voldoende veilig bevonden om op recept voorgeschreven te mogen worden. Stel dat voor gentherapie de kans dat dit veilig en werkzaam blijkt 5% is.

Let op: u kunt met uw muis over de kenmerken aan de linkerkant van de tabel gaan om de uitleg hiervan opnieuw te lezen.

	Uw huidige therapie	Gentherapie
**Werkzaamheid op korte termijn (ca. 2 jaar)**	**Even goed als gentherapie**	**Even goed als uw huidige therapie**
**Kans dat het middel op langere termijn als veilig en effectief wordt beschouwd**	**100%**	**5%**
**Kans op milde bijwerkingen**	**15% (15 van elke 100)**	**60% (60 van elke 100)**
**Kans op ernstige bijwerkingen**	**1% (1 van elke 100)**	**20% (20 van elke 100)**
**Kans dat aanvullende medicatie nodig is vanwege de therapie**	**0% (0 van elke 100)**	**15% (15 van elke 100)**
**Behandelfrequentie**	**Twee tot vierwekelijks een infuus of dagelijks een pil, langdurig (jaren)**	**Eenmalig**

Op basis van de informatie in bovenstaande tabel, welke behandeling heeft dan uw voorkeur?

- ◯ Uw huidige therapie
- ◯ Gentherapie

Volgende

Bekijk de informatie in onderstaande tabel.

Uw huidige therapie is voldoende veilig bevonden om op recept voorgeschreven te mogen worden. Stel dat voor gentherapie de kans dat dit veilig en werkzaam blijkt 10% is.

Let op: u kunt met uw muis over de kenmerken aan de linkerkant van de tabel gaan om de uitleg hiervan opnieuw te lezen.

	Uw huidige therapie	Gentherapie
**Werkzaamheid op korte termijn (ca. 2 jaar))**	Even goed als gentherapie	Even goed als uw huidige therapie
**Kans dat het middel op langere termijn als veilig en effectief wordt beschouwd**	100%	10%
**Kans op milde bijwerkingen**	15%<br>(15 van elke 100)	60%<br>(60 van elke 100)
**Kans op ernstige bijwerkingen**	1%<br>(1 van elke 100)	20%<br>(20 van elke 100)
**Kans dat aanvullende medicatie nodig is vanwege de therapie**	0%<br>(0 van elke 100)	15%<br>(15 van elke 100)
**Behandelfrequentie**	Twee tot vierwekelijks een infuus of dagelijks een pil, langdurig (jaren)	Eenmalig

Op basis van de informatie in bovenstaande tabel, welke behandeling heeft dan uw voorkeur?

- ◯ Uw huidige therapie
- ◯ Gentherapie

Volgende

Bekijk de informatie in onderstaande tabel.

Uw huidige therapie is voldoende veilig bevonden om op recept voorgeschreven te mogen worden. Stel dat voor gentherapie de kans dat dit veilig en werkzaam blijkt 50% is.

Let op: u kunt met uw muis over de kenmerken aan de linkerkant van de tabel gaan om de uitleg hiervan opnieuw te lezen.

	**Uw huidige therapie**	**Gentherapie**
**Werkzaamheid op korte termijn (ca. 2 jaar))**	**Even goed als gentherapie**	**Even goed als uw huidige therapie**
**Kans dat het middel op langere termijn als veilig en effectief wordt geschouwd**	**100%**	**50%**
**Kans op milde bijwerkingen**	**15%**<br>**(15 van elke 100)**	**60%**<br>**(60 van elke 100)**
**Kans op ernstige bijwerkingen**	**1%**<br>**(1 van elke 100)**	**20%**<br>**(20 van elke 100)**
**Kans dat aanvullende medicatie nodig is vanwege de therapie**	**0%**<br>**(0 van elke 100)**	**15%**<br>**(15 van elke 100)**
**Behandelfrequentie**	**Twee tot vierwekelijks een infuus of dagelijks een pil, langdurig (jaren)**	**Eenmalig**

Op basis van de informatie in bovenstaande tabel, welke behandeling heeft dan uw voorkeur?

- ◯ Uw huidige therapie
- ◯ Gentherapie

Volgende

Bekijk de informatie in onderstaande tabel.

Uw huidige therapie is voldoende veilig bevonden om op recept voorgeschreven te mogen worden. Stel dat voor gentherapie de kans dat dit veilig en werkzaam blijkt 30% is.

Let op: u kunt met uw muis over de kenmerken aan de linkerkant van de tabel gaan om de uitleg hiervan opnieuw te lezen.

	**Uw huidige therapie**	**Gentherapie**
**Werkzaamheid op korte termijn (ca. 2 jaar))**	**Even goed als gentherapie**	**Even goed als uw huidige therapie**
**Kans dat het middel op langere termijn als veilig en effectief wordt beschouwd**	**100%**	**30%**
**Kans op milde bijwerkingen**	**15%<br>(15 van elke 100)**	**60%<br>(60 van elke 100)**
**Kans op ernstige bijwerkingen**	**1%<br>(1 van elke 100)**	**20%<br>(20 van elke 100)**
**Kans dat aanvullende medicatie nodig is vanwege de therapie**	**0%<br>(0 van elke 100)**	**15%<br>(15 van elke 100)**
**Behandelfrequentie**	**Twee tot vierwekelijks een infuus of dagelijks een pil, langdurig (jaren)**	**Eenmalig**

Op basis van de informatie in bovenstaande tabel, welke behandeling heeft dan uw voorkeur?

- ○ Uw huidige therapie
- ○ Gentherapie

Volgende

U heeft net aangegeven dat u voor gentherapie zou kiezen als de kans dat gentherapie in de komende jaren als veilig en effectief wordt beschouwd 5% is.

Wat is de minimale kans (in %) dat het middel in de komende jaren als veilig en effectief wordt beschouwd waarbij u nog voor gentherapie zou kiezen? (let op: dit is dus altijd lager dan 5, maar kan ook 0 zijn indien u altijd gentherapie zou kiezen)

Volgende

U heeft net aangegeven dat u voor uw huidige therapie zou kiezen als de kans dat gentherapie in de komende jaren als veilig en effectief wordt beschouwd 50% is.

Wat is de minimale kans (in %) dat het middel in de komende jaren als veilig en effectief wordt beschouwd waarbij u toch voor gentherapie zou kiezen? (let op: dit is dus altijd hoger dan 50, maar kan ook 100 zijn indien u nooit voor gentherapie zou kiezen)

Volgende

Deel 5: Stel gentherapie werkt beter

We gaan er in dit scenario van uit dat gentherapie **een beter effect** heeft dan eerder. Of gentherapie veilig en werkzaam is, moet uitgezocht worden in studieverband.

Volgende

Bekijk de informatie in onderstaande tabel.

Uw huidige therapie is voldoende veilig bevonden om op recept voorgeschreven te mogen worden. Stel dat voor gentherapie de kans dat dit veilig en werkzaam blijkt 25% is.

Let op: u kunt met uw muis over de kenmerken aan de rechterkant van de tabel gaan om de uitleg hiervan opnieuw te lezen.

	Uw huidige therapie	Gentherapie
Werkzaamheid op korte termijn (ca. 2 jaar)	Minder goed dan gentherapie	Beter dan uw huidige therapie
Kans dat het middel op langere termijn als veilig en effectief wordt beschouwd	100%	25%
Kans op milde bijwerkingen	15% (15 van elke 100)	60% (60 van elke 100)
Kans op ernstige bijwerkingen	1% (1 van elke 100)	20% (20 van elke 100)
Kans dat aanvullende medicatie nodig is vanwege de therapie	0% (0 van elke 100)	15% (15 van elke 100)
Behandelfrequentie	Twee tot vierwekelijks een infuus of dagelijks een pil, langdurig (jaren)	Eenmalig

Op basis van de informatie in bovenstaande tabel, welke behandeling heeft dan uw voorkeur?

- ○ Uw huidige therapie
- ○ Gentherapie

Volgende

Bekijk de informatie in onderstaande tabel.

Uw huidige therapie is voldoende veilig bevonden om op recept voorgeschreven te mogen worden. Stel dat voor gentherapie de kans dat dit veilig en werkzaam blijkt 5% is.

Let op: u kunt met uw muis over de kenmerken aan de linkerkant van de tabel gaan om de uitleg hiervan opnieuw te lezen.

	**Uw huidige therapie**	**Gentherapie**
**Werkzaamheid op korte termijn (ca. 2 jaar)**	**Minder goed dan gentherapie**	**Beter dan uw huidige therapie**
**Kans dat het middel op langere termijn als veilig en effectief wordt beschouwd**	**100%**	**5%**
**Kans op milde bijwerkingen**	**15% (15 van elke 100)**	**60% (60 van elke 100)**
**Kans op ernstige bijwerkingen**	**1% (1 van elke 100)**	**20% (20 van elke 100)**
**Kans dat aanvullende medicatie nodig is vanwege de therapie**	**0% (0 van elke 100)**	**15% (15 van elke 100)**
**Behandelfrequentie**	**Twee tot vierwekelijks een infuus of dagelijks een pil, langdurig (jaren)**	**Eenmalig**

Op basis van de informatie in bovenstaande tabel, welke behandeling heeft dan uw voorkeur?

- ○ Uw huidige therapie
- ○ Gentherapie

Volgende

Bekijk de informatie in onderstaande tabel.

Uw huidige therapie is voldoende veilig bevonden om op recept voorgeschreven te mogen worden. Stel dat voor gentherapie de kans dat dit veilig en werkzaam blijkt 10% is.

Let op: u kunt met uw muis over de kenmerken aan de linkerkant van de tabel gaan om de uitleg hiervan opnieuw te lezen.

	**Uw huidige therapie**	**Gentherapie**
**Werkzaamheid op korte termijn (ca. 2 jaar)**	**Minder goed dan gentherapie**	**Beter dan uw huidige therapie**
**Kans dat het middel op langere termijn als veilig en effectief wordt beschouwd**	**100%**	**10%**
**Kans op milde bijwerkingen**	**15% (15 van elke 100)**	**60% (60 van elke 100)**
**Kans op ernstige bijwerkingen**	**1% (1 van elke 100)**	**20% (20 van elke 100)**
**Kans dat aanvullende medicatie nodig is vanwege de therapie**	**0% (0 van elke 100)**	**15% (15 van elke 100)**
**Behandelfrequentie**	**Twee tot vierwekelijks een infuus of dagelijks een pil, langdurig (jaren)**	**Eenmalig**

Op basis van de informatie in bovenstaande tabel, welke behandeling heeft dan uw voorkeur?

- ◯ Uw huidige therapie
- ◯ Gentherapie

Volgende

Bekijk de informatie in onderstaande tabel.

Uw huidige therapie is voldoende veilig bevonden om op recept voorgeschreven te mogen worden. Stel dat voor gentherapie de kans dat dit veilig en werkzaam blijkt 50% is.

Let op: u kunt met uw muis over de kenmerken aan de linkerkant van de tabel gaan om de uitleg hiervan opnieuw te lezen.

	**Uw huidige therapie**	**Gentherapie**
**Werkzaamheid op korte termijn (ca. 2 jaar)**	**Minder goed dan gentherapie**	**Beter dan uw huidige therapie**
**Kans dat het middel op langere termijn als veilig en effectief wordt beschouwd**	**100%**	**50%**
**Kans op milde bijwerkingen**	**15% (15 van elke 100)**	**60% (60 van elke 100)**
**Kans op ernstige bijwerkingen**	**1% (1 van elke 100)**	**20% (20 van elke 100)**
**Kans dat aanvullende medicatie nodig is vanwege de therapie**	**0% (0 van elke 100)**	**15% (15 van elke 100)**
**Behandelfrequentie**	**Twee tot vierwekelijks een infuus of dagelijks een pil, langdurig (jaren)**	**Eenmalig**

Op basis van de informatie in bovenstaande tabel, welke behandeling heeft dan uw voorkeur?

- Uw huidige therapie
- Gentherapie

Volgende

Bekijk de informatie in onderstaande tabel.

Uw huidige therapie is voldoende veilig bevonden om op recept voorgeschreven te mogen worden. Stel dat voor gentherapie de kans dat dit veilig en werkzaam blijkt 30% is.

Let op: u kunt met uw muis over de kenmerken aan de linkerkant van de tabel gaan om de uitleg hiervan opnieuw te lezen.

	Uw huidige therapie	Gentherapie
Werkzaamheid op korte termijn (ca. 2 jaar)	Minder goed dan gentherapie	Beter dan uw huidige therapie
Kans dat het middel op langere termijn als veilig en effectief wordt beschouwd	100%	30%
Kans op milde bijwerkingen	15% (15 van elke 100)	60% (60 van elke 100)
Kans op ernstige bijwerkingen	1% (1 van elke 100)	20% (20 van elke 100)
Kans dat aanvullende medicatie nodig is vanwege de therapie	0% (0 van elke 100)	15% (15 van elke 100)
Behandelfrequentie	Twee tot vierwekelijks een infuus of dagelijks een pil, langdurig (jaren)	Eenmalig

Op basis van de informatie in bovenstaande tabel, welke behandeling heeft dan uw voorkeur?

- ◯ Uw huidige therapie
- ◯ Gentherapie

Volgende

U heeft net aangegeven dat u voor gentherapie zou kiezen als de kans dat gentherapie in de komende jaren als veilig en effectief wordt beschouwd 5% is.

Wat is de minimale kans (in %) dat het middel in de komende jaren als veilig en effectief wordt beschouwd waarbij u nog voor gentherapie zou kiezen? (let op: dit is dus altijd lager dan 5, maar kan ook 0 zijn indien u bijvoorbeeld altijd gentherapie zou kiezen)

Volgende

U heeft net aangegeven dat u voor uw huidige therapie zou kiezen als de kans dat gentherapie in de komende jaren als veilig en effectief wordt beschouwd 50% is.

Wat is de minimale kans (in %) dat het middel in de komende jaren als veilig en effectief wordt beschouwd waarbij u toch voor gentherapie zou kiezen? (let op: dit is dus altijd hoger dan 50, maar kan ook 100 zijn indien u nooit voor gentherapie zou kiezen)

Volgende

Heeft u ooit aan een studie naar een nieuwe (vorm van) therapie voor de ziekte van Gaucher meegewerkt? Studies waar u op dit moment nog aan meewerkt tellen ook mee.

- ◯ Ja
- ◯ Nee

Volgende

Uw mening over de medicijnen die u gebruikt

Als laatste onderdeel van deze vragenlijst willen we u vragen om per vraag het antwoord te kiezen dat het beste uw mening weergeeft over medicatie die u voorgeschreven heeft gekregen of nog krijgt. Het gaat hierbij specifiek om medicatie die u krijgt vanwege de ziekte van Gaucher, zoals enzymtherapie (Cerezyme/imiglucerase® of VPRIV/velaglucerase®) of substraatreductietherapie (Cerdelga/eliglustat®) of bijv. pijnstilling voor botpijn. We vragen u om medicijnen die u eventueel in het kader van andere aandoeningen voorgeschreven krijgt hier niet mee te wegen in uw keuze.

U kunt per vraag een antwoord kiezen.

	Helemaal niet mee eens	Niet mee eens	Geen duidelijke mening	Mee eens	Helemaal mee eens
Op het moment hangt mijn gezondheid af van mijn medicijnen	○	○	○	○	○
Ik maak met zorgen over het feit dat ik medicijnen moet nemen	○	○	○	○	○
Mijn leven zou erg moeilijk zijn zonder medicijnen	○	○	○	○	○
Soms maak ik me zorgen over de effecten die mijn medicijnen op de langere termijn kunnen hebben	○	○	○	○	○
Zonder mijn medicijnen zou ik heel ziek zijn	○	○	○	○	○
Ik ben onvoldoende op de hoogte van wat mijn medicijnen doen	○	○	○	○	○
Mijn toekomstige gezondheid hangt af van mijn medicijnen	○	○	○	○	○
Mijn medicijnen ontwrichten mijn leven	○	○	○	○	○
Soms ben ik bang dat ik te afhankelijk zal worden van mijn medicijnen	○	○	○	○	○
Mijn medicijnen voorkomen dat ik verder achteruit ga	○	○	○	○	○
Deze medicijnen hebben onplezierige bijwerkingen	○	○	○	○	○

Volgende

Afronding vragenlijst & contact

U bent bij het einde van de vragenlijst aangekomen. We willen u van harte bedanken voor uw tijd en het beantwoorden van de vragen. Een samenvatting van onze bevindingen uit dit onderzoek zal na het verwerken van de resultaten met alle deelnemers van dit vragenlijstonderzoek via email worden gedeeld.

Heeft u nog vragen of opmerkingen over de vragenlijst, dit onderzoek of het onderwerp?

Volgende

U gaf aan dat u Migalastat (Galafold©) gebruikt. Om de vragenlijst verder in te vullen en deel te nemen aan dit onderzoek vragen we u vriendelijk contact op te nemen met een van de onderzoekers, Ellie Corazolla (e.m.corazolla@amsterdamumc.nl).

Amsterdam UMC
Universitair Medische Centra

Nogmaals hartelijk dank voor uw medewerking.

Als u aanvullende achtergrond informatie wenst over gentherapie voor stofwisselingsziekten willen we u wijzen op deze uitgebreide informatievideo van patientenvereniging VKS. Klik hier om deze video te bekijken.

PTT survey for women with Fabry disease

Volgende

Doel van dit onderzoek

Uit groepsgesprekken en interviews met patiënten met de ziekte van Fabry is gebleken dat er verschillende factoren zijn die maken of patiënten in de toekomst gentherapie zouden willen ontvangen. De belangrijkste factoren zijn eventuele bijwerkingen van therapie en de te verwachten werkzaamheid (effectiviteit).

Met deze vragenlijst willen wij – samen met FSIGN (de Fabry patiëntenvereniging) en VKS (patiëntenvereniging voor volwassenen en kinderen met een erfelijke stofwisselingsziekte) - onderzoeken welke patiënten wanneer gentherapie zouden willen ontvangen als zij hiervoor (in studieverband) in aanmerking zouden komen.

Om te kunnen onderzoeken in hoeverre deze factoren invloed hebben op de keuzes van patiënten stellen we u vragen over:
- uw huidige situatie
- gentherapie
- geneesmiddelen in het algemeen

Wij vragen u om aan te geven of u onder verschillende omstandigheden zou kiezen voor behandeling met gentherapie of uw huidige therapie. Indien uw huidige behandeling een behandeling in studieverband is dan vragen we u uit te gaan van de situatie voordat u aan de studie meedeed. Meer uitleg hierover vindt u verderop. Voor deze vragenlijst gaan we ervan uit dat u behandeld kunt worden met gentherapie. Of dit in de toekomst daadwerkelijk zo zal zijn is niet bekend.

U kunt de vragenlijst op elk moment sluiten en later op dezelfde plek verdergaan met invullen. Tijdens het invullen van de vragenlijst kunt u niet terug naar vorige vragen. Als u bij het invullen van de vragenlijst hulp wilt of tegen iets aanloopt, sluit de vragenlijst en stuur een mail naar een van de onderzoekers, Ellie Corazolla (e.m.corazolla@amsterdamumc.nl), met de volgende informatie:
- Waar loopt u tegenaan?
- Wilt u via de mail of telefonisch geholpen worden?
- Als u telefonisch contact wenst: Wanneer bent u in de komende dagen bereikbaar? En op welk telefoonnummer?

Amsterdam UMC
Universitair Medische Centra

Volgende

Achtergrondinformatie

Bij de ziekte van Fabry zit er een fout in het erfelijk (genetisch) materiaal (DNA) waardoor een bepaald eiwit (ook wel enzym: alfa-galactosidase) niet of niet goed werkt. Daardoor stapelt er een vetachtige stof in de cellen.

Gentherapie is een vorm van therapie waarbij 'nieuw' erfelijk materiaal in cellen wordt ingebracht. Het doel hiervan is om een stukje DNA toe te voegen zodat er een goed werkend enzym gevormd kan worden. Grofweg zijn er twee soorten gentherapie (zie figuur).

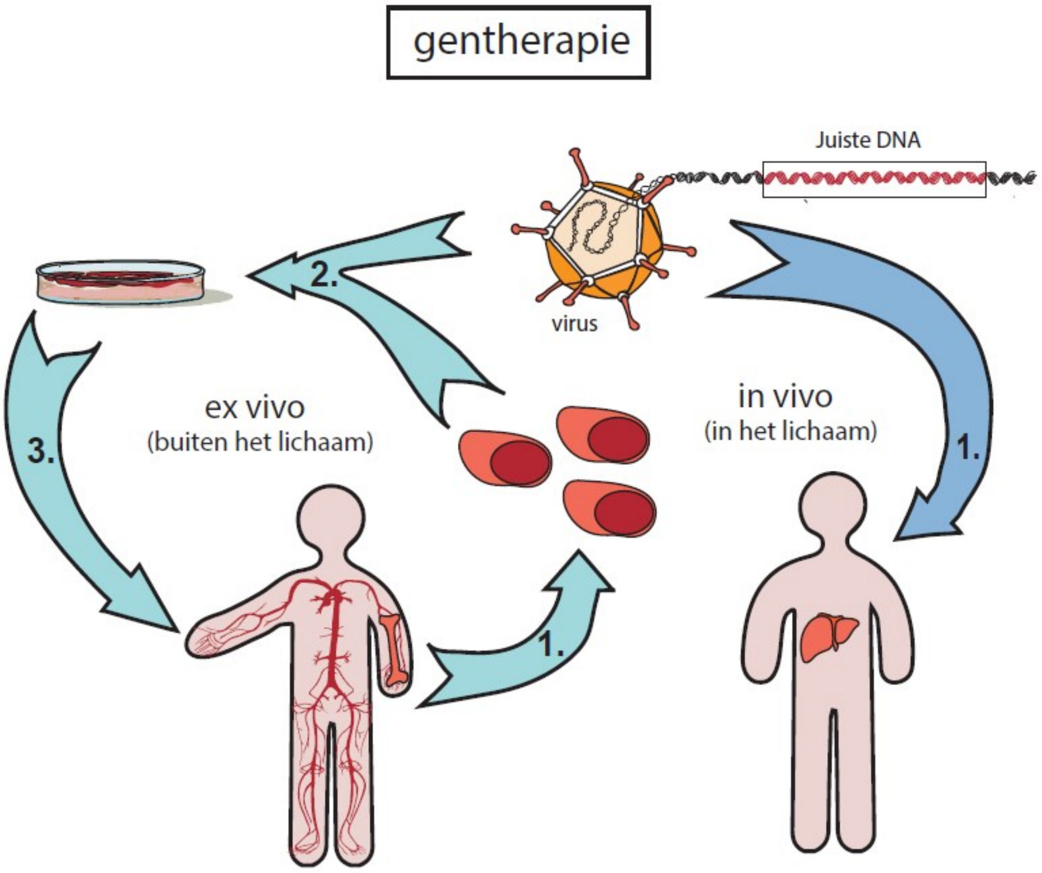

Volgende

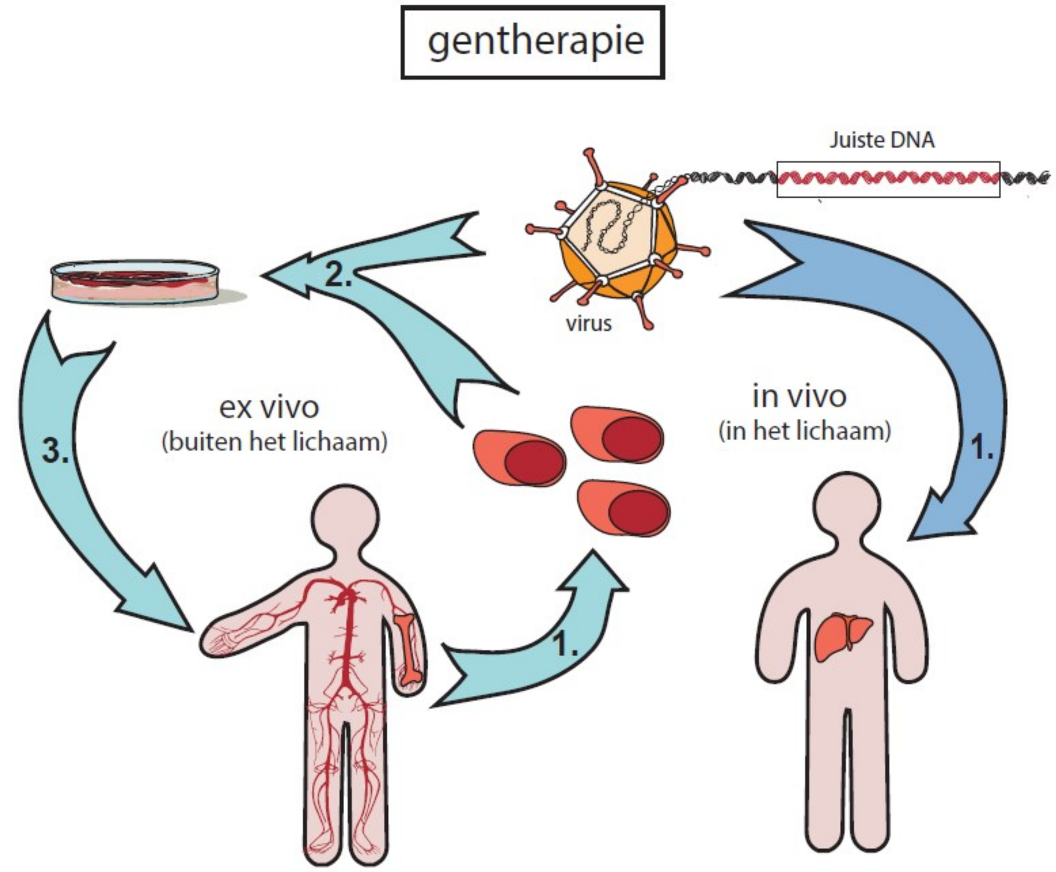

Bij de ene soort ("in vivo") wordt een virusonderdeel waar je niet ziek van wordt in het lichaam gespoten (1.). Dit virusonderdeel heeft het 'nieuwe' DNA bij zich en bouwt dit in bepaalde lichaamscellen in.

- Voordeel van deze vorm van gentherapie: het is een vrij eenvoudige behandeling
- Nadeel van deze vorm van gentherapie: een deel van de mensen hebben het virus al eens heeft 'gehad'. Het lichaam zal het daarom herkennen en opruimen. Óf dit bij mensen inderdaad zo is, moet nog worden onderzocht. Áls dit zo is, dan is het de vraag wat het effect van dit 'opruimen' op de werkzaamheid van de therapie is.

De andere soort ("ex vivo") vindt in meerdere stappen plaats:
1. Er worden beenmergcellen uit het lichaam van patiënten gehaald via een bloedafname.
2. Buiten het lichaam worden de cellen behandeld met gentherapie.
3. Daarna worden de behandelde cellen teruggeplaatst in het lichaam.

- Voordelen van deze vorm van gentherapie: er is controle over de hoeveelheid gentherapie waar de specifieke cellen aan worden blootgesteld. Daarnaast kan het lichaam het DNA mogelijk minder makkelijk 'opruimen' omdat het al in de cellen zit.
- Nadelen van deze vorm van gentherapie: er moet eerst ruimte in het beenmerg komen om de 'nieuwe' cellen te laten uitgroeien voordat de cellen teruggeplaatst kunnen worden. Daarom moet er een milde vorm van chemotherapie worden gegeven. Deze behandeling is kortdurend. Daarnaast is er nog geen zekerheid of deze vorm van gentherapie tot blijvende

enzymproductie leidt.

Op dit moment wordt er onderzoek gedaan naar beide soorten gentherapie voor de ziekte van Fabry. Bij de vragen die we u zullen stellen maken we geen onderscheid tussen de verschillende vormen.

Volgende

Achtergrondinformatie

In welke leeftijdcategorie valt u?

- ◯ 18 jaar of jonger
- ◯ Tussen de 18 - 29 jaar
- ◯ Tussen 30 - 39 jaar
- ◯ Tussen 40 - 49 jaar
- ◯ Tussen 50 - 59 jaar
- ◯ Tussen 60 - 69 jaar
- ◯ Tussen 70 - 79 jaar
- ◯ 80 jaar of ouder

Gebruikt u op dit moment medicijnen voor de ziekte van Fabry (hierbij wordt bedoeld: **enzymtherapie** en/of **chaperone therapie** (Migalastat/Galafold©); het gaat hier **niet** om bloeddrukverlagers, bloedverdunners en/of pijnstillers)?

- ◯ Ja
- ◯ Nee

Volgende

Welk medicijn gebruikt u?

- ◯ Enzymtherapie: Agalsidase beta (Fabrazyme©)
- ◯ Enzymtherapie: Agalsidase alfa (Replagal©)
- ◯ Chaperone therapie: Migalastat (Galafold©)
- ◯ Medicatie in studieverband

Volgende

Welk medicijn voor de ziekte van Fabry kreeg u voordat u met de studie begon?

- ◯ Enzymtherapie: Agalsidase beta (Fabrazyme©)
- ◯ Enzymtherapie: Agalsidase alfa (Replagal©)

Volgende

Heeft u in het verleden enzymtherapie gehad?

- ◯ Ja, ik heb in het verleden wel behandeling met enzymtherapie (Agalsidase beta/Fabrazyme© of Agalsidase alfa/Replagal©) gehad maar dit is gestopt
- ◯ Nee, ik heb nooit behandeling met enzymtherapie (Agalsidase beta/Fabrazyme© of Agalsidase alfa/Replagal©) gehad

Volgende

Keuzetaken

We vragen u straks steeds een keuze te maken uit twee behandelopties; enzymtherapie of gentherapie. De keuzes zullen erg op elkaar lijken, toch zijn er kleine verschillen. Het is belangrijk voor ons dat u de informatie op de volgende pagina's goed leest en alle keuzetaken invult, we zullen hier de verschillende kenmerken van de behandelingen toelichten.

We vragen bij de keuzetaken om uw persoonlijke mening, er zijn dus geen goede of foute antwoorden/keuzes.

Het is belangrijk om te vermelden dat het mogelijk is om eerder gebruikte therapie weer te hervatten als gentherapie niet goed werkt.

Volgende

Keuzetaken

We vragen u straks steeds een keuze te maken uit twee behandelopties; enzymtherapie of gentherapie. De keuzes zullen erg op elkaar lijken, toch zijn er kleine verschillen. Het is belangrijk voor ons dat u de informatie op de volgende pagina's goed leest en alle keuzetaken invult, we zullen hier de verschillende kenmerken van de behandelingen toelichten.

We vragen bij de keuzetaken om uw persoonlijke mening, er zijn dus geen goede of foute antwoorden/keuzes.

Het is belangrijk om te vermelden dat het mogelijk is om eerder gebruikte therapie weer te hervatten als gentherapie niet goed werkt.

Volgende

Keuzetaken

We vragen u straks steeds een keuze te maken uit twee behandelopties; enzymtherapie of gentherapie. De keuzes zullen erg op elkaar lijken, toch zijn er kleine verschillen. Het is belangrijk voor ons dat u de informatie op de volgende pagina's goed leest en alle keuzetaken invult, we zullen hier de verschillende kenmerken van de behandelingen toelichten.

We vragen bij de keuzetaken om uw persoonlijke mening, er zijn dus geen goede of foute antwoorden/keuzes.

Volgende

Keuzetaken

We vragen u straks steeds een keuze te maken uit twee behandelopties; enzymtherapie of gentherapie. De keuzes zullen erg op elkaar lijken, toch zijn er kleine verschillen. Het is belangrijk voor ons dat u de informatie op de volgende pagina's goed leest en alle keuzetaken invult, we zullen hier de verschillende kenmerken van de behandelingen toelichten.

We vragen bij de keuzetaken om uw persoonlijke mening, er zijn dus geen goede of foute antwoorden/keuzes.

Volgende

Uitleg over de werkzaamheid van de behandeling

We gaan ervan uit dat de gentherapie even goed werkt als uw huidige therapie. Gentherapie is een eenmalige behandeling; bij uw huidige behandeling is het noodzakelijk om herhaaldelijke infusies te ondergaan. Het ondergaan van gentherapie kan gepaard gaan met milde en/of ernstige bijwerkingen. Ook bestaat er een kans dat er extra medicatie gebruikt moet worden. Tot slot is er nog onzekerheid over of gentherapie goed blijft werken op de lange termijn. In de vragenlijst stellen we u vragen die steeds betrekking hebben op een van deze aspecten. We vragen u bij elke vraag een keuze te maken tussen gentherapie en uw huidige therapie.

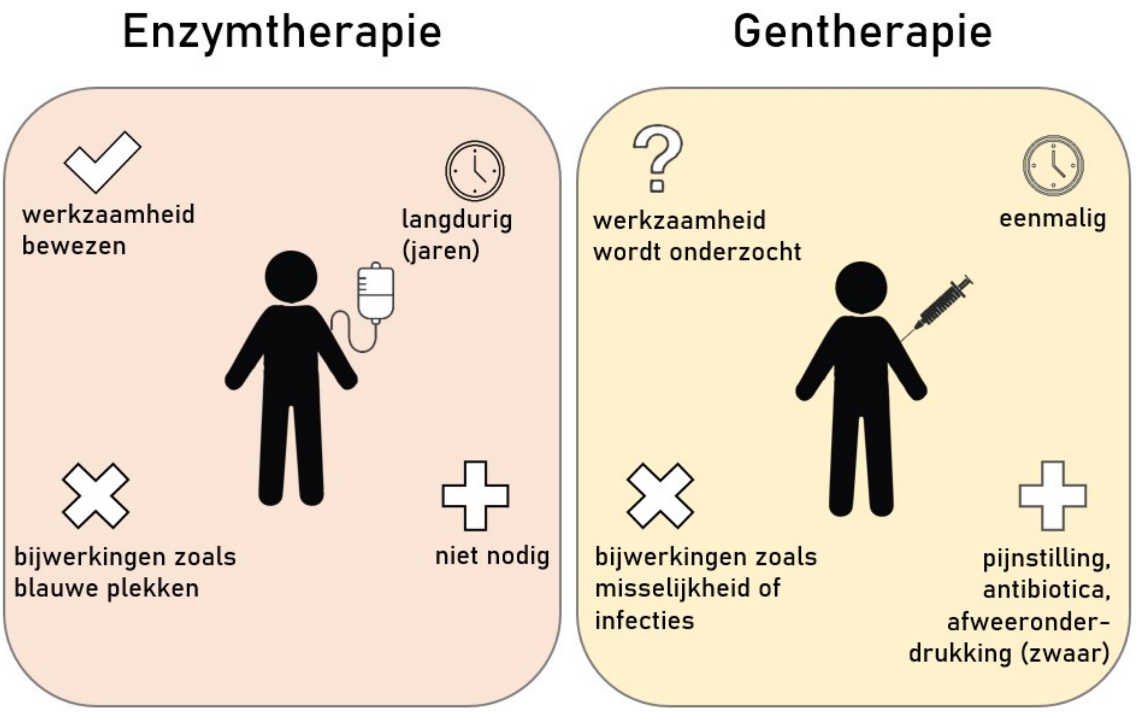

Volgende

Uitleg over de werkzaamheid van de behandeling

U gebruikt op dit moment enzymtherapie in studieverband. We vragen u om voor deze vragenlijst uit te gaan van uw situatie vóórdat u met de studie begon, toen u Agalsidase alfa/Replagal© of Agalsidase beta/Fabrazyme© kreeg.

We gaan ervan uit dat de gentherapie even goed werkt als enzymtherapie. Gentherapie is een eenmalige behandeling; bij enzymtherapie is het noodzakelijk om herhaaldelijke infusies te ondergaan. Het ondergaan van gentherapie kan gepaard gaan met milde en/of ernstige bijwerkingen. Ook bestaat er een kans dat er extra medicatie gebruikt moet worden. Tot slot is er nog onzekerheid over of gentherapie goed blijft werken op de lange termijn. In de vragenlijst stellen we u vragen die steeds betrekking hebben op een van deze aspecten. We vragen u bij elke vraag een keuze te maken tussen gentherapie en de therapie die u in het verleden gebruikte.

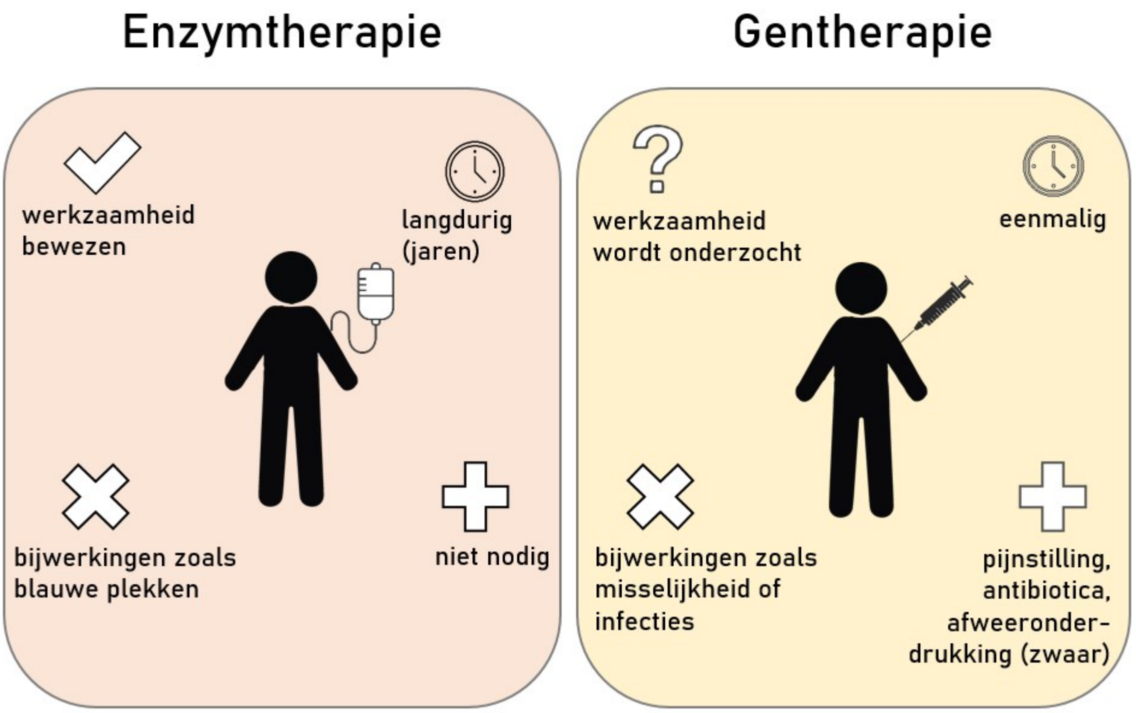

Volgende

Uitleg over de werkzaamheid van de behandeling

U gebruikt op dit moment geen therapie en heeft dat nog nooit eerder gebruikt. We vragen u om er bij het invullen van de vragenlijst van uit te gaan dat u enzymtherapie krijgt. Dat betekent dat u elke twee weken een infuus krijgt met daarin het enzym wat u zelf niet goed aan kunt maken. De eerste infusen krijgt u in het ziekenhuis, daarna kan het thuis met behulp van de thuiszorg. Over het algemeen krijgen vrouwen geen bijwerkingen van de enzymtherapie.

We gaan ervan uit dat de gentherapie even goed werkt als enzymtherapie. Gentherapie is een eenmalige behandeling; bij enzymtherapie is het noodzakelijk om herhaaldelijke infusies te ondergaan. Het ondergaan van gentherapie kan gepaard gaan met milde en/of ernstige bijwerkingen. Ook bestaat er een kans dat er extra medicatie gebruikt moet worden. Tot slot is er nog onzekerheid over of gentherapie goed blijft werken op de lange termijn. In de vragenlijst stellen we u vragen die steeds betrekking hebben op een van deze aspecten. We vragen u bij elke vraag een keuze te maken tussen gentherapie en enzymtherapie.

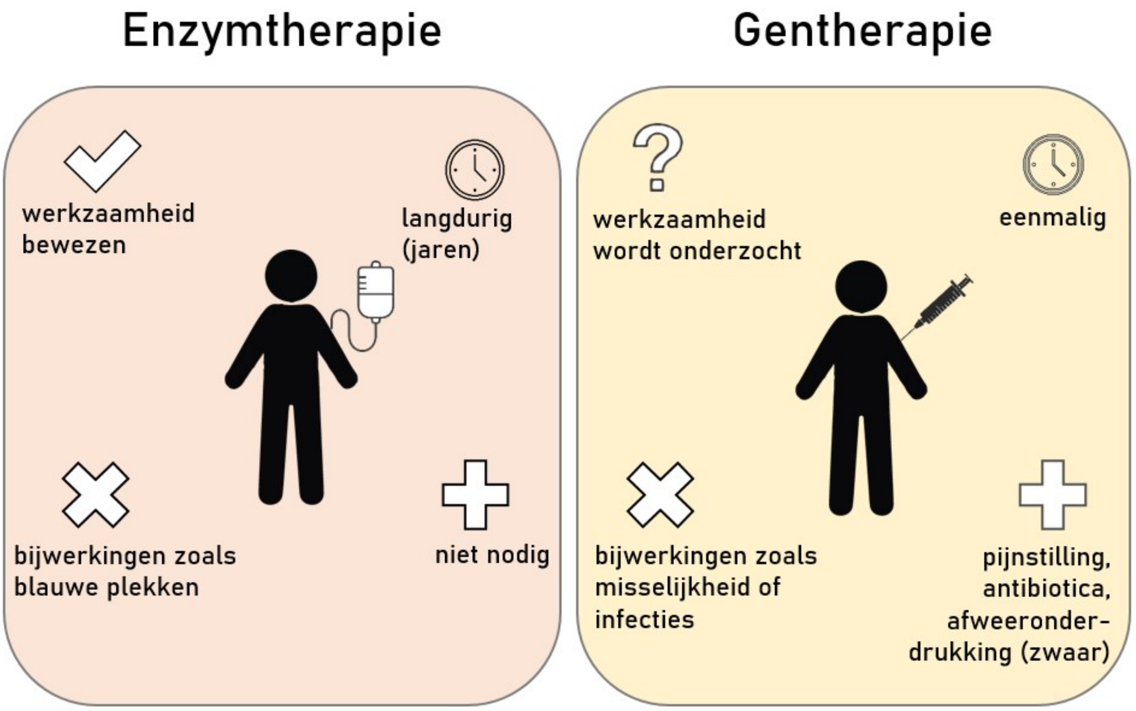

Volgende

Uitleg over de werkzaamheid van de behandeling

U gebruikt op dit moment geen therapie maar heeft dat wel in het verleden gebruikt. We vragen u om voor deze vragenlijst uit te gaan van uw situatie waarin u enzymtherapie (Agalsidase alfa/Replagal© of Agalsidase beta/Fabrazyme©) als tweewekelijks infuus kreeg en de verwachting was dat dat een positief effect had op het ziektebeloop.

We gaan ervan uit dat de gentherapie even goed werkt als enzymtherapie. Dit is het geval wanneer de therapie in een vroeg stadium van de ziekte gegeven wordt. Gentherapie is een eenmalige behandeling; bij enzymtherapie is het noodzakelijk om herhaaldelijke infusies te ondergaan. Het ondergaan van gentherapie kan gepaard gaan met milde en/of ernstige bijwerkingen. Ook bestaat er een kans dat er extra medicatie gebruikt moet worden. Tot slot is er nog onzekerheid over of gentherapie goed blijft werken op de lange termijn. In de vragenlijst stellen we u vragen die steeds betrekking hebben op een van deze aspecten. We vragen u bij elke vraag een keuze te maken tussen gentherapie en de therapie die u in het verleden gebruikte.

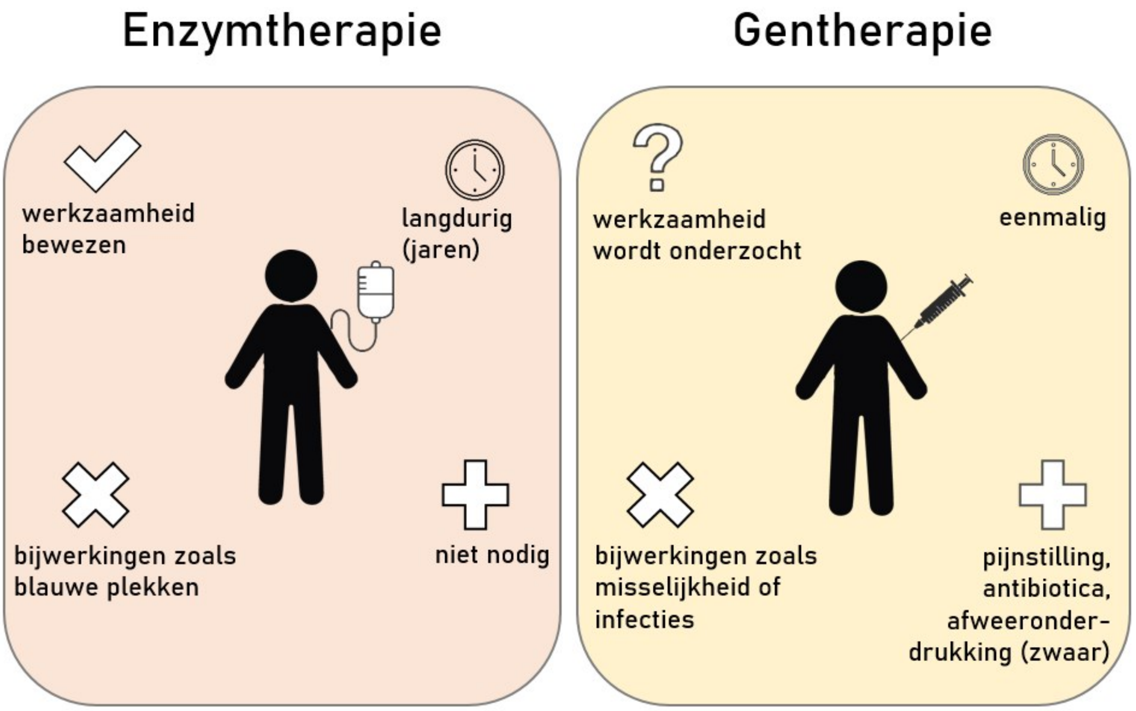

Volgende

Uitleg over mogelijke bijwerkingen van de behandelingen

Milde bijwerkingen: bij het gebruik van enzymtherapie of gentherapie kunnen milde bijwerkingen optreden. Deze bijwerkingen duren kort, zijn zonder blijvende gevolgen en er is geen ziekenhuisopname nodig.

Voor enzymtherapie: reacties op infusies die zich uiten als
- blauwe plekken of bloeduitstortingen

Voor gentherapie:
- het krijgen van een blauwe plek of bloeduitstorting
- tijdelijk algeheel niet lekker zijn (malaise)
- milde griepachtige klachten
- kortdurende misselijkheid of overgeven
- buikpijn of diarree
- ongevaarlijke infecties
- bloedarmoede
- pijnlijke ontstekingen van het mondslijmvlies
- koorts

Ernstige bijwerkingen: bij het gebruik van enzymtherapie of gentherapie kunnen ernstige bijwerkingen optreden. Deze bijwerkingen hebben een ziekenhuisopname tot gevolg.

Voor enzymtherapie komen ernstige bijwerkingen bij vrouwen met de ziekte van Fabry niet voor.

Voor gentherapie: ziekenhuisopnames vanwege bijvoorbeeld
- zeer hoge koorts
- ernstige infecties

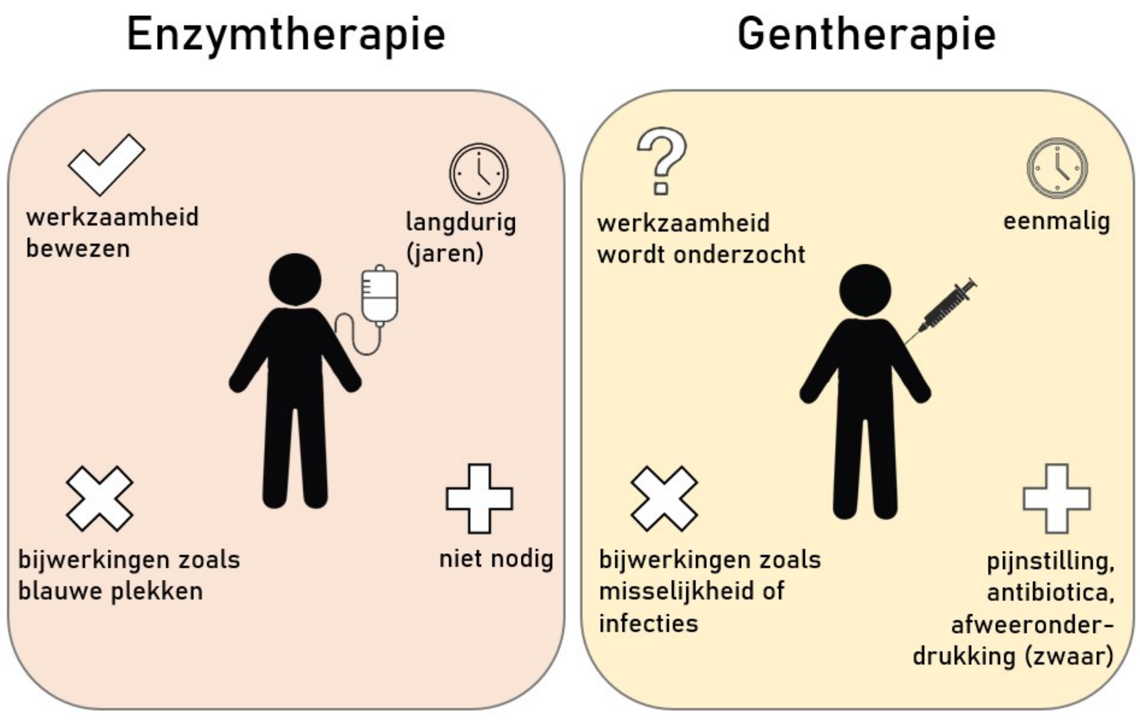

Volgende

Uitleg over aanvullende medicatie en behandelfrequentie van de behandelingen.

Aanvullende medicatie: het kan nodig zijn om de bijwerkingen van enzymtherapie of gentherapie te onderdrukken met medicatie. Dit kan zowel kortdurend als langdurig zijn. Met deze medicatie bedoelen we geen medicijnen die gebruikt worden om eventuele al bestaande problemen door de ziekte te behandelen (bijvoorbeeld een middel dat nodig is vanwege verminderde nierfunctie), maar medicijnen die verschijnselen die de therapie veroorzaakt tegengaan (bijvoorbeeld misselijkheid door de gentherapie).

Voor enzymtherapie: komt aanvullende medicatie vanwege de enzymtherapie bij vrouwen met de ziekte van Fabry niet voor.

Voor gentherapie: Dit kan gaan om een immuunsysteem onderdrukkend middel, zoals prednison. Dat wordt kortdurend (weken tot maanden) gegeven om een afweerreactie van het lichaam tegen het (ongevaarlijke) virusonderdeel tegen te gaan. Daarnaast kan medicatie nodig zijn om bijwerkingen te verhelpen, zoals een antibioticakuur, pijnstilling, of middelen tegen misselijkheid.

Behandelfrequentie: voor enzymtherapie geldt dat u langdurig (jaren) elke twee weken een infuus krijgt terwijl voor gentherapie geldt dat de behandeling eenmalig is.

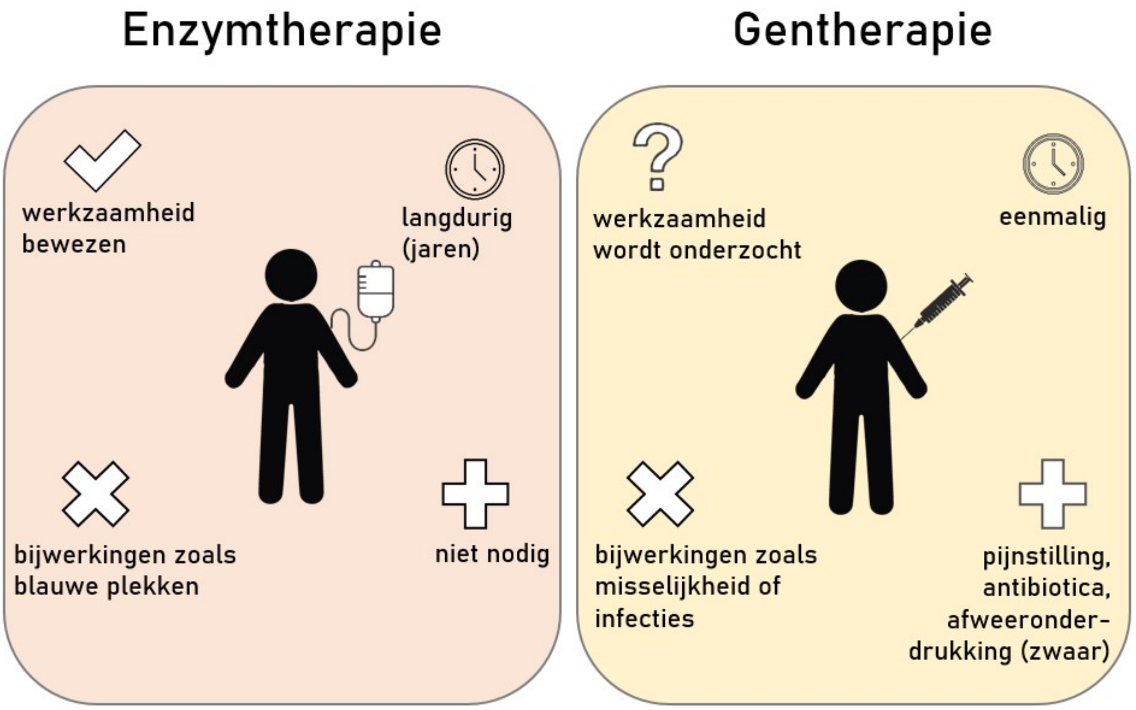

Volgende

Uitleg over waarschijnlijkheid en kansen

Voor verschillende kenmerken van behandelingen wordt straks geproken over 'waarschijnlijkheid'. Hiermee bedoelen we de kans dat de behandeling milde of ernstige bijwerkingen tot gevolg heeft of de kans dat u aanvullende medicatie zou moeten nemen. Er worden verschillende kansen aan u gepresenteerd. Wanneer de waarschijnlijkheid (oftewel kans) op een bijwerking 40% is dan zullen 40 van elke 100 vrouwen die het medicijn nemen bijwerkingen krijgen terwijl 60 van de 100 vrouwen die het medicijn nemen geen bijwerkingen zullen ervaren. Dit ziet er als volgt uit:

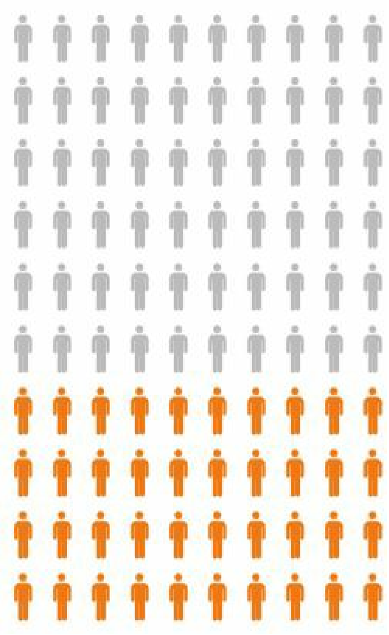

Volgende

Het invullen van de keuzetaken

We vragen u zo meerdere keren een keuze te maken uit twee behandelopties; enzymtherapie of gentherapie.

De behandelingen zullen erg op elkaar lijken, toch zijn er kleine verschillen.

De keuzes zullen eruit zien zoals in het plaatje hieronder. Aan de linkerkant ziet u de eigenschappen van de behandelingen staan. Wanneer u straks de keuzetaken gaat invullen kunt u hier met uw muis op gaat staan, om de uitleg die hierbij hoort nogmaals lezen.

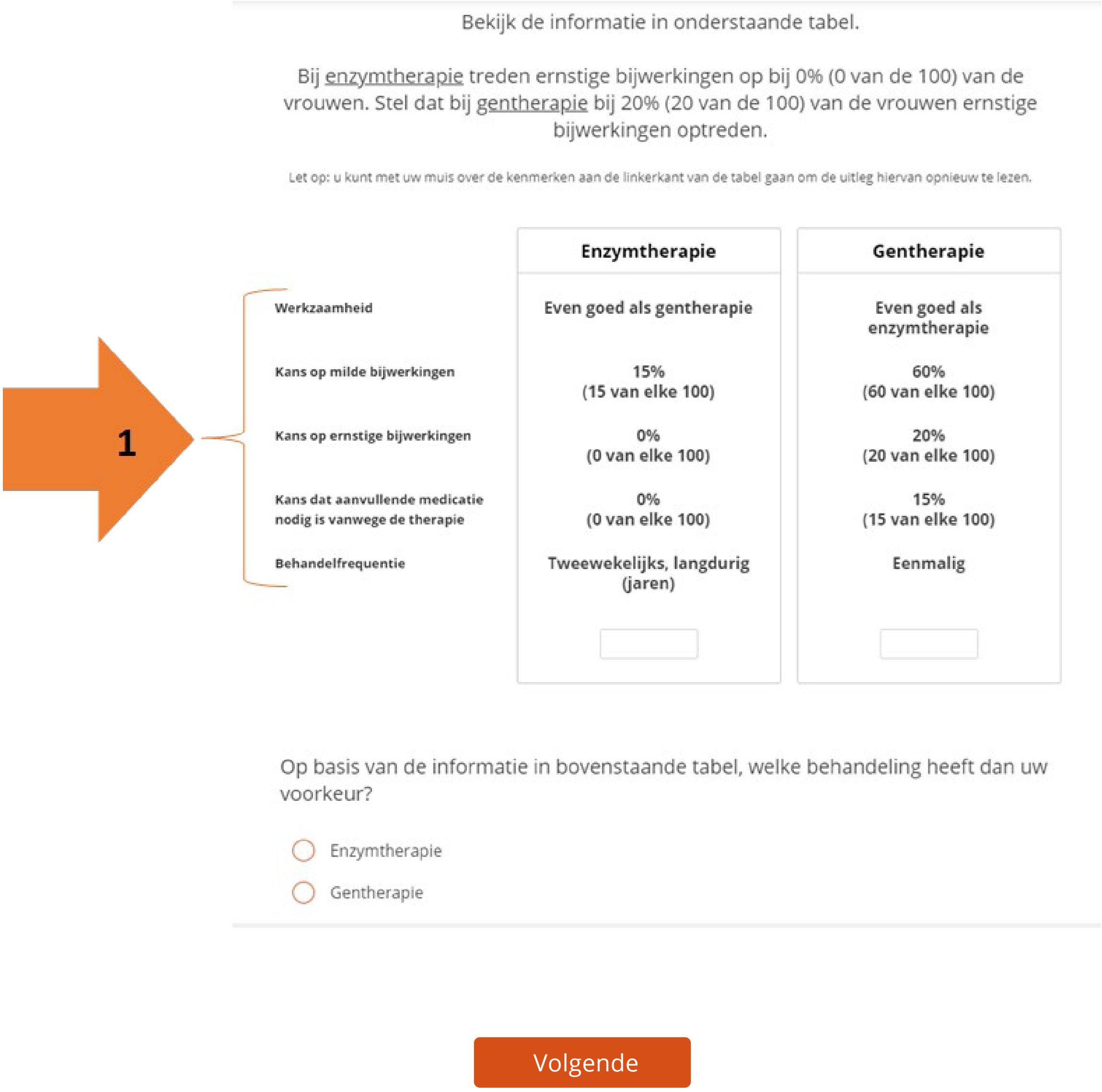
Bekijk de informatie in onderstaande tabel.

Bij enzymtherapie treden ernstige bijwerkingen op bij 0% (0 van de 100) van de vrouwen. Stel dat bij gentherapie bij 20% (20 van de 100) van de vrouwen ernstige bijwerkingen optreden.

Let op: u kunt met uw muis over de kenmerken aan de linkerkant van de tabel gaan om de uitleg hiervan opnieuw te lezen.

	Enzymtherapie	Gentherapie
Werkzaamheid	Even goed als gentherapie	Even goed als enzymtherapie
Kans op milde bijwerkingen	15% (15 van elke 100)	60% (60 van elke 100)
Kans op ernstige bijwerkingen	0% (0 van elke 100)	20% (20 van elke 100)
Kans dat aanvullende medicatie nodig is vanwege de therapie	0% (0 van elke 100)	15% (15 van elke 100)
Behandelfrequentie	Tweewekelijks, langdurig (jaren)	Eenmalig

Op basis van de informatie in bovenstaande tabel, welke behandeling heeft dan uw voorkeur?

- Enzymtherapie
- Gentherapie

Volgende

In het midden van de taak ziet u de twee behandelingen.

Beide behandelingen hebben net iets andere niveaus van hun eigenschappen.

In het onderstaande voorbeeld werken beide behandelingen even goed. U heeft bij de gentherapie meer kans op ernstige bijwerkingen (namelijk 20% in plaats van 0% voor enzymtherapie), maar gentherapie is een eenmalige behandeling terwijl enzymtherapie langdurig (jaren) tweewekelijks moet worden toegediend.

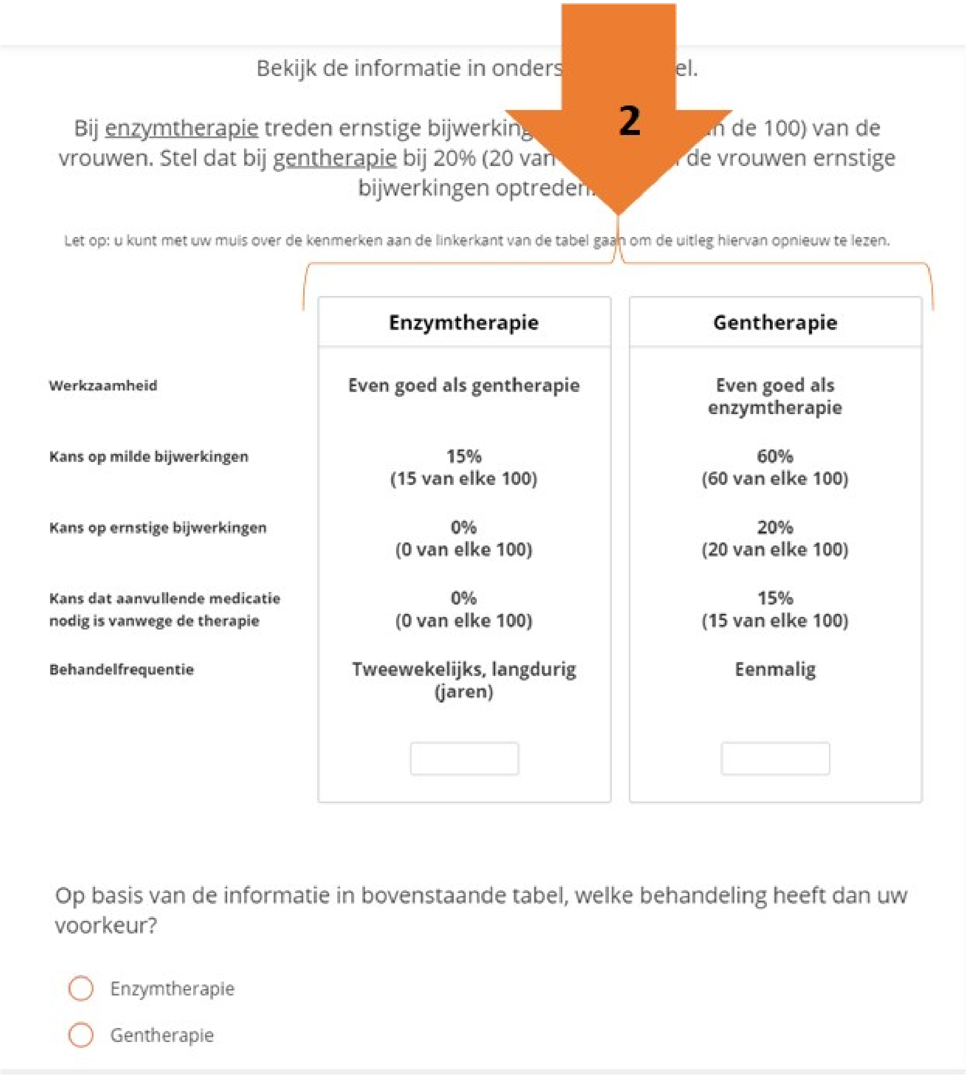
Bekijk de informatie in onders[illegible]el.

Bij enzymtherapie treden ernstige bijwerkin[illegible] n de 100) van de vrouwen. Stel dat bij gentherapie bij 20% (20 van [illegible] de vrouwen ernstige bijwerkingen optreden[illegible]

Let op: u kunt met uw muis over de kenmerken aan de linkerkant van de tabel gaan om de uitleg hiervan opnieuw te lezen.

	Enzymtherapie	Gentherapie
Werkzaamheid	Even goed als gentherapie	Even goed als enzymtherapie
Kans op milde bijwerkingen	15% (15 van elke 100)	60% (60 van elke 100)
Kans op ernstige bijwerkingen	0% (0 van elke 100)	20% (20 van elke 100)
Kans dat aanvullende medicatie nodig is vanwege de therapie	0% (0 van elke 100)	15% (15 van elke 100)
Behandelfrequentie	Tweewekelijks, langdurig (jaren)	Eenmalig

Op basis van de informatie in bovenstaande tabel, welke behandeling heeft dan uw voorkeur?

- Enzymtherapie
- Gentherapie

Volgende

Vervolgens is het aan u om een afweging te maken tussen deze behandelingen en hun eigenschappen en een keuze te maken.

U kunt uw keuze bevestigen door:
1. In de tabel op de therapie van uw voorkeur te klikken
2. In de vraag onder de tabel dezelfde therapie aan te klikken

Het is belangrijk dat u uw keuze in de tabel nogmaals bevestigt in de vraag onder elke tabel zoals in het voorbeeld hieronder waar de patient voor gentherapie heeft gekozen.

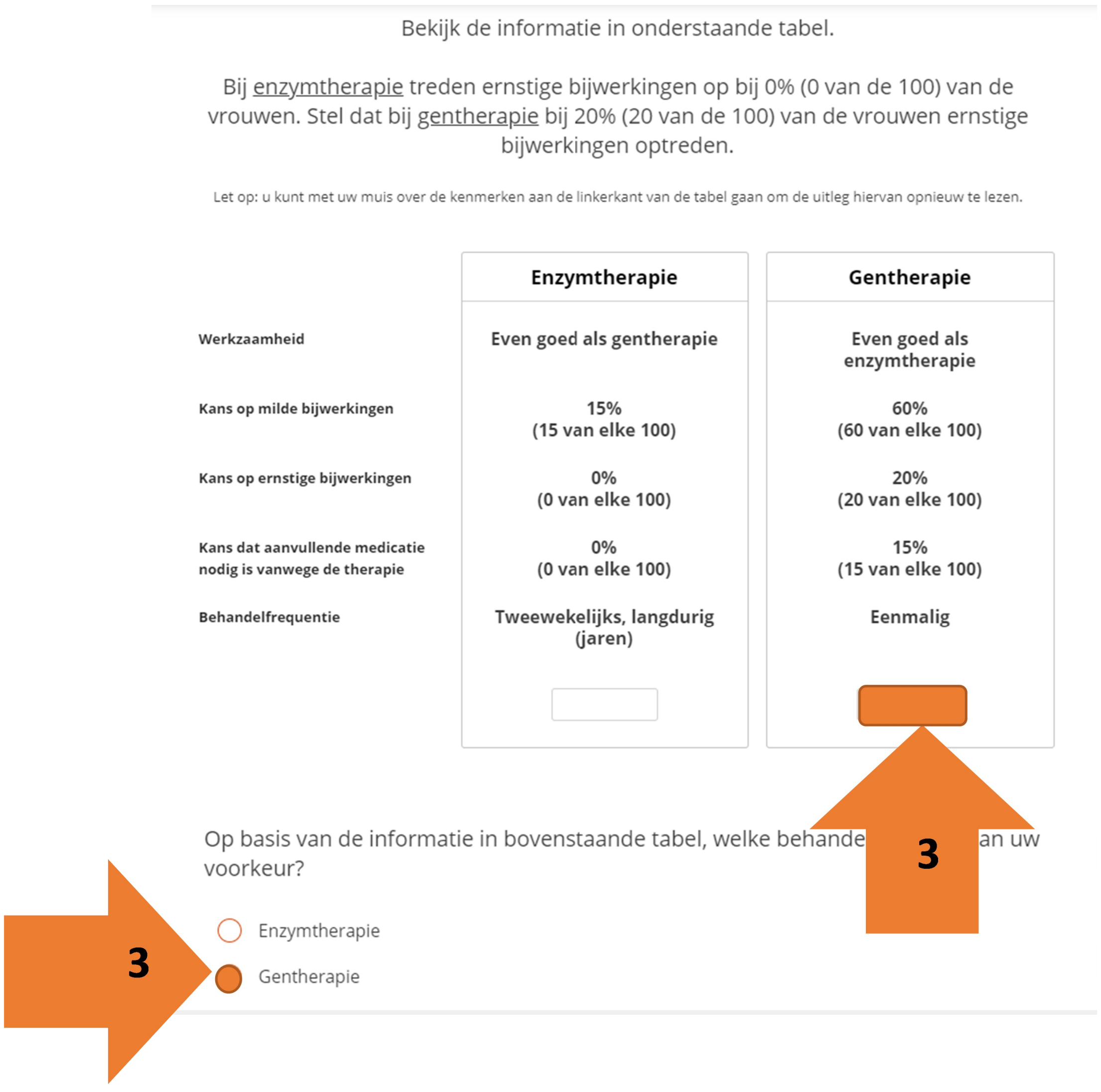

Bekijk de informatie in onderstaande tabel.

Bij enzymtherapie treden ernstige bijwerkingen op bij 0% (0 van de 100) van de vrouwen. Stel dat bij gentherapie bij 20% (20 van de 100) van de vrouwen ernstige bijwerkingen optreden.

Let op: u kunt met uw muis over de kenmerken aan de linkerkant van de tabel gaan om de uitleg hiervan opnieuw te lezen.

	Enzymtherapie	Gentherapie
Werkzaamheid	Even goed als gentherapie	Even goed als enzymtherapie
Kans op milde bijwerkingen	15% (15 van elke 100)	60% (60 van elke 100)
Kans op ernstige bijwerkingen	0% (0 van elke 100)	20% (20 van elke 100)
Kans dat aanvullende medicatie nodig is vanwege de therapie	0% (0 van elke 100)	15% (15 van elke 100)
Behandelfrequentie	Tweewekelijks, langdurig (jaren)	Eenmalig

Op basis van de informatie in bovenstaande tabel, welke behande [illegible] an uw voorkeur?

- Enzymtherapie
- Gentherapie

Volgende

Deel 1: Milde bijwerkingen

Bij dit onderdeel van de vragenlijst gaat het om milde bijwerkingen die kunnen optreden bij het gebruik van enzymtherapie of gentherapie. Deze bijwerkingen duren kort, zijn zonder blijvende gevolgen en er is geen ziekenhuisopname nodig.

Volgende

Bekijk de informatie in onderstaande tabel.

Bij enzymtherapie treden milde bijwerkingen op bij 15% (15 van de 100) van de vrouwen. Stel dat ook bij gentherapie bij 60% (60 van de 100) van de vrouwen milde bijwerkingen optreden.

Let op: u kunt met uw muis over de kenmerken aan de linkerkant van de tabel gaan om de uitleg hiervan opnieuw te lezen.

	**Enzymtherapie**	**Gentherapie**
**Werkzaamheid**	**Even goed als gentherapie**	**Even goed als enzymtherapie**
**Kans op milde bijwerkingen**	**15% (15 van elke 100)**	**60% (60 van elke 100)**
**Kans op ernstige bijwerkingen**	**0% (0 van elke 100)**	**20% (20 van elke 100)**
**Kans dat aanvullende medicatie nodig is vanwege de therapie**	**0% (0 van elke 100)**	**15% (15 van elke 100)**
**Behandelfrequentie**	**Tweewekelijks, langdurig (jaren)**	**Eenmalig**

Op basis van de informatie in bovenstaande tabel, welke behandeling heeft dan uw voorkeur?

- ○ Enzymtherapie
- ○ Gentherapie

Volgende

Bekijk de informatie in onderstaande tabel.

Bij enzymtherapie treden milde bijwerkingen op bij 15% (15 van de 100) van de vrouwen. Stel dat bij gentherapie bij 40% (40 van de 100) van de vrouwen milde bijwerkingen optreden.

Let op: u kunt met uw muis over de kenmerken aan de linkerkant van de tabel gaan om de uitleg hiervan opnieuw te lezen.

	**Enzymtherapie**	**Gentherapie**
**Werkzaamheid**	**Even goed als gentherapie**	**Even goed als enzymtherapie**
**Kans op milde bijwerkingen**	**15% (15 van elke 100)**	**40% (40 van elke 100)**
**Kans op ernstige bijwerkingen**	**0% (0 van elke 100)**	**20% (20 van elke 100)**
**Kans dat aanvullende medicatie nodig is vanwege de therapie**	**0% (0 van elke 100)**	**15% (15 van elke 100)**
**Behandelfrequentie**	**Tweewekelijks, langdurig (jaren)**	**Eenmalig**

Op basis van de informatie in bovenstaande tabel, welke behandeling heeft dan uw voorkeur?

- Enzymtherapie
- Gentherapie

Volgende

Bekijk de informatie in onderstaande tabel.

Bij enzymtherapie treden milde bijwerkingen op bij 15% (15 van de 100) van de vrouwen. Stel dat bij gentherapie bij 50% (50 van de 100) van de vrouwen milde bijwerkingen optreden.

Let op: u kunt met uw muis over de kenmerken aan de linkerkant van de tabel gaan om de uitleg hiervan opnieuw te lezen.

	**Enzymtherapie**	**Gentherapie**
**Werkzaamheid**	**Even goed als gentherapie**	**Even goed als enzymtherapie**
**Kans op milde bijwerkingen**	**15% (15 van elke 100)**	**50% (50 van elke 100)**
**Kans op ernstige bijwerkingen**	**0% (0 van elke 100)**	**20% (20 van elke 100)**
**Kans dat aanvullende medicatie nodig is vanwege de therapie**	**0% (0 van elke 100)**	**15% (15 van elke 100)**
**Behandelfrequentie**	**Tweewekelijks, langdurig (jaren)**	**Eenmalig**

Op basis van de informatie in bovenstaande tabel, welke behandeling heeft dan uw voorkeur?

- ◯ Enzymtherapie
- ◯ Gentherapie

Volgende

Bekijk de informatie in onderstaande tabel.

Bij enzymtherapie treden milde bijwerkingen op bij 15% (15 van de 100) van de vrouwen. Stel dat bij gentherapie bij 80% (80 van de 100) van de vrouwen milde bijwerkingen optreden.

Let op: u kunt met uw muis over de kenmerken aan de linkerkant van de tabel gaan om de uitleg hiervan opnieuw te lezen.

	**Enzymtherapie**	**Gentherapie**
**Werkzaamheid**	**Even goed als gentherapie**	**Even goed als enzymtherapie**
**Kans op milde bijwerkingen**	**15% (15 van elke 100)**	**80% (80 van elke 100)**
**Kans op ernstige bijwerkingen**	**0% (0 van elke 100)**	**20% (20 van elke 100)**
**Kans dat aanvullende medicatie nodig is vanwege de therapie**	**0% (0 van elke 100)**	**15% (15 van elke 100)**
**Behandelfrequentie**	**Tweewekelijks, langdurig (jaren)**	**Eenmalig**

Op basis van de informatie in bovenstaande tabel, welke behandeling heeft dan uw voorkeur?

- ◯ Enzymtherapie
- ◯ Gentherapie

Volgende

Bekijk de informatie in onderstaande tabel.

Bij enzymtherapie treden milde bijwerkingen op bij 15% (15 van de 100) van de vrouwen. Stel dat bij gentherapie bij 70% (70 van de 100) van de vrouwen milde bijwerkingen optreden.

Let op: u kunt met uw muis over de kenmerken aan de linkerkant van de tabel gaan om de uitleg hiervan opnieuw te lezen.

	**Enzymtherapie**	**Gentherapie**
**Werkzaamheid**	**Even goed als gentherapie**	**Even goed als enzymtherapie**
**Kans op milde bijwerkingen**	**15% (15 van elke 100)**	**70% (70 van elke 100)**
**Kans op ernstige bijwerkingen**	**0% (0 van elke 100)**	**20% (20 van elke 100)**
**Kans dat aanvullende medicatie nodig is vanwege de therapie**	**0% (0 van elke 100)**	**15% (15 van elke 100)**
**Behandelfrequentie**	**Tweewekelijks, langdurig (jaren)**	**Eenmalig**

Op basis van de informatie in bovenstaande tabel, welke behandeling heeft dan uw voorkeur?

- ○ Enzymtherapie
- ○ Gentherapie

Volgende

U heeft net aangegeven dat u voor enzymtherapie zou kiezen als de kans op milde bijwerkingen bij gentherapie 40% is.

Wat is de hoogste kans (in %) op milde bijwerkingen waarbij u toch gentherapie zou kiezen? (let op: dit is dus altijd lager dan 40, maar kan ook 0 zijn indien u bijvoorbeeld helemaal geen gentherapie wil)

Volgende

U heeft net aangegeven dat u voor gentherapie zou kiezen als de kans op milde bijwerkingen 80% is.

Wat is de hoogste kans (in %) op milde bijwerkingen waarbij u nog gentherapie zou kiezen? (let op: dit is dus altijd hoger dan 80, maar kan ook 100 zijn indien u altijd voor gentherapie zou kiezen)

Volgende

Heeft u op dit moment of in het verleden een of meerdere van de **milde bijwerkingen** (gehad) die bij uw huidige therapie kunnen voorkomen?

- ◯ Ja
- ◯ Nee

Volgende

Deel 2: Ernstige bijwerkingen

Bij dit onderdeel van de vragenlijst gaat het om ernstige bijwerkingen die kunnen optreden bij het gebruik van enzymtherapie of bij gentherapie. Deze bijwerkingen hebben een ziekenhuisopname tot gevolg.

Volgende

Bekijk de informatie in onderstaande tabel.

Bij enzymtherapie treden ernstige bijwerkingen op bij 0% (0 van de 100) van de vrouwen. Stel dat bij gentherapie bij 20% (20 van de 100) van de vrouwen ernstige bijwerkingen optreden.

Let op: u kunt met uw muis over de kenmerken aan de linkerkant van de tabel gaan om de uitleg hiervan opnieuw te lezen.

	**Enzymtherapie**	**Gentherapie**
**Werkzaamheid**	**Even goed als gentherapie**	**Even goed als enzymtherapie**
**Kans op milde bijwerkingen**	**15% (15 van elke 100)**	**60% (60 van elke 100)**
**Kans op ernstige bijwerkingen**	**0% (0 van elke 100)**	**20% (20 van elke 100)**
**Kans dat aanvullende medicatie nodig is vanwege de therapie**	**0% (0 van elke 100)**	**15% (15 van elke 100)**
**Behandelfrequentie**	**Tweewekelijks, langdurig (jaren)**	**Eenmalig**

Op basis van de informatie in bovenstaande tabel, welke behandeling heeft dan uw voorkeur?

- ◯ Enzymtherapie
- ◯ Gentherapie

Volgende

Bekijk de informatie in onderstaande tabel.

Bij enzymtherapie treden ernstige bijwerkingen op bij 0% (0 van de 100) van de vrouwen. Stel dat bij gentherapie bij 5% (5 van de 100) van de vrouwen ernstige bijwerkingen optreden.

Let op: u kunt met uw muis over de kenmerken aan de linkerkant van de tabel gaan om de uitleg hiervan opnieuw te lezen.

	**Enzymtherapie**	**Gentherapie**
**Werkzaamheid**	**Even goed als gentherapie**	**Even goed als enzymtherapie**
**Kans op milde bijwerkingen**	**15% (15 van elke 100)**	**60% (60 van elke 100)**
**Kans op ernstige bijwerkingen**	**0% (0 van elke 100)**	**5% (5 van elke 100)**
**Kans dat aanvullende medicatie nodig is vanwege de therapie**	**0% (0 van elke 100)**	**15% (15 van elke 100)**
**Behandelfrequentie**	**Tweewekelijks, langdurig (jaren)**	**Eenmalig**

Op basis van de informatie in bovenstaande tabel, welke behandeling heeft dan uw voorkeur?

- ○ Enzymtherapie
- ○ Gentherapie

Volgende

Bekijk de informatie in onderstaande tabel.

Bij enzymtherapie treden ernstige bijwerkingen op bij 0% (0 van de 100) van de vrouwen. Stel dat bij gentherapie bij 10% (10 van de 100) van de vrouwen ernstige bijwerkingen optreden.

Let op: u kunt met uw muis over de kenmerken aan de linkerkant van de tabel gaan om de uitleg hiervan opnieuw te lezen.

	**Enzymtherapie**	**Gentherapie**
**Werkzaamheid**	**Even goed als gentherapie**	**Even goed als enzymtherapie**
**Kans op milde bijwerkingen**	**15% (15 van elke 100)**	**60% (60 van elke 100)**
**Kans op ernstige bijwerkingen**	**0% (0 van elke 100)**	**10% (10 van elke 100)**
**Kans dat aanvullende medicatie nodig is vanwege de therapie**	**0% (0 van elke 100)**	**15% (15 van elke 100)**
**Behandelfrequentie**	**Tweewekelijks, langdurig (jaren)**	**Eenmalig**

Op basis van de informatie in bovenstaande tabel, welke behandeling heeft dan uw voorkeur?

- ◯ Enzymtherapie
- ◯ Gentherapie

Volgende

Bekijk de informatie in onderstaande tabel.

Bij enzymtherapie treden ernstige bijwerkingen op bij 0% (0 van de 100) van de vrouwen. Stel dat bij gentherapie bij 80% (80 van de 100) van de vrouwen ernstige bijwerkingen optreden.

Let op: u kunt met uw muis over de kenmerken aan de linkerkant van de tabel gaan om de uitleg hiervan opnieuw te lezen.

	**Enzymtherapie**	**Gentherapie**
**Werkzaamheid**	**Even goed als gentherapie**	**Even goed als enzymtherapie**
**Kans op milde bijwerkingen**	**15% (15 van elke 100)**	**60% (60 van elke 100)**
**Kans op ernstige bijwerkingen**	**0% (0 van elke 100)**	**80% (80 van elke 100)**
**Kans dat aanvullende medicatie nodig is vanwege de therapie**	**0% (0 van elke 100)**	**15% (15 van elke 100)**
**Behandelfrequentie**	**Tweewekelijks, langdurig (jaren)**	**Eenmalig**

Op basis van de informatie in bovenstaande tabel, welke behandeling heeft dan uw voorkeur?

- Enzymtherapie
- Gentherapie

Volgende

Bekijk de informatie in onderstaande tabel.

Bij enzymtherapie treden ernstige bijwerkingen op bij 0% (0 van de 100) van de vrouwen. Stel dat bij gentherapie bij 50% (50 van de 100) van de vrouwen ernstige bijwerkingen optreden.

Let op: u kunt met uw muis over de kenmerken aan de linkerkant van de tabel gaan om de uitleg hiervan opnieuw te lezen.

	**Enzymtherapie**	**Gentherapie**
**Werkzaamheid**	**Even goed als gentherapie**	**Even goed als enzymtherapie**
**Kans op milde bijwerkingen**	**15% (15 van elke 100)**	**60% (60 van elke 100)**
**Kans op ernstige bijwerkingen**	**0% (0 van elke 100)**	**50% (50 van elke 100)**
**Kans dat aanvullende medicatie nodig is vanwege de therapie**	**0% (0 van elke 100)**	**15% (15 van elke 100)**
**Behandelfrequentie**	**Tweewekelijks, langdurig (jaren)**	**Eenmalig**

Op basis van de informatie in bovenstaande tabel, welke behandeling heeft dan uw voorkeur?

- Enzymtherapie
- Gentherapie

Volgende

U heeft net aangegeven dat u voor enzymtherapie zou kiezen als de kans op ernstige bijwerkingen bij gentherapie 5% is.

Wat is de hoogste kans (in %) op ernstige bijwerkingen waarbij u toch gentherapie zou kiezen? (let op: dit is dus altijd lager dan 5, maar kan ook 0 zijn indien u bijvoorbeeld helemaal geen gentherapie wil)

Volgende

U heeft net aangegeven dat u voor gentherapie zou kiezen als de kans op ernstige bijwerkingen 80% is.

Wat is de hoogste kans (in %) op ernstige bijwerkingen waarbij u nog gentherapie zou kiezen? (let op: dit is dus altijd hoger dan 80, maar kan ook 100 zijn indien u altijd voor gentherapie zou kiezen)

Volgende

Heeft u op dit moment of in het verleden een of meerdere van de **ernstige bijwerkingen** (gehad) die bij uw huidige therapie kunnen voorkomen?

- ◯ Ja
- ◯ Nee

Volgende

Deel 3: gebruik van aanvullende medicatie

Bij dit onderdeel van de vragenlijst gaat het om het gebruik van aanvullende medicatie, wat nodig kan zijn om de bijwerkingen van enzymtherapie of gentherapie te onderdrukken. Dit kan zowel kortdurend als langdurig zijn.

Volgende

Bekijk de informatie in onderstaande tabel.

Bij enzymtherapie is het gebruik van aanvullende medicatie nodig bij 0% (0 van de 100) van de vrouwen. Stel dat bij gentherapie bij 15% (15 van de 100) van de vrouwen aanvullende medicatie nodig zou zijn.

Let op: u kunt met uw muis over de kenmerken aan de linkerkant van de tabel gaan om de uitleg hiervan opnieuw te lezen.

	**Enzymtherapie**	**Gentherapie**
**Werkzaamheid**	**Even goed als gentherapie**	**Even goed als enzymtherapie**
**Kans op milde bijwerkingen**	**15% (15 van elke 100)**	**60% (60 van elke 100)**
**Kans op ernstige bijwerkingen**	**0% (0 van elke 100)**	**20% (20 van elke 100)**
**Kans dat aanvullende medicatie nodig is vanwege de therapie**	**0% (0 van elke 100)**	**15% (15 van elke 100)**
**Behandelfrequentie**	**Tweewekelijks, langdurig (jaren)**	**Eenmalig**

Op basis van de informatie in bovenstaande tabel, welke behandeling heeft dan uw voorkeur?

- ◯ Enzymtherapie
- ◯ Gentherapie

Volgende

Bekijk de informatie in onderstaande tabel.

Bij enzymtherapie is het gebruik van aanvullende medicatie nodig bij 0% (0 van de 100) van de vrouwen. Stel dat bij gentherapie bij 5% (5 van de 100) van de vrouwen aanvullende medicatie nodig zou zijn.

Let op: u kunt met uw muis over de kenmerken aan de linkerkant van de tabel gaan om de uitleg hiervan opnieuw te lezen.

	**Enzymtherapie**	**Gentherapie**
**Werkzaamheid**	**Even goed als gentherapie**	**Even goed als enzymtherapie**
**Kans op milde bijwerkingen**	**15% (15 van elke 100)**	**60% (60 van elke 100)**
**Kans op ernstige bijwerkingen**	**0% (0 van elke 100)**	**20% (20 van elke 100)**
**Kans dat aanvullende medicatie nodig is vanwege de therapie**	**0% (0 van elke 100)**	**5% (5 van elke 100)**
**Behandelfrequentie**	**Tweewekelijks, langdurig (jaren)**	**Eenmalig**

Op basis van de informatie in bovenstaande tabel, welke behandeling heeft dan uw voorkeur?

- ○ Enzymtherapie
- ○ Gentherapie

Volgende

Bekijk de informatie in onderstaande tabel.

Bij enzymtherapie is het gebruik van aanvullende medicatie nodig bij 0% (0 van de 100) van de vrouwen. Stel dat bij gentherapie bij 10% (10 van de 100) van de vrouwen aanvullende medicatie nodig zou zijn.

Let op: u kunt met uw muis over de kenmerken aan de linkerkant van de tabel gaan om de uitleg hiervan opnieuw te lezen.

	**Enzymtherapie**	**Gentherapie**
**Werkzaamheid**	**Even goed als gentherapie**	**Even goed als enzymtherapie**
**Kans op milde bijwerkingen**	**15% (15 van elke 100)**	**60% (60 van elke 100)**
**Kans op ernstige bijwerkingen**	**0% (0 van elke 100)**	**20% (20 van elke 100)**
**Kans dat aanvullende medicatie nodig is vanwege de therapie**	**0% (0 van elke 100)**	**10% (10 van elke 100)**
**Behandelfrequentie**	**Tweewekelijks, langdurig (jaren)**	**Eenmalig**

Op basis van de informatie in bovenstaande tabel, welke behandeling heeft dan uw voorkeur?

- ○ Enzymtherapie
- ○ Gentherapie

Volgende

Bekijk de informatie in onderstaande tabel.

Bij enzymtherapie is het gebruik van aanvullende medicatie nodig bij 0% (0 van de 100) van de vrouwen. Stel dat bij gentherapie bij 50% (50 van de 100) van de vrouwen aanvullende medicatie nodig zou zijn.

Let op: u kunt met uw muis over de kenmerken aan de linkerkant van de tabel gaan om de uitleg hiervan opnieuw te lezen.

	**Enzymtherapie**	**Gentherapie**
**Werkzaamheid**	**Even goed als gentherapie**	**Even goed als enzymtherapie**
**Kans op milde bijwerkingen**	**15% (15 van elke 100)**	**60% (60 van elke 100)**
**Kans op ernstige bijwerkingen**	**0% (0 van elke 100)**	**20% (20 van elke 100)**
**Kans dat aanvullende medicatie nodig is vanwege de therapie**	**0% (0 van elke 100)**	**50% (50 van elke 100)**
**Behandelfrequentie**	**Tweewekelijks, langdurig (jaren)**	**Eenmalig**

Op basis van de informatie in bovenstaande tabel, welke behandeling heeft dan uw voorkeur?

- ◯ Enzymtherapie
- ◯ Gentherapie

Volgende

Bekijk de informatie in onderstaande tabel.

Bij enzymtherapie is het gebruik van aanvullende medicatie nodig bij 0% (0 van de 100) van de vrouwen. Stel dat bij gentherapie bij 30% (30 van de 100) van de vrouwen aanvullende medicatie nodig zou zijn.

Let op: u kunt met uw muis over de kenmerken aan de linkerkant van de tabel gaan om de uitleg hiervan opnieuw te lezen.

	**Enzymtherapie**	**Gentherapie**
**Werkzaamheid**	**Even goed als gentherapie**	**Even goed als enzymtherapie**
**Kans op milde bijwerkingen**	**15% (15 van elke 100)**	**60% (60 van elke 100)**
**Kans op ernstige bijwerkingen**	**0% (0 van elke 100)**	**20% (20 van elke 100)**
**Kans dat aanvullende medicatie nodig is vanwege de therapie**	**0% (0 van elke 100)**	**30% (30 van elke 100)**
**Behandelfrequentie**	**Tweewekelijks, langdurig (jaren)**	**Eenmalig**

Op basis van de informatie in bovenstaande tabel, welke behandeling heeft dan uw voorkeur?

- ◯ Enzymtherapie
- ◯ Gentherapie

Volgende

U heeft net aangegeven dat u voor enzymtherapie zou kiezen als de kans op aanvullende medicatie bij gentherapie 5% is.

Wat is de hoogste kans (in %) op aanvullende medicatie waarbij u toch gentherapie zou kiezen? (let op: dit is dus altijd lager dan 5, maar kan ook 0 zijn indien u bijvoorbeeld helemaal geen gentherapie wil)

Volgende

U heeft net aangegeven dat u voor gentherapie zou kiezen als de kans op aanvullende medicatie 50% is.

Wat is de hoogste kans (in %) op aanvullende medicatie waarbij u nog gentherapie zou kiezen? (let op: dit is dus altijd hoger dan 50, maar kan ook 100 zijn indien u altijd voor gentherapie zou kiezen)

Volgende

Gebruikt(e) u op dit moment of in het verleden een of meerdere aanvullende medicijnen die door uw therapie nodig zijn?

- ◯ Ja
- ◯ Nee

Volgende

Deel 4: onzekerheidsvraag

Bij de keuze om wel of niet mee te doen aan een studie naar een nieuw geneesmiddel kan ook de onzekerheid over werkzaamheid en bijwerkingen meespelen. Er moet dan gekozen worden tussen therapie waarvan de werkzaamheid (gedeeltelijk) bekend is (enzymtherapie) en een nieuwe therapie in studieverband met meer onzekerheden (gentherapie). Deze vraag gaat over die onzekerheid.

We gaan er in dit scenario van uit dat gentherapie – in ieder geval op korte termijn (tot ca. 2 jaar) - even goed werkt als uw huidige therapie.

Enzymtherapie is voldoende veilig bevonden om op recept voorgeschreven te mogen worden. Voor gentherapie is dat nog niet het geval, dus het wordt alleen nog in studieverband gegeven. Of het veilig en werkzaam is moet nog worden vastgesteld.

Voor de ziekte van Fabry kan pas na jaren worden geconcludeerd of een nieuwe therapie goed werkt omdat de ziekte zich langzaam ontwikkelt.

Volgende

Bekijk de informatie in onderstaande tabel.

Enzymtherapie is voldoende veilig bevonden om op recept voorgeschreven te mogen worden. Stel dat voor gentherapie de kans dat dit veilig en werkzaam blijkt 25% is.

Let op: u kunt met uw muis over de kenmerken aan de linkerkant van de tabel gaan om de uitleg hiervan opnieuw te lezen.

	**Enzymtherapie**	**Gentherapie**
**Werkzaamheid op korte termijn (ca. 2 jaar)**	**Even goed als gentherapie**	**Even goed als enzymtherapie**
**Kans dat het middel op langere termijn als veilig en effectief wordt beschouwd**	**75-100%***	**25%**
**Kans op milde bijwerkingen**	**15% (15 van elke 100)**	**60% (60 van elke 100)**
**Kans op ernstige bijwerkingen**	**0% (0 van elke 100)**	**20% (20 van elke 100)**
**Kans dat aanvullende medicatie nodig is vanwege de therapie**	**0% (0 van elke 100)**	**15% (15 van elke 100)**
**Behandelfrequentie**	**Tweewekelijks, langdurig (jaren)**	**Eenmalig**

*Enzymtherapie is sinds ongeveer 20 jaar beschikbaar en is veilig gebleken. Door de zeldzaamheid en het trage beloop van de ziekte van Fabry leren we nog steeds voor welke patiënten en op welk moment enzymtherapie werkzaam is. Daarom kan dit percentage per persoon iets verschillen.

Op basis van de informatie in bovenstaande tabel, welke behandeling heeft dan uw voorkeur?

- ○ Enzymtherapie
- ○ Gentherapie

Volgende

Bekijk de informatie in onderstaande tabel.

Enzymtherapie is voldoende veilig bevonden om op recept voorgeschreven te mogen worden. Stel dat voor gentherapie de kans dat dit veilig en werkzaam blijkt 5% is.

Let op: u kunt met uw muis over de kenmerken aan de linkerkant van de tabel gaan om de uitleg hiervan opnieuw te lezen.

	**Enzymtherapie**	**Gentherapie**
**Werkzaamheid op korte termijn (ca. 2 jaar)**	**Even goed als gentherapie**	**Even goed als enzymtherapie**
**Kans dat het middel op langere termijn als veilig en effectief wordt beschouwd**	**75%-100%***	**5%**
**Kans op milde bijwerkingen**	**15%<br>(15 van elke 100)**	**60%<br>(60 van elke 100)**
**Kans op ernstige bijwerkingen**	**0%<br>(0 van elke 100)**	**20%<br>(20 van elke 100)**
**Kans dat aanvullende medicatie nodig is vanwege de therapie**	**0%<br>(0 van elke 100)**	**15%<br>(15 van elke 100)**
**Behandelfrequentie**	**Tweewekelijks, langdurig (jaren)**	**Eenmalig**

*Enzymtherapie is sinds ongeveer 20 jaar beschikbaar en is veilig gebleken. Door de zeldzaamheid en het trage beloop van de ziekte van Fabry leren we nog steeds voor welke patiënten en op welk moment enzymtherapie werkzaam is. Daarom kan dit percentage per persoon iets verschillen.

Op basis van de informatie in bovenstaande tabel, welke behandeling heeft dan uw voorkeur?

- ○ Enzymtherapie
- ○ Gentherapie

Volgende

Bekijk de informatie in onderstaande tabel.

Enzymtherapie is voldoende veilig bevonden om op recept voorgeschreven te mogen worden. Stel dat voor gentherapie de kans dat dit veilig en werkzaam blijkt 10% is.

Let op: u kunt met uw muis over de kenmerken aan de linkerkant van de tabel gaan om de uitleg hiervan opnieuw te lezen.

	Enzymtherapie	Gentherapie
Werkzaamheid op korte termijn (ca. 2 jaar))	Even goed als gentherapie	Even goed als enzymtherapie
Kans dat het middel op langere termijn als veilig en effectief wordt beschouwd	75%-100%*	10%
Kans op milde bijwerkingen	15% (15 van elke 100)	60% (60 van elke 100)
Kans op ernstige bijwerkingen	0% (0 van elke 100)	20% (20 van elke 100)
Kans dat aanvullende medicatie nodig is vanwege de therapie	0% (0 van elke 100)	15% (15 van elke 100)
Behandelfrequentie	Tweewekelijks, langdurig (jaren)	Eenmalig

*Enzymtherapie is sinds ongeveer 20 jaar beschikbaar en is veilig gebleken. Door de zeldzaamheid en het trage beloop van de ziekte van Fabry leren we nog steeds voor welke patiënten en op welk moment enzymtherapie werkzaam is. Daarom kan dit percentage per persoon iets verschillen.

Op basis van de informatie in bovenstaande tabel, welke behandeling heeft dan uw voorkeur?

- ○ Enzymtherapie
- ○ Gentherapie

Volgende

Bekijk de informatie in onderstaande tabel.

Enzymtherapie is voldoende veilig bevonden om op recept voorgeschreven te mogen worden. Stel dat voor gentherapie de kans dat dit veilig en werkzaam blijkt 50% is.

Let op: u kunt met uw muis over de kenmerken aan de linkerkant van de tabel gaan om de uitleg hiervan opnieuw te lezen.

	**Enzymtherapie**	**Gentherapie**
**Werkzaamheid op korte termijn (ca. 2 jaar))**	**Even goed als gentherapie**	**Even goed als enzymtherapie**
**Kans dat het middel op langere termijn als veilig en effectief wordt geschouwd**	**75%-100%**	**50%**
**Kans op milde bijwerkingen**	**15% (15 van elke 100)**	**60% (60 van elke 100)**
**Kans op ernstige bijwerkingen**	**0% (0 van elke 100)**	**20% (20 van elke 100)**
**Kans dat aanvullende medicatie nodig is vanwege de therapie**	**0% (0 van elke 100)**	**15% (15 van elke 100)**
**Behandelfrequentie**	**Tweewekelijks, langdurig (jaren)**	**Eenmalig**

*Enzymtherapie is sinds ongeveer 20 jaar beschikbaar en is veilig gebleken. Door de zeldzaamheid en het trage beloop van de ziekte van Fabry leren we nog steeds voor welke patiënten en op welk moment enzymtherapie werkzaam is. Daarom kan dit percentage per persoon iets verschillen.

Op basis van de informatie in bovenstaande tabel, welke behandeling heeft dan uw voorkeur?

- ◯ Enzymtherapie
- ◯ Gentherapie

Volgende

Bekijk de informatie in onderstaande tabel.

Enzymtherapie is voldoende veilig bevonden om op recept voorgeschreven te mogen worden. Stel dat voor gentherapie de kans dat dit veilig en werkzaam blijkt 30% is.

Let op: u kunt met uw muis over de kenmerken aan de linkerkant van de tabel gaan om de uitleg hiervan opnieuw te lezen.

	**Enzymtherapie**	**Gentherapie**
**Werkzaamheid op korte termijn (ca. 2 jaar))**	**Even goed als gentherapie**	**Even goed als enzym therapie**
**Kans dat het middel op langere termijn als veilig en effectief wordt beschouwd**	**75%-100%***	**30%**
**Kans op milde bijwerkingen**	**15% (15 van elke 100)**	**60% (60 van elke 100)**
**Kans op ernstige bijwerkingen**	**0% (0 van elke 100)**	**20% (20 van elke 100)**
**Kans dat aanvullende medicatie nodig is vanwege de therapie**	**0% (0 van elke 100)**	**15% (15 van elke 100)**
**Behandelfrequentie**	**Tweewekelijks, langdurig (jaren)**	**Eenmalig**

*Enzymtherapie is sinds ongeveer 20 jaar beschikbaar en is veilig gebleken. Door de zeldzaamheid en het trage beloop van de ziekte van Fabry leren we nog steeds voor welke patiënten en op welk moment enzymtherapie werkzaam is. Daarom kan dit percentage per persoon iets verschillen.

Op basis van de informatie in bovenstaande tabel, welke behandeling heeft dan uw voorkeur?

- ○ Enzymtherapie
- ○ Gentherapie

Volgende

U heeft net aangegeven dat u voor gentherapie zou kiezen als de kans dat gentherapie in de komende jaren als veilig en effectief wordt beschouwd 5% is.

Wat is de minimale kans (in %) dat het middel in de komende jaren als veilig en effectief wordt beschouwd waarbij u nog voor gentherapie zou kiezen? (let op: dit is dus altijd lager dan 5, maar kan ook 0 zijn indien u bijvoorbeeld helemaal geen gentherapie wil)

Volgende

U heeft net aangegeven dat u voor enzymtherapie zou kiezen als de kans dat gentherapie in de komende jaren als veilig en effectief wordt beschouwd 50% is.

Wat is de minimale kans (in %) dat het middel in de komende jaren als veilig en effectief wordt beschouwd waarbij u toch voor gentherapie zou kiezen? (let op: dit is dus altijd hoger dan 50, maar kan ook 100 zijn indien u altijd voor gentherapie zou kiezen)

Volgende

Deel 5: Stel gentherapie werkt beter

We gaan er in dit scenario van uit dat gentherapie **een beter effect** heeft dan enzymtherapie. Of gentherapie veilig en werkzaam is, moet uitgezocht worden in studieverband.

Volgende

Bekijk de informatie in onderstaande tabel.

Enzymtherapie is voldoende veilig bevonden om op recept voorgeschreven te mogen worden. Stel dat voor gentherapie de kans dat dit veilig en werkzaam blijkt 25% is.

Let op: u kunt met uw muis over de kenmerken aan de rechterkant van de tabel gaan om de uitleg hiervan opnieuw te lezen.

	Enzymtherapie	Gentherapie
**Werkzaamheid op korte termijn (ca. 2 jaar)**	**Minder goed dan gentherapie**	**Beter dan enzymtherapie**
**Kans dat het middel op langere termijn als veilig en effectief wordt beschouwd**	**75%-100%***	**25%**
**Kans op milde bijwerkingen**	**15% (15 van elke 100)**	**60% (60 van elke 100)**
**Kans op ernstige bijwerkingen**	**0% (0 van elke 100)**	**20% (20 van elke 100)**
**Kans dat aanvullende medicatie nodig is vanwege de therapie**	**0% (0 van elke 100)**	**15% (15 van elke 100)**
**Behandelfrequentie**	**Tweewekelijks, langdurig (jaren)**	**Eenmalig**

*Enzymtherapie is sinds ongeveer 20 jaar beschikbaar en is veilig gebleken. Door de zeldzaamheid en het trage beloop van de ziekte van Fabry leren we nog steeds voor welke patiënten en op welk moment enzymtherapie werkzaam is. Daarom kan dit percentage per persoon iets verschillen.

Op basis van de informatie in bovenstaande tabel, welke behandeling heeft dan uw voorkeur?

- ◯ Enzymtherapie
- ◯ Gentherapie

Volgende

Bekijk de informatie in onderstaande tabel.

Enzymtherapie is voldoende veilig bevonden om op recept voorgeschreven te mogen worden. Stel dat voor gentherapie de kans dat dit veilig en werkzaam blijkt 5% is.

Let op: u kunt met uw muis over de kenmerken aan de linkerkant van de tabel gaan om de uitleg hiervan opnieuw te lezen.

	**Enzymtherapie**	**Gentherapie**
**Werkzaamheid op korte termijn (ca. 2 jaar)**	**Minder goed dan gentherapie**	**Beter dan enzymtherapie**
**Kans dat het middel op langere termijn als veilig en effectief wordt beschouwd**	**75%-100%***	**5%**
**Kans op milde bijwerkingen**	**15% (15 van elke 100)**	**60% (60 van elke 100)**
**Kans op ernstige bijwerkingen**	**0% (0 van elke 100)**	**20% (20 van elke 100)**
**Kans dat aanvullende medicatie nodig is vanwege de therapie**	**0% (0 van elke 100)**	**15% (15 van elke 100)**
**Behandelfrequentie**	**Tweewekelijks, langdurig (jaren)**	**Eenmalig**

*Enzymtherapie is sinds ongeveer 20 jaar beschikbaar en is veilig gebleken. Door de zeldzaamheid en het trage beloop van de ziekte van Fabry leren we nog steeds voor welke patiënten en op welk moment enzymtherapie werkzaam is. Daarom kan dit percentage per persoon iets verschillen.

Op basis van de informatie in bovenstaande tabel, welke behandeling heeft dan uw voorkeur?

- ○ Enzymtherapie
- ○ Gentherapie

Volgende

Bekijk de informatie in onderstaande tabel.

Enzymtherapie is voldoende veilig bevonden om op recept voorgeschreven te mogen worden. Stel dat voor gentherapie de kans dat dit veilig en werkzaam blijkt 10% is.

Let op: u kunt met uw muis over de kenmerken aan de linkerkant van de tabel gaan om de uitleg hiervan opnieuw te lezen.

	Enzymtherapie	Gentherapie
**Werkzaamheid op korte termijn (ca. 2 jaar)**	**Minder goed dan gentherapie**	**Beter dan enzymtherapie**
**Kans dat het middel op langere termijn als veilig en effectief wordt beschouwd**	**75%-100%***	**10%**
**Kans op milde bijwerkingen**	**15% (15 van elke 100)**	**60% (60 van elke 100)**
**Kans op ernstige bijwerkingen**	**0% (0 van elke 100)**	**20% (20 van elke 100)**
**Kans dat aanvullende medicatie nodig is vanwege de therapie**	**0% (0 van elke 100)**	**15% (15 van elke 100)**
**Behandelfrequentie**	**Tweewekelijks, langdurig (jaren)**	**Eenmalig**

*Enzymtherapie is sinds ongeveer 20 jaar beschikbaar en is veilig gebleken. Door de zeldzaamheid en het trage beloop van de ziekte van Fabry leren we nog steeds voor welke patiënten en op welk moment enzymtherapie werkzaam is. Daarom kan dit percentage per persoon iets verschillen.

Op basis van de informatie in bovenstaande tabel, welke behandeling heeft dan uw voorkeur?

- Enzymtherapie
- Gentherapie

Volgende

Bekijk de informatie in onderstaande tabel.

Enzymtherapie is voldoende veilig bevonden om op recept voorgeschreven te mogen worden. Stel dat voor gentherapie de kans dat dit veilig en werkzaam blijkt 50% is.

Let op: u kunt met uw muis over de kenmerken aan de linkerkant van de tabel gaan om de uitleg hiervan opnieuw te lezen.

	Enzymtherapie	Gentherapie
**Werkzaamheid op korte termijn (ca. 2 jaar)**	**Minder goed dan gentherapie**	**Beter dan enzymtherapie**
**Kans dat het middel op langere termijn als veilig en effectief wordt beschouwd**	**75%-100%***	**50%**
**Kans op milde bijwerkingen**	**15%<br>(15 van elke 100)**	**60%<br>(60 van elke 100)**
**Kans op ernstige bijwerkingen**	**0%<br>(0 van elke 100)**	**20%<br>(20 van elke 100)**
**Kans dat aanvullende medicatie nodig is vanwege de therapie**	**0%<br>(0 van elke 100)**	**15%<br>(15 van elke 100)**
**Behandelfrequentie**	**Tweewekelijks, langdurig (jaren)**	**Eenmalig**

*Enzymtherapie is sinds ongeveer 20 jaar beschikbaar en is veilig gebleken. Door de zeldzaamheid en het trage beloop van de ziekte van Fabry leren we nog steeds voor welke patiënten en op welk moment enzymtherapie werkzaam is. Daarom kan dit percentage per persoon iets verschillen.

Op basis van de informatie in bovenstaande tabel, welke behandeling heeft dan uw voorkeur?

- Enzymtherapie
- Gentherapie

Volgende

Bekijk de informatie in onderstaande tabel.

Enzymtherapie is voldoende veilig bevonden om op recept voorgeschreven te mogen worden. Stel dat voor gentherapie de kans dat dit veilig en werkzaam blijkt 30% is.

Let op: u kunt met uw muis over de kenmerken aan de linkerkant van de tabel gaan om de uitleg hiervan opnieuw te lezen.

	**Enzymtherapie**	**Gentherapie**
**Werkzaamheid op korte termijn (ca. 2 jaar)**	**Minder goed dan gentherapie**	**Beter dan enzymtherapie**
**Kans dat het middel op langere termijn als veilig en effectief wordt beschouwd**	**75%-100%***	**30%**
**Kans op milde bijwerkingen**	**15% (15 van elke 100)**	**60% (60 van elke 100)**
**Kans op ernstige bijwerkingen**	**0% (0 van elke 100)**	**20% (20 van elke 100)**
**Kans dat aanvullende medicatie nodig is vanwege de therapie**	**0% (0 van elke 100)**	**15% (15 van elke 100)**
**Behandelfrequentie**	**Tweewekelijks, langdurig (jaren)**	**Eenmalig**

*Enzymtherapie is sinds ongeveer 20 jaar beschikbaar en is veilig gebleken. Door de zeldzaamheid en het trage beloop van de ziekte van Fabry leren we nog steeds voor welke patiënten en op welk moment enzymtherapie werkzaam is. Daarom kan dit percentage per persoon iets verschillen.

Op basis van de informatie in bovenstaande tabel, welke behandeling heeft dan uw voorkeur?

- ○ Enzymtherapie
- ○ Gentherapie

Volgende

U heeft net aangegeven dat u voor gentherapie zou kiezen als de kans dat gentherapie in de komende jaren als veilig en effectief wordt beschouwd 5% is.

Wat is de minimale kans (in %) dat het middel in de komende jaren als veilig en effectief wordt beschouwd waarbij u nog voor gentherapie zou kiezen? (let op: dit is dus altijd lager dan 5, maar kan ook 0 zijn indien u bijvoorbeeld helemaal geen gentherapie wil)

Volgende

U heeft net aangegeven dat u voor enzymtherapie zou kiezen als de kans dat gentherapie in de komende jaren als veilig en effectief wordt beschouwd 50% is.

Wat is de minimale kans (in %) dat het middel in de komende jaren als veilig en effectief wordt beschouwd waarbij u toch voor gentherapie zou kiezen? (let op: dit is dus altijd hoger dan 50, maar kan ook 100 zijn indien u altijd voor gentherapie zou kiezen)

Volgende

Heeft u ooit aan een studie naar een nieuwe (vorm van) therapie voor de ziekte van Fabry meegewerkt? Studies waar u op dit moment nog aan meewerkt tellen ook mee.

- ◯ Ja
- ◯ Nee

Volgende

Uw mening over de medicijnen die u gebruikt

Als laatste onderdeel van deze vragenlijst willen we u vragen om per vraag het antwoord te kiezen dat het beste uw mening weergeeft over medicatie die u voorgeschreven heeft gekregen of nog krijgt. Het gaat hierbij specifiek om medicatie die u krijgt vanwege de ziekte van Fabry, zoals enzymtherapie, pijnstilling of middelen voor uw bloeddruk, hart of nieren. We vragen u om medicijnen die u eventueel in het kader van andere aandoeningen voorgeschreven krijgt hier niet mee te wegen in uw keuze.

U kunt per vraag een antwoord kiezen.

	Helemaal niet mee eens	Niet mee eens	Geen duidelijke mening	Mee eens	Helemaal mee eens
Op het moment hangt mijn gezondheid af van mijn medicijnen	○	○	○	○	○
Ik maak met zorgen over het feit dat ik medicijnen moet nemen	○	○	○	○	○
Mijn leven zou erg moeilijk zijn zonder medicijnen	○	○	○	○	○
Soms maak ik me zorgen over de effecten die mijn medicijnen op de langere termijn kunnen hebben	○	○	○	○	○
Zonder mijn medicijnen zou ik heel ziek zijn	○	○	○	○	○
Ik ben onvoldoende op de hoogte van wat mijn medicijnen doen	○	○	○	○	○
Mijn toekomstige gezondheid hangt af van mijn medicijnen	○	○	○	○	○
Mijn medicijnen ontwrichten mijn leven	○	○	○	○	○
Soms ben ik bang dat ik te afhankelijk zal worden van mijn medicijnen	○	○	○	○	○
Mijn medicijnen voorkomen dat ik verder achteruit ga	○	○	○	○	○
Deze medicijnen hebben onplezierige bijwerkingen	○	○	○	○	○

Volgende

Afronding vragenlijst & contact

U bent bij het einde van de vragenlijst aangekomen. We willen u van harte bedanken voor uw tijd en het beantwoorden van de vragen. Een samenvatting van onze bevindingen uit dit onderzoek zal na het verwerken van de resultaten met alle deelnemers van dit vragenlijstonderzoek via email worden gedeeld.

Heeft u nog vragen of opmerkingen over de vragenlijst, dit onderzoek of het onderwerp?

Volgende

U gaf aan dat u Migalastat (Galafold©) gebruikt. Om de vragenlijst verder in te vullen en deel te nemen aan dit onderzoek vragen we u vriendelijk contact op te nemen met een van de onderzoekers, Ellie Corazolla (e.m.corazolla@amsterdamumc.nl).

Amsterdam UMC
Universitair Medische Centra

Nogmaals hartelijk dank voor uw medewerking.

Als u aanvullende achtergrond informatie wenst over gentherapie voor stofwisselingsziekten willen we u wijzen op deze uitgebreide informatievideo van patientenvereniging VKS. Klik hier om deze video te bekijken.

PTT survey for men with Fabry disease

Volgende

Doel van dit onderzoek

Uit groepsgesprekken en interviews met patiënten met de ziekte van Fabry is gebleken dat er verschillende factoren zijn die maken of patiënten in de toekomst gentherapie zouden willen ontvangen. De belangrijkste factoren zijn eventuele bijwerkingen van therapie en de te verwachten werkzaamheid (effectiviteit).

Met deze vragenlijst willen wij – samen met VKS (patiëntenvereniging voor volwassenen en kinderen met een erfelijke stofwisselingsziekte) en FSIGN (de Fabry patiëntenvereniging) - onderzoeken welke patiënten wanneer gentherapie zouden willen ontvangen als zij hiervoor (in studieverband) in aanmerking zouden komen.

Om te kunnen onderzoeken in hoeverre deze factoren invloed hebben op de keuzes van patiënten stellen we u vragen over:
- uw huidige situatie
- gentherapie
- geneesmiddelen in het algemeen

Wij vragen u om aan te geven of u onder verschillende omstandigheden zou kiezen voor behandeling met gentherapie of uw huidige therapie. Indien uw huidige behandeling een behandeling in studieverband is dan vragen we u uit te gaan van de situatie voordat u aan de studie meedeed. Meer uitleg hierover vindt u verderop. Voor deze vragenlijst gaan we ervan uit dat u behandeld kunt worden met gentherapie. Of dit in de toekomst daadwerkelijk zo zal zijn is niet bekend.

U kunt de vragenlijst op elk moment sluiten en later op dezelfde plek verdergaan met invullen. Tijdens het invullen van de vragenlijst kunt u niet terug naar vorige vragen. Als u bij het invullen van de vragenlijst hulp wilt of tegen iets aanloopt, sluit de vragenlijst en stuur een mail naar een van de onderzoekers, Ellie Corazolla (e.m.corazolla@amsterdamumc.nl), met de volgende informatie:
- Waar loopt u tegenaan?
- Wilt u via de mail of telefonisch geholpen worden?
- Als u telefonisch contact wenst: Wanneer bent u in de komende dagen bereikbaar? En op welk telefoonnummer?

Volgende

Achtergrondinformatie

Bij de ziekte van Fabry zit er een fout in het erfelijk (genetisch) materiaal (DNA) waardoor een bepaald eiwit (ook wel enzym: alfa-galactosidase) niet of niet goed werkt. Daardoor stapelt er een vetachtige stof in de cellen.

Gentherapie is een vorm van therapie waarbij 'nieuw' erfelijk materiaal in cellen wordt ingebracht. Het doel hiervan is om een stukje DNA toe te voegen zodat er een goed werkend enzym gevormd kan worden. Grofweg zijn er twee soorten gentherapie (zie figuur).

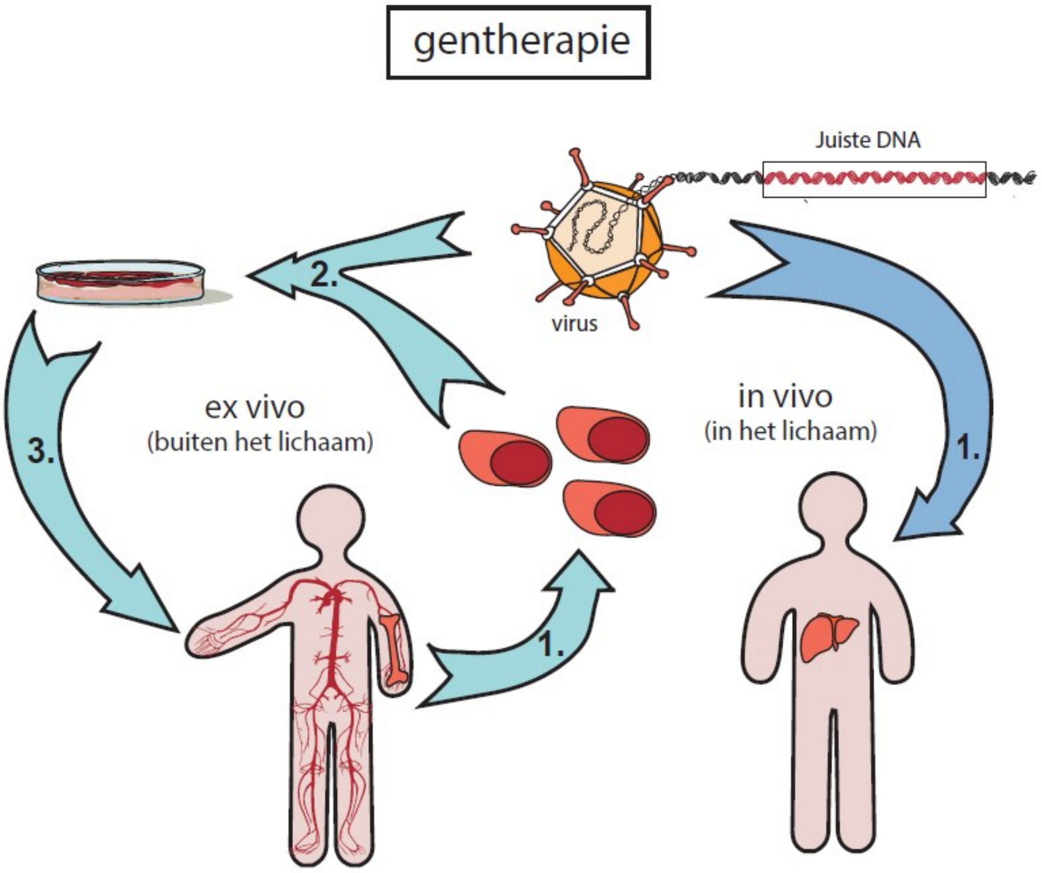

Volgende

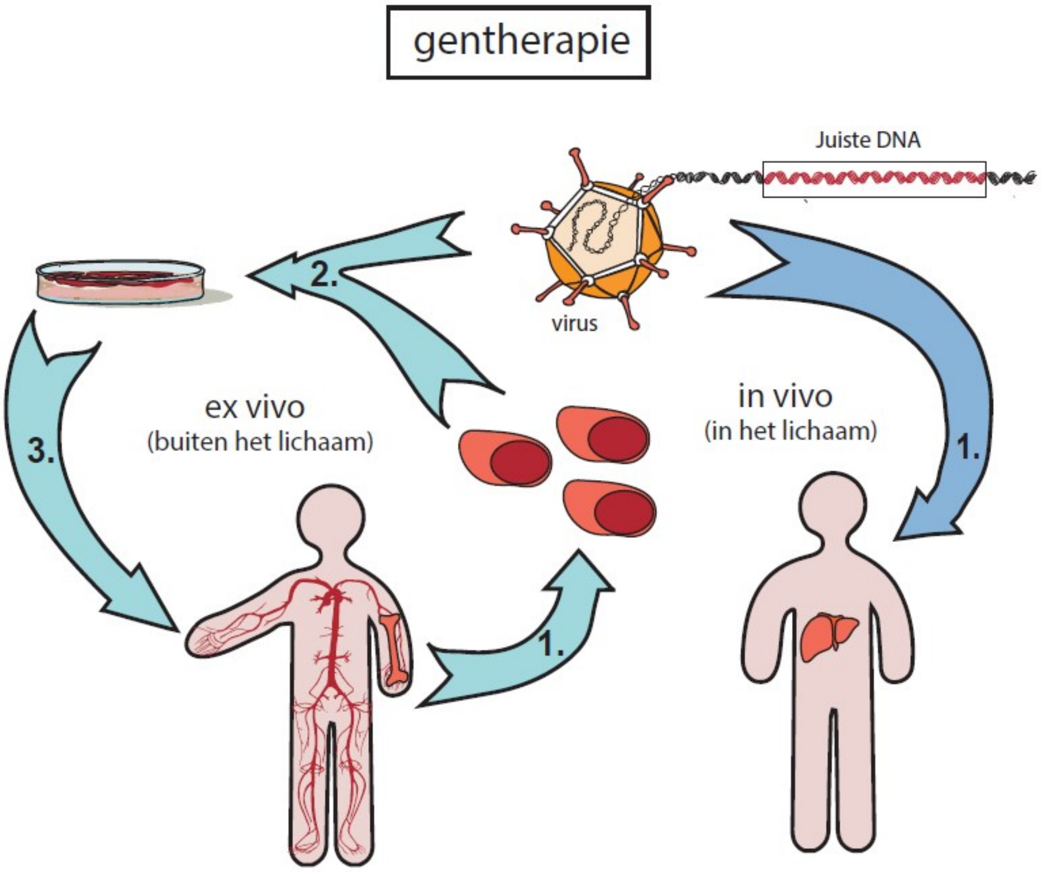

Bij de ene soort ("in vivo") wordt een virusonderdeel waar je niet ziek van wordt in het lichaam gespoten (1.). Dit virusonderdeel heeft het 'nieuwe' DNA bij zich en bouwt dit in bepaalde lichaamscellen in.

- Voordeel van deze vorm van gentherapie: het is een vrij eenvoudige behandeling
- Nadeel van deze vorm van gentherapie: een deel van de mensen hebben het virus al eens heeft 'gehad'. Het lichaam zal het daarom zal herkennen en opruimen. Óf dit bij mensen inderdaad zo is, moet nog worden onderzocht. Áls dit zo is, dan is het de vraag wat het effect van dit 'opruimen' op de werkzaamheid van de therapie is.

De andere soort ("ex vivo") vindt in meerdere stappen plaats:
1. Er worden beenmergcellen uit het lichaam van patiënten gehaald via een bloedafname.
2. Buiten het lichaam worden de cellen behandeld met gentherapie.
3. Daarna worden de behandelde cellen teruggeplaatst in het lichaam.

- Voordelen van deze vorm van gentherapie: er is controle over de hoeveelheid gentherapie waar de specifieke cellen aan worden blootgesteld. Daarnaast kan het lichaam het DNA mogelijk minder makkelijk 'opruimen' omdat het al in de cellen zit.
- Nadelen van deze vorm van gentherapie: er moet eerst ruimte in het beenmerg komen om de 'nieuwe' cellen te laten uitgroeien voordat de cellen teruggeplaatst kunnen worden. Daarom moet er een milde vorm van chemotherapie worden gegeven. Deze behandeling is kortdurend. Daarnaast is er nog geen zekerheid of deze vorm van gentherapie tot blijvende

enzymproductie leidt.

Op dit moment wordt er onderzoek gedaan naar beide soorten gentherapie voor de ziekte van Fabry. Bij de vragen die we u zullen stellen maken we geen onderscheid tussen de verschillende vormen.

Volgende

Achtergrondinformatie

In welke leeftijdcategorie valt u?

- ◯ 18 jaar of jonger
- ◯ Tussen de 18 - 29 jaar
- ◯ Tussen 30 - 39 jaar
- ◯ Tussen 40 - 49 jaar
- ◯ Tussen 50 - 59 jaar
- ◯ Tussen 60 - 69 jaar
- ◯ Tussen 70 - 79 jaar
- ◯ 80 jaar of ouder

Gebruikt u op dit moment medicijnen voor de ziekte van Fabry (hierbij wordt bedoeld: **enzymtherapie** en/of **chaperone therapie** (Migalastat/Galafold©); het gaat hier **niet** om bloeddrukverlagers, bloedverdunners en/of pijnstillers)?

- ◯ Ja
- ◯ Nee

Volgende

Welk medicijn gebruikt u?

- ○ Enzymtherapie: Agalsidase beta (Fabrazyme©)
- ○ Enzymtherapie: Agalsidase alfa (Replagal©)
- ○ Chaperone therapie: Migalastat (Galafold©)
- ○ Medicatie in studieverband

Volgende

Welk medicijn voor de ziekte van Fabry kreeg u voordat u met de studie begon?

- ◯ Enzymtherapie: Agalsidase beta (Fabrazyme©)
- ◯ Enzymtherapie: Agalsidase alfa (Replagal©)

Volgende

Heeft u in het verleden enzymtherapie gehad?

- ◯ Ja, ik heb in het verleden wel behandeling met enzymtherapie (Agalsidase beta/Fabrazyme© of Agalsidase alfa/Replagal©) gehad maar dit is gestopt
- ◯ Nee, ik heb nooit behandeling met enzymtherapie (Agalsidase beta/Fabrazyme© of Agalsidase alfa/Replagal©) gehad

Volgende

Keuzetaken

We vragen u straks steeds een keuze te maken uit twee behandelopties; enzymtherapie of gentherapie. De keuzes zullen erg op elkaar lijken, toch zijn er kleine verschillen. Het is belangrijk voor ons dat u de informatie op de volgende pagina's goed leest en alle keuzetaken invult, we zullen hier de verschillende kenmerken van de behandelingen toelichten.

We vragen bij de keuzetaken om uw persoonlijke mening, er zijn dus geen goede of foute antwoorden/keuzes.

Het is belangrijk om te vermelden dat het mogelijk is om eerder gebruikte therapie weer te hervatten als gentherapie niet goed werkt.

Volgende

Keuzetaken

We vragen u straks steeds een keuze te maken uit twee behandelopties; enzymtherapie of gentherapie. De keuzes zullen erg op elkaar lijken, toch zijn er kleine verschillen. Het is belangrijk voor ons dat u de informatie op de volgende pagina's goed leest en alle keuzetaken invult, we zullen hier de verschillende kenmerken van de behandelingen toelichten.

We vragen bij de keuzetaken om uw persoonlijke mening, er zijn dus geen goede of foute antwoorden/keuzes.

Het is belangrijk om te vermelden dat het mogelijk is om eerder gebruikte therapie weer te hervatten als gentherapie niet goed werkt.

Volgende

Keuzetaken

We vragen u straks steeds een keuze te maken uit twee behandelopties; enzymtherapie of gentherapie. De keuzes zullen erg op elkaar lijken, toch zijn er kleine verschillen. Het is belangrijk voor ons dat u de informatie op de volgende pagina's goed leest en alle keuzetaken invult, we zullen hier de verschillende kenmerken van de behandelingen toelichten.

We vragen bij de keuzetaken om uw persoonlijke mening, er zijn dus geen goede of foute antwoorden/keuzes.

Volgende

Keuzetaken

We vragen u straks steeds een keuze te maken uit twee behandelopties; enzymtherapie of gentherapie. De keuzes zullen erg op elkaar lijken, toch zijn er kleine verschillen. Het is belangrijk voor ons dat u de informatie op de volgende pagina's goed leest en alle keuzetaken invult, we zullen hier de verschillende kenmerken van de behandelingen toelichten.

We vragen bij de keuzetaken om uw persoonlijke mening, er zijn dus geen goede of foute antwoorden/keuzes.

Volgende

Uitleg over de werkzaamheid van de behandeling

We gaan ervan uit dat de gentherapie even goed werkt als uw huidige therapie. Gentherapie is een eenmalige behandeling; bij uw huidige behandeling is het noodzakelijk om herhaaldelijke infusies te ondergaan. Het ondergaan van gentherapie kan gepaard gaan met milde en/of ernstige bijwerkingen. Ook bestaat er een kans dat er extra medicatie gebruikt moet worden. Tot slot is er nog onzekerheid over of gentherapie goed blijft werken op de lange termijn. In de vragenlijst stellen we u vragen die steeds betrekking hebben op een van deze aspecten. We vragen u bij elke vraag een keuze te maken tussen gentherapie en uw huidige therapie.

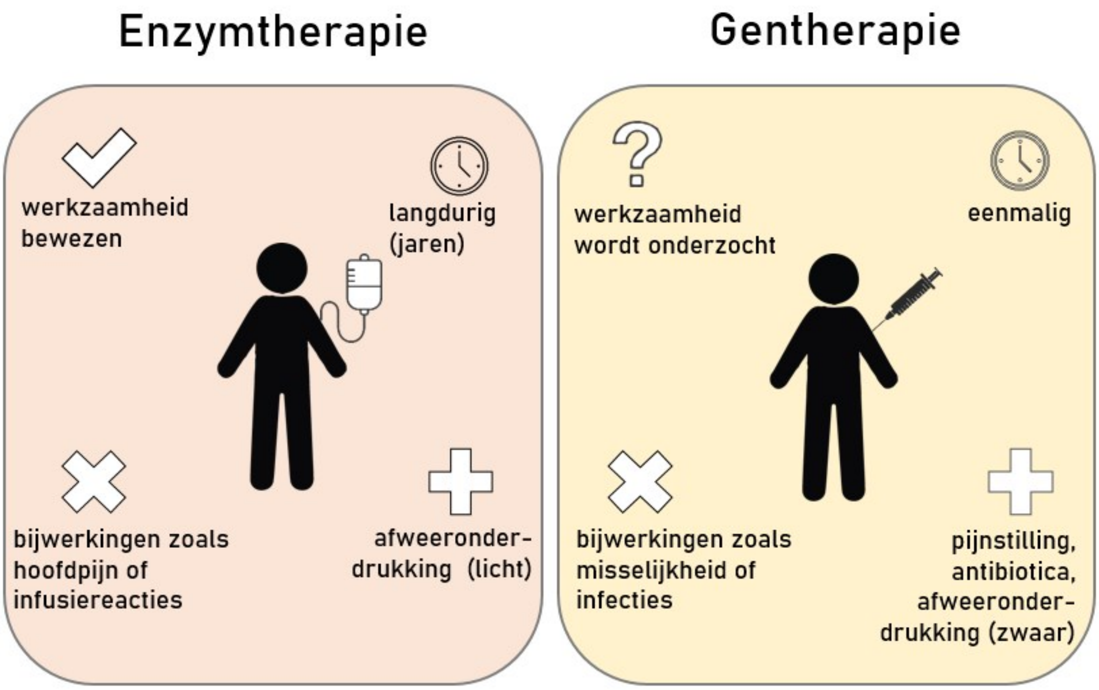

Volgende

Uitleg over de werkzaamheid van de behandeling

U gebruikt op dit moment enzymtherapie in studieverband. We vragen u om voor deze vragenlijst uit te gaan van uw situatie vóórdat u met de studie begon, toen u Agalsidase alfa/Replagal© of Agalsidase beta/Fabrazyme© kreeg.

We gaan ervan uit dat de gentherapie even goed werkt als enzymtherapie. Gentherapie is een eenmalige behandeling; bij enzymtherapie is het noodzakelijk om herhaaldelijke infusies te ondergaan. Het ondergaan van gentherapie kan gepaard gaan met milde en/of ernstige bijwerkingen. Ook bestaat er een kans dat er extra medicatie gebruikt moet worden. Tot slot is er nog onzekerheid over of gentherapie goed blijft werken op de lange termijn. In de vragenlijst stellen we u vragen die steeds betrekking hebben op een van deze aspecten. We vragen u bij elke vraag een keuze te maken tussen gentherapie en de therapie die u in het verleden gebruikte.

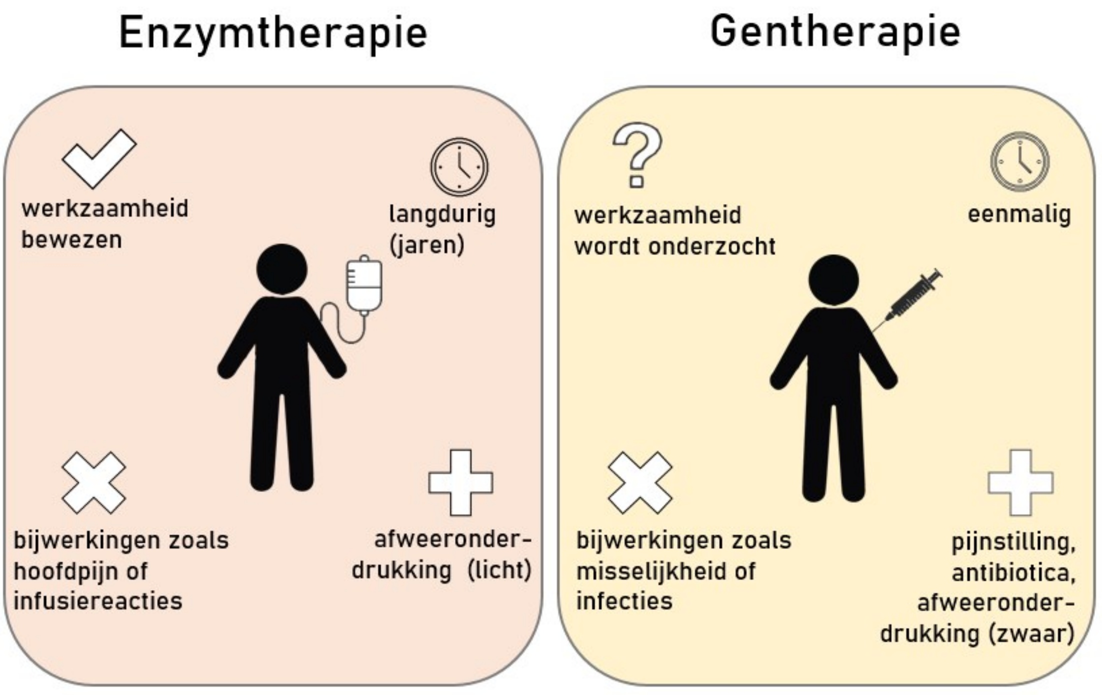

Volgende

Uitleg over de werkzaamheid van de behandeling

U gebruikt op dit moment geen therapie en heeft dat nog nooit eerder gebruikt. We vragen u om er bij het invullen van de vragenlijst van uit te gaan dat u enzymtherapie krijgt. Dat betekent dat u elke twee weken een infuus krijgt met daarin het enzym wat u zelf niet goed aan kunt maken. De eerste infusen krijgt u in het ziekenhuis, daarna kan het thuis met behulp van de thuiszorg. Sommige mannen krijgen bijwerkingen van het infuus, zogenaamde infusiereacties. Klachten die daarbij kunnen passen zijn uitslag, jeuk, zweten, niet lekker worden, misselijkheid en braken of benauwdheid. Soms is het nodig om voor het infuus medicijnen in te nemen om die reacties te onderdrukken.

We gaan ervan uit dat de gentherapie even goed werkt als enzymtherapie. Gentherapie is een eenmalige behandeling; bij enzymtherapie is het noodzakelijk om herhaaldelijke infusies te ondergaan. Het ondergaan van gentherapie kan gepaard gaan met milde en/of ernstige bijwerkingen. Ook bestaat er een kans dat er extra medicatie gebruikt moet worden. Tot slot is er nog onzekerheid over of gentherapie goed blijft werken op de lange termijn. In de vragenlijst stellen we u vragen die steeds betrekking hebben op een van deze aspecten. We vragen u bij elke vraag een keuze te maken tussen gentherapie en enzymtherapie.

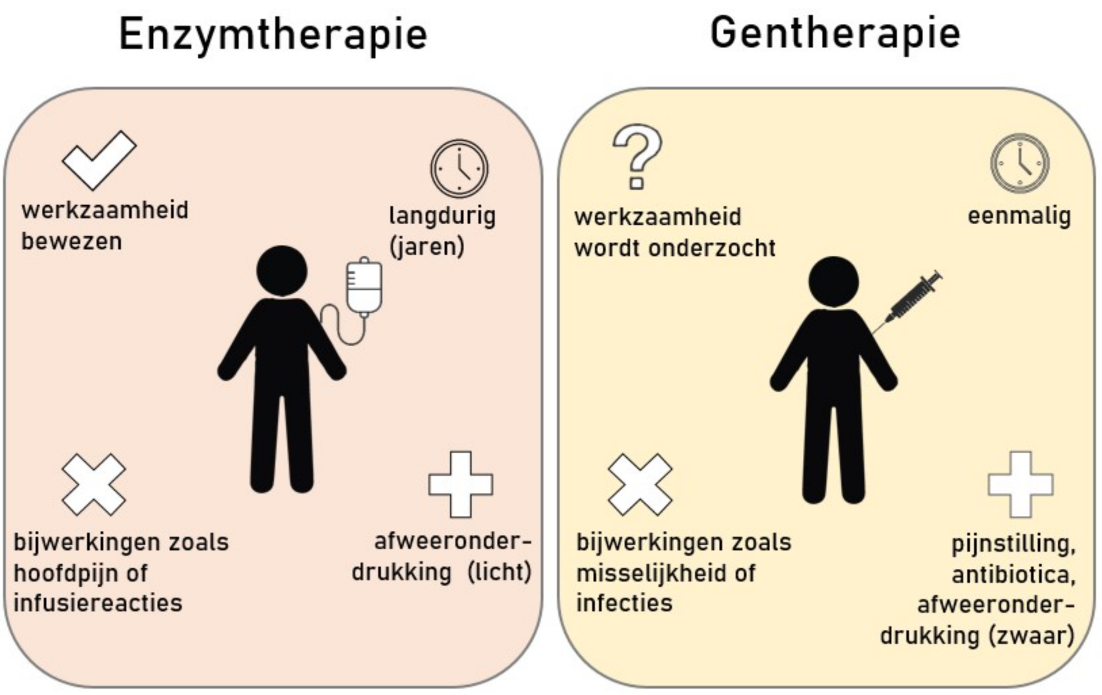

Volgende

Uitleg over de werkzaamheid van de behandeling

U gebruikt op dit moment geen therapie maar heeft dat wel in het verleden gebruikt. We vragen u om voor deze vragenlijst uit te gaan van uw situatie waarin u enzymtherapie (Agalsidase alfa/Replagal© of Agalsidase beta/Fabrazyme©) als tweewekelijks infuus kreeg en de verwachting was dat dat een positief effect had op het ziektebeloop.

We gaan ervan uit dat de gentherapie even goed werkt als enzymtherapie. Dit is het geval wanneer de therapie in een vroeg stadium van de ziekte gegeven wordt. Gentherapie is een eenmalige behandeling; bij enzymtherapie is het noodzakelijk om herhaaldelijke infusies te ondergaan. Het ondergaan van gentherapie kan gepaard gaan met milde en/of ernstige bijwerkingen. Ook bestaat er een kans dat er extra medicatie gebruikt moet worden. Tot slot is er nog onzekerheid over of gentherapie goed blijft werken op de lange termijn. In de vragenlijst stellen we u vragen die steeds betrekking hebben op een van deze aspecten. We vragen u bij elke vraag een keuze te maken tussen gentherapie en de therapie die u in het verleden gebruikte.

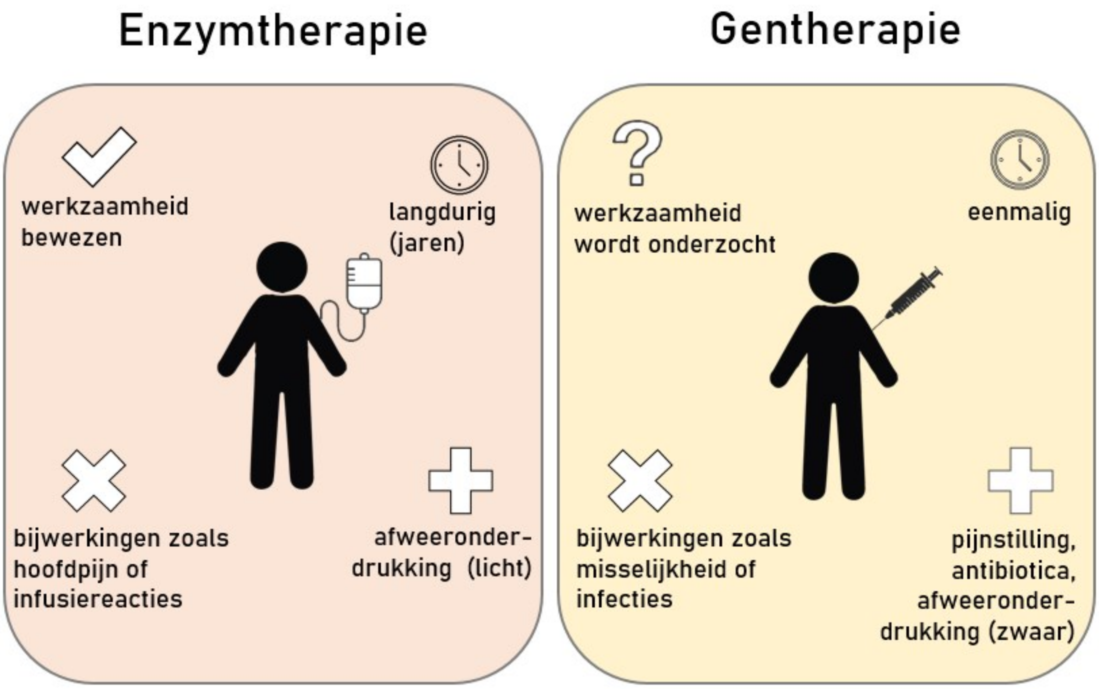

Volgende

Uitleg over mogelijke bijwerkingen van de behandelingen

Milde bijwerkingen: bij het gebruik van enzymtherapie of gentherapie kunnen milde bijwerkingen optreden. Deze bijwerkingen duren kort, zijn zonder blijvende gevolgen en er is geen ziekenhuisopname nodig.

Voor enzymtherapie: reacties op infusies die zich uiten als
- rillingen
- koorts
- hoofdpijn
- misselijkheid
- versnelde hartslag
- lage bloeddruk
- en/of het koud of warm hebben

Voor gentherapie:
- het krijgen van een blauwe plek of bloeduitstorting
- tijdelijk algeheel niet lekker zijn (malaise)
- milde griepachtige klachten
- kortdurende misselijkheid of overgeven
- buikpijn of diarree
- ongevaarlijke infecties
- bloedarmoede
- pijnlijke ontstekingen van het mondslijmvlies
- koorts

Ernstige bijwerkingen: bij het gebruik van enzymtherapie of gentherapie kunnen ernstige bijwerkingen optreden. Deze bijwerkingen hebben een ziekenhuisopname tot gevolg.

Voor enzymtherapie: zeer ernstige infusiereacties, die zich uiten als
- zeer lage bloeddruk
- netelroos
- hoge koorts
- opzwelling van de keel
- versnelde hartslag
- pijn op de borst.

Voor gentherapie: ziekenhuisopnames vanwege bijvoorbeeld
- zeer hoge koorts
- ernstige infecties

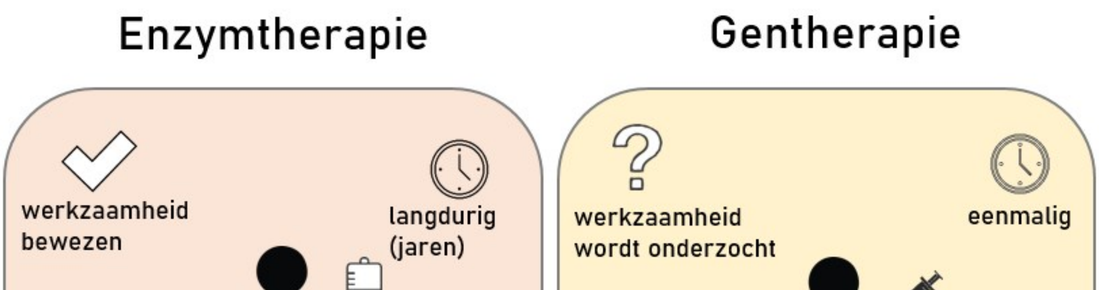

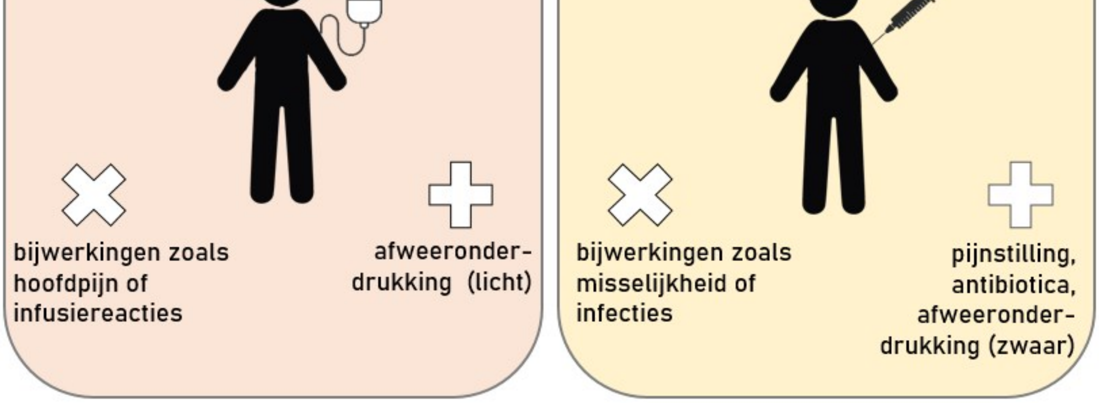
bijwerkingen zoals hoofdpijn of infusiereacties
afweeronder-drukking (licht)
bijwerkingen zoals misselijkheid of infecties
pijnstilling, antibiotica, afweeronder-drukking (zwaar)

Volgende

Uitleg over aanvullende medicatie en behandelfrequentie van de behandelingen.

Aanvullende medicatie: het kan nodig zijn om de bijwerkingen van enzymtherapie of gentherapie te onderdrukken met medicatie. Dit kan zowel kortdurend als langdurig zijn. Met deze medicatie bedoelen we geen medicijnen die gebruikt worden om eventuele al bestaande problemen door de ziekte te behandelen (bijvoorbeeld een middel dat nodig is vanwege verminderde nierfunctie), maar medicijnen die verschijnselen die de therapie veroorzaakt tegengaan (bijvoorbeeld misselijkheid door de gentherapie).

Voor enzymtherapie: wanneer er infusiereacties optreden. Hierbij gaat het om middelen zoals dexamethason, die soms na een paar infusies afgebouwd kunnen worden en soms langdurig voorafgaand aan elke infusie ingenomen moet worden.

Voor gentherapie: Dit kan gaan om een immuunsysteem onderdrukkend middel, zoals prednison. Dat wordt kortdurend (weken tot maanden) gegeven om een afweerreactie van het lichaam tegen het (ongevaarlijke) virusonderdeel tegen te gaan. Daarnaast kan medicatie nodig zijn om bijwerkingen te verhelpen, zoals een antibioticakuur, pijnstilling, of middelen tegen misselijkheid.

Behandelfrequentie: voor enzymtherapie geldt dat u langdurig (jaren) elke twee weken een infuus krijgt terwijl voor gentherapie geldt dat de behandeling eenmalig is.

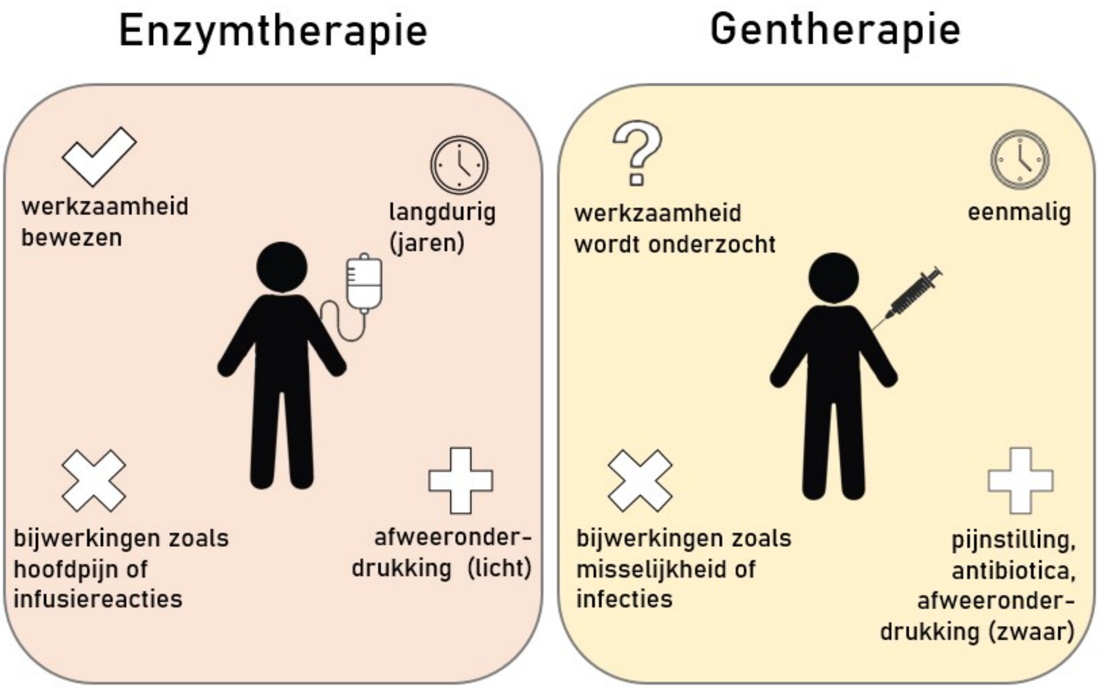

Volgende

Uitleg over waarschijnlijkheid en kansen

Voor verschillende kenmerken van behandelingen wordt straks geproken over 'waarschijnlijkheid'. Hiermee bedoelen we de kans dat de behandeling milde of ernstige bijwerkingen tot gevolg heeft of de kans dat u aanvullende medicatie zou moeten nemen. Er worden verschillende kansen aan u gepresenteerd. Wanneer de waarschijnlijkheid (oftewel kans) op een bijwerking 40% is dan zullen 40 van elke 100 mannen die het medicijn nemen bijwerkingen krijgen terwijl 60 van de 100 mannen die het medicijn nemen geen bijwerkingen zullen ervaren. Dit ziet er als volgt uit:

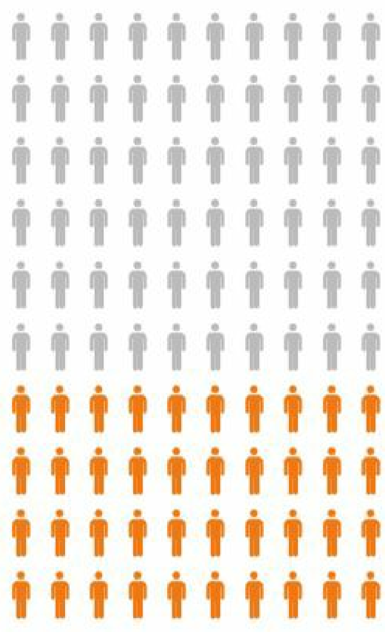

Volgende

Het invullen van de keuzetaken

We vragen u zo meerdere keren een keuze te maken uit twee behandelopties; enzymtherapie of gentherapie.

De behandelingen zullen erg op elkaar lijken, toch zijn er kleine verschillen.

De keuzes zullen eruit zien zoals in het plaatje hieronder. Aan de linkerkant ziet u de eigenschappen van de behandelingen staan. Wanneer u straks de keuzetaken gaat invullen kunt u hier met uw muis op gaat staan, om de uitleg die hierbij hoort nogmaals lezen.

Bekijk de informatie in onderstaande tabel.

Bij enzymtherapie treden ernstige bijwerkingen op bij 15% (15 van de 100) van de mannen. Stel dat bij gentherapie bij 20% (20 van de 100) van de mannen ernstige bijwerkingen optreden.

Let op: u kunt met uw muis over de kenmerken aan de linkerkant van de tabel gaan om de uitleg hiervan opnieuw te lezen.

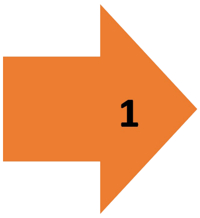

	Enzymtherapie	Gentherapie
Werkzaamheid	Even goed als gentherapie	Even goed als enzymtherapie
Kans op milde bijwerkingen	60% (60 van elke 100)	60% (60 van elke 100)
Kans op ernstige bijwerkingen	15% (15 van elke 100)	20% (20 van elke 100)
Kans dat aanvullende medicatie nodig is vanwege de therapie	5% (5 van elke 100)	15% (15 van elke 100)
Behandelfrequentie	Tweewekelijks, langdurig (jaren)	Eenmalig

Op basis van de informatie in bovenstaande tabel, welke behandeling heeft dan uw voorkeur?

- ○ Enzymtherapie
- ○ Gentherapie

Volgende

In het midden van de taak ziet u de twee behandelingen.

Beide behandelingen hebben net iets andere niveaus van hun eigenschappen.

In het onderstaande voorbeeld werken beide behandelingen even goed. U heeft bij de gentherapie meer kans op ernstige bijwerkingen (namelijk 20% in plaats van 15% voor enzymtherapie), maar gentherapie is een eenmalige behandeling terwijl enzymtherapie uw leven lang tweewekelijks moet worden toegediend.

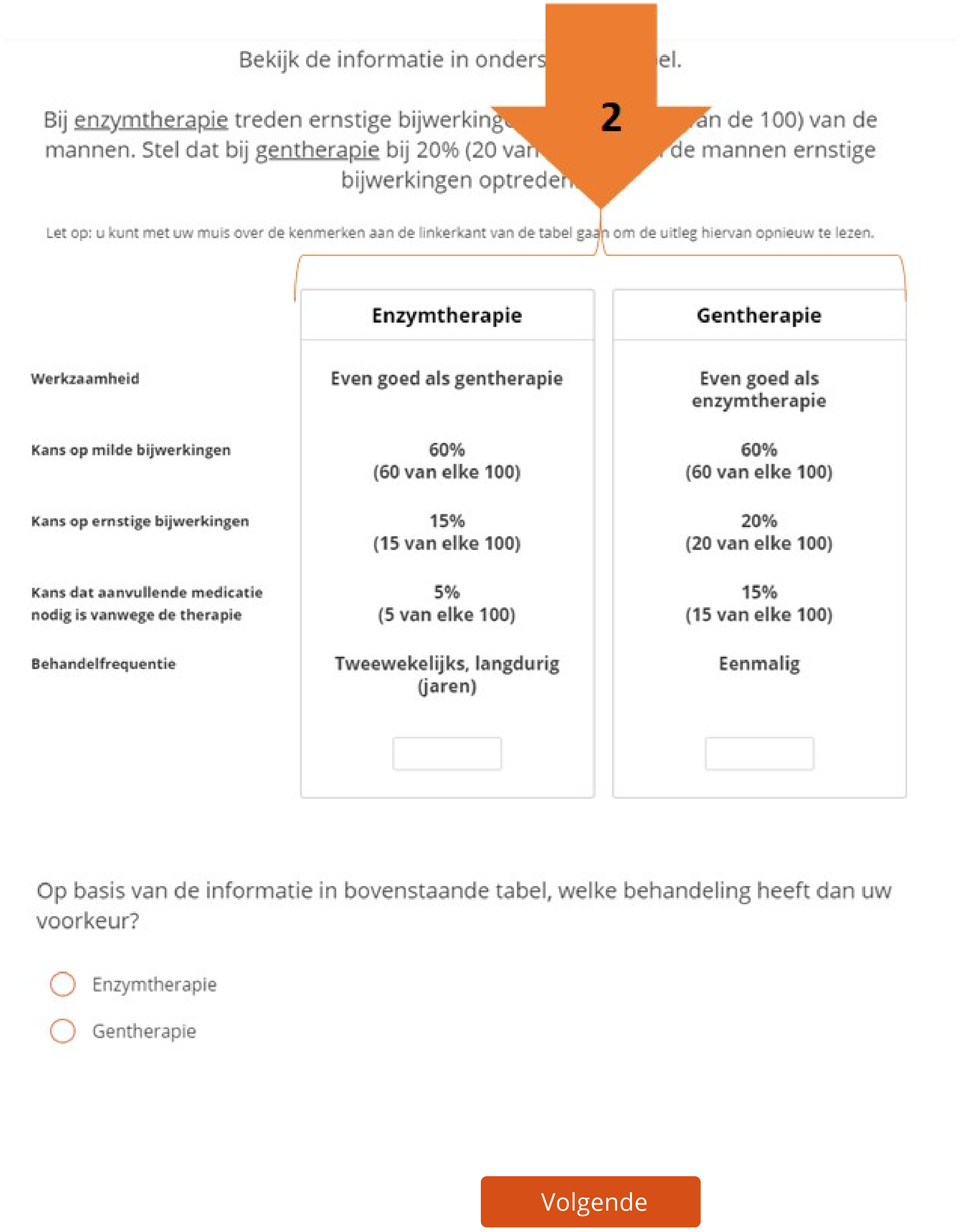
Bekijk de informatie in onders[illegible]el.

Bij enzymtherapie treden ernstige bijwerking[illegible]an de 100) van de mannen. Stel dat bij gentherapie bij 20% (20 van[illegible] de mannen ernstige bijwerkingen optreden[illegible]

Let op: u kunt met uw muis over de kenmerken aan de linkerkant van de tabel gaan om de uitleg hiervan opnieuw te lezen.

	Enzymtherapie	Gentherapie
Werkzaamheid	Even goed als gentherapie	Even goed als enzymtherapie
Kans op milde bijwerkingen	60% (60 van elke 100)	60% (60 van elke 100)
Kans op ernstige bijwerkingen	15% (15 van elke 100)	20% (20 van elke 100)
Kans dat aanvullende medicatie nodig is vanwege de therapie	5% (5 van elke 100)	15% (15 van elke 100)
Behandelfrequentie	Tweewekelijks, langdurig (jaren)	Eenmalig

Op basis van de informatie in bovenstaande tabel, welke behandeling heeft dan uw voorkeur?

- Enzymtherapie
- Gentherapie

Volgende

Vervolgens is het aan u om een afweging te maken tussen deze behandelingen en hun eigenschappen en een keuze te maken.

U kunt uw keuze bevestigen door:
1. In de tabel op de therapie van uw voorkeur te klikken
2. In de vraag onder de tabel dezelfde therapie aan te klikken

Het is belangrijk dat u uw keuze in de tabel nogmaals bevestigt in de vraag onder elke tabel zoals in het voorbeeld hieronder waar de patient voor gentherapie heeft gekozen.

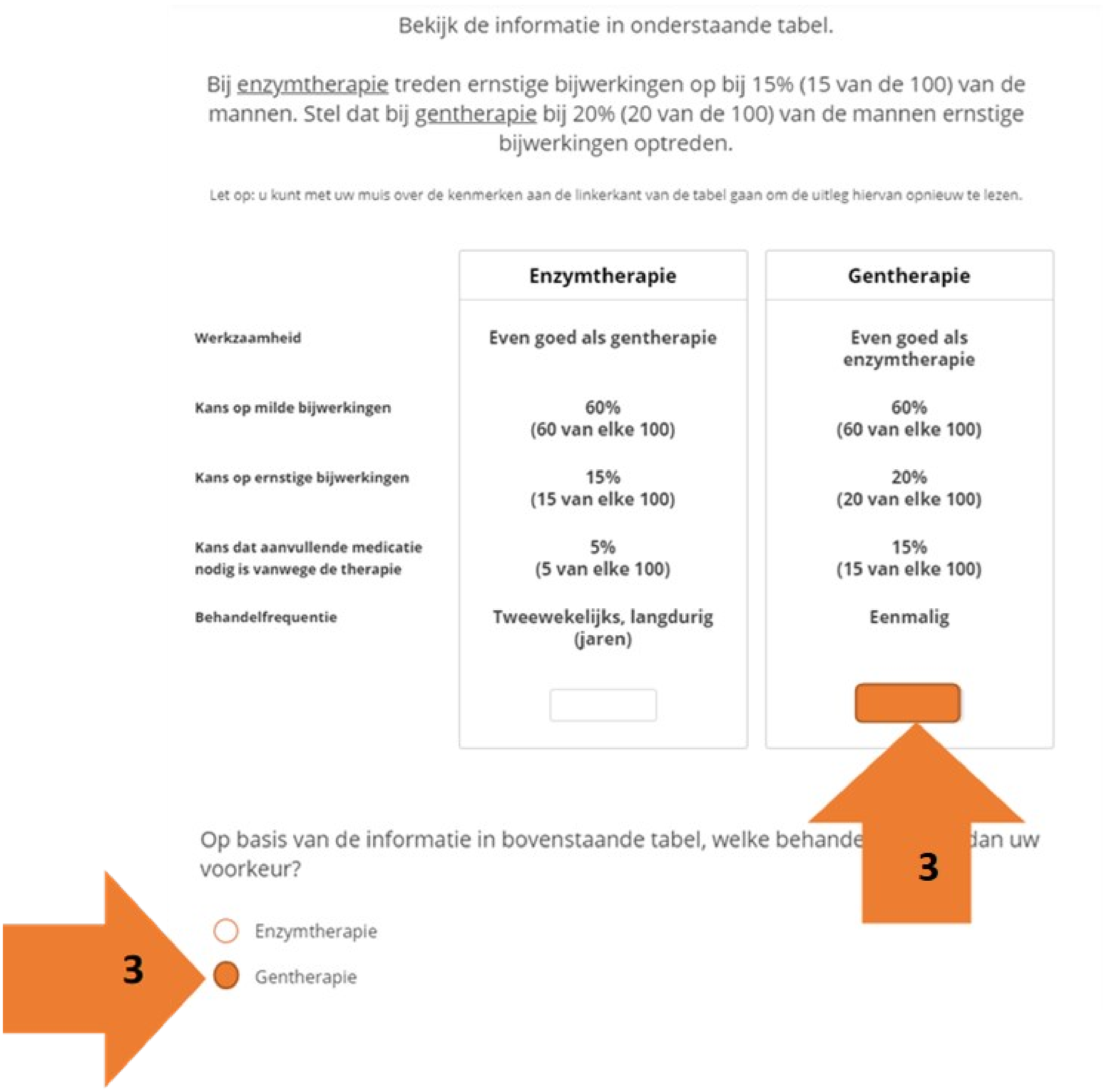

Bekijk de informatie in onderstaande tabel.

Bij enzymtherapie treden ernstige bijwerkingen op bij 15% (15 van de 100) van de mannen. Stel dat bij gentherapie bij 20% (20 van de 100) van de mannen ernstige bijwerkingen optreden.

Let op: u kunt met uw muis over de kenmerken aan de linkerkant van de tabel gaan om de uitleg hiervan opnieuw te lezen.

	Enzymtherapie	Gentherapie
Werkzaamheid	Even goed als gentherapie	Even goed als enzymtherapie
Kans op milde bijwerkingen	60% (60 van elke 100)	60% (60 van elke 100)
Kans op ernstige bijwerkingen	15% (15 van elke 100)	20% (20 van elke 100)
Kans dat aanvullende medicatie nodig is vanwege de therapie	5% (5 van elke 100)	15% (15 van elke 100)
Behandelfrequentie	Tweewekelijks, langdurig (jaren)	Eenmalig

Op basis van de informatie in bovenstaande tabel, welke behande[illegible]dan uw voorkeur?

- Enzymtherapie
- Gentherapie

Volgende

Deel 1: Milde bijwerkingen

Bij dit onderdeel van de vragenlijst gaat het om milde bijwerkingen die kunnen optreden bij het gebruik van enzymtherapie of gentherapie. Deze bijwerkingen duren kort, zijn zonder blijvende gevolgen en er is geen ziekenhuisopname nodig.

Volgende

Bekijk de informatie in onderstaande tabel.

Bij enzymtherapie treden milde bijwerkingen op bij 60% (60 van de 100) van de mannen. Stel dat ook bij gentherapie bij 60% (60 van de 100) van de mannen milde bijwerkingen optreden.

Let op: u kunt met uw muis over de kenmerken aan de linkerkant van de tabel gaan om de uitleg hiervan opnieuw te lezen.

	**Enzymtherapie**	**Gentherapie**
**Werkzaamheid**	**Even goed als gentherapie**	**Even goed als enzymtherapie**
**Kans op milde bijwerkingen**	**60% (60 van elke 100)**	**60% (60 van elke 100)**
**Kans op ernstige bijwerkingen**	**15% (15 van elke 100)**	**20% (20 van elke 100)**
**Kans dat aanvullende medicatie nodig is vanwege de therapie**	**5% (5 van elke 100)**	**15% (15 van elke 100)**
**Behandelfrequentie**	**Tweewekelijks, langdurig (jaren)**	**Eenmalig**

Op basis van de informatie in bovenstaande tabel, welke behandeling heeft dan uw voorkeur?

- ◯ Enzymtherapie
- ◯ Gentherapie

Volgende

Bekijk de informatie in onderstaande tabel.

Bij enzymtherapie treden milde bijwerkingen op bij 60% (60 van de 100) van de mannen. Stel dat bij gentherapie bij 40% (40 van de 100) van de mannen milde bijwerkingen optreden.

Let op: u kunt met uw muis over de kenmerken aan de linkerkant van de tabel gaan om de uitleg hiervan opnieuw te lezen.

	**Enzymtherapie**	**Gentherapie**
**Werkzaamheid**	**Even goed als gentherapie**	**Even goed als enzymtherapie**
**Kans op milde bijwerkingen**	**60% (60 van elke 100)**	**40% (40 van elke 100)**
**Kans op ernstige bijwerkingen**	**15% (15 van elke 100)**	**20% (20 van elke 100)**
**Kans dat aanvullende medicatie nodig is vanwege de therapie**	**5% (5 van elke 100)**	**15% (15 van elke 100)**
**Behandelfrequentie**	**Tweewekelijks, langdurig (jaren)**	**Eenmalig**

Op basis van de informatie in bovenstaande tabel, welke behandeling heeft dan uw voorkeur?

- ○ Enzymtherapie
- ○ Gentherapie

Volgende

Bekijk de informatie in onderstaande tabel.

Bij enzymtherapie treden milde bijwerkingen op bij 60% (60 van de 100) van de mannen. Stel dat bij gentherapie bij 50% (50 van de 100) van de mannen milde bijwerkingen optreden.

Let op: u kunt met uw muis over de kenmerken aan de linkerkant van de tabel gaan om de uitleg hiervan opnieuw te lezen.

	Enzymtherapie	Gentherapie
**Werkzaamheid**	**Even goed als gentherapie**	**Even goed als enzymtherapie**
**Kans op milde bijwerkingen**	**60% (60 van elke 100)**	**50% (50 van elke 100)**
**Kans op ernstige bijwerkingen**	**15% (15 van elke 100)**	**20% (20 van elke 100)**
**Kans dat aanvullende medicatie nodig is vanwege de therapie**	**5% (5 van elke 100)**	**15% (15 van elke 100)**
**Behandelfrequentie**	**Tweewekelijks, langdurig (jaren)**	**Eenmalig**

Op basis van de informatie in bovenstaande tabel, welke behandeling heeft dan uw voorkeur?

- Enzymtherapie
- Gentherapie

Volgende

Bekijk de informatie in onderstaande tabel.

Bij enzymtherapie treden milde bijwerkingen op bij 60% (60 van de 100) van de mannen. Stel dat bij gentherapie bij 80% (80 van de 100) van de mannen milde bijwerkingen optreden.

Let op: u kunt met uw muis over de kenmerken aan de linkerkant van de tabel gaan om de uitleg hiervan opnieuw te lezen.

	**Enzymtherapie**	**Gentherapie**
**Werkzaamheid**	**Even goed als gentherapie**	**Even goed als enzymtherapie**
**Kans op milde bijwerkingen**	**60%<br>(60 van elke 100)**	**80%<br>(80 van elke 100)**
**Kans op ernstige bijwerkingen**	**15%<br>(15 van elke 100)**	**20%<br>(20 van elke 100)**
**Kans dat aanvullende medicatie nodig is vanwege de therapie**	**5%<br>(5 van elke 100)**	**15%<br>(15 van elke 100)**
**Behandelfrequentie**	**Tweewekelijks, langdurig (jaren)**	**Eenmalig**

Op basis van de informatie in bovenstaande tabel, welke behandeling heeft dan uw voorkeur?

- ○ Enzymtherapie
- ○ Gentherapie

Volgende

Bekijk de informatie in onderstaande tabel.

Bij enzymtherapie treden milde bijwerkingen op bij 60% (60 van de 100) van de mannen. Stel dat bij gentherapie bij 70% (70 van de 100) van de mannen milde bijwerkingen optreden.

Let op: u kunt met uw muis over de kenmerken aan de linkerkant van de tabel gaan om de uitleg hiervan opnieuw te lezen.

	Enzymtherapie	Gentherapie
**Werkzaamheid**	**Even goed als gentherapie**	**Even goed als enzymtherapie**
**Kans op milde bijwerkingen**	**60% (60 van elke 100)**	**70% (70 van elke 100)**
**Kans op ernstige bijwerkingen**	**15% (15 van elke 100)**	**20% (20 van elke 100)**
**Kans dat aanvullende medicatie nodig is vanwege de therapie**	**5% (5 van elke 100)**	**15% (15 van elke 100)**
**Behandelfrequentie**	**Tweewekelijks, langdurig (jaren)**	**Eenmalig**

Op basis van de informatie in bovenstaande tabel, welke behandeling heeft dan uw voorkeur?

- ◯ Enzymtherapie
- ◯ Gentherapie

Volgende

U heeft net aangegeven dat u voor enzymtherapie zou kiezen als de kans op milde bijwerkingen bij gentherapie 40% is.

Wat is de hoogste kans (in %) op milde bijwerkingen waarbij u toch gentherapie zou kiezen? (let op: dit is dus altijd lager dan 40, maar kan ook 0 zijn indien u bijvoorbeeld helemaal geen gentherapie wil)

Volgende

U heeft net aangegeven dat u voor gentherapie zou kiezen als de kans op milde bijwerkingen 80% is.

Wat is de hoogste kans (in %) op milde bijwerkingen waarbij u nog gentherapie zou kiezen? (let op: dit is dus altijd hoger dan 80, maar kan ook 100 zijn indien u altijd voor gentherapie zou kiezen)

Volgende

Heeft u op dit moment of in het verleden een of meerdere van de **milde bijwerkingen** (gehad) die bij uw huidige therapie kunnen voorkomen?

- ◯ Ja
- ◯ Nee

Volgende

Deel 2: Ernstige bijwerkingen

Bij dit onderdeel van de vragenlijst gaat het om ernstige bijwerkingen die kunnen optreden bij het gebruik van enzymtherapie of bij gentherapie. Deze bijwerkingen hebben een ziekenhuisopname tot gevolg.

Volgende

Bekijk de informatie in onderstaande tabel.

Bij enzymtherapie treden ernstige bijwerkingen op bij 15% (15 van de 100) van de mannen. Stel dat bij gentherapie bij 20% (20 van de 100) van de mannen ernstige bijwerkingen optreden.

Let op: u kunt met uw muis over de kenmerken aan de linkerkant van de tabel gaan om de uitleg hiervan opnieuw te lezen.

	**Enzymtherapie**	**Gentherapie**
**Werkzaamheid**	**Even goed als gentherapie**	**Even goed als enzymtherapie**
**Kans op milde bijwerkingen**	**60% (60 van elke 100)**	**60% (60 van elke 100)**
**Kans op ernstige bijwerkingen**	**15% (15 van elke 100)**	**20% (20 van elke 100)**
**Kans dat aanvullende medicatie nodig is vanwege de therapie**	**5% (5 van elke 100)**	**15% (15 van elke 100)**
**Behandelfrequentie**	**Tweewekelijks, langdurig (jaren)**	**Eenmalig**

Op basis van de informatie in bovenstaande tabel, welke behandeling heeft dan uw voorkeur?

- ◯ Enzymtherapie
- ◯ Gentherapie

Volgende

Bekijk de informatie in onderstaande tabel.

Bij enzymtherapie treden ernstige bijwerkingen op bij 15% (15 van de 100) van de mannen. Stel dat bij gentherapie bij 5% (5 van de 100) van de mannen ernstige bijwerkingen optreden.

Let op: u kunt met uw muis over de kenmerken aan de linkerkant van de tabel gaan om de uitleg hiervan opnieuw te lezen.

	**Enzymtherapie**	**Gentherapie**
**Werkzaamheid**	**Even goed als gentherapie**	**Even goed als enzymtherapie**
**Kans op milde bijwerkingen**	**60% (60 van elke 100)**	**60% (60 van elke 100)**
**Kans op ernstige bijwerkingen**	**15% (15 van elke 100)**	**5% (5 van elke 100)**
**Kans dat aanvullende medicatie nodig is vanwege de therapie**	**5% (5 van elke 100)**	**15% (15 van elke 100)**
**Behandelfrequentie**	**Tweewekelijks, langdurig (jaren)**	**Eenmalig**

Op basis van de informatie in bovenstaande tabel, welke behandeling heeft dan uw voorkeur?

- Enzymtherapie
- Gentherapie

Volgende

Bekijk de informatie in onderstaande tabel.

Bij enzymtherapie treden ernstige bijwerkingen op bij 15% (15 van de 100) van de mannen. Stel dat bij gentherapie bij 10% (10 van de 100) van de mannen ernstige bijwerkingen optreden.

Let op: u kunt met uw muis over de kenmerken aan de linkerkant van de tabel gaan om de uitleg hiervan opnieuw te lezen.

	**Enzymtherapie**	**Gentherapie**
**Werkzaamheid**	**Even goed als gentherapie**	**Even goed als enzymtherapie**
**Kans op milde bijwerkingen**	**60% (60 van elke 100)**	**60% (60 van elke 100)**
**Kans op ernstige bijwerkingen**	**15% (15 van elke 100)**	**10% (10 van elke 100)**
**Kans dat aanvullende medicatie nodig is vanwege de therapie**	**5% (5 van elke 100)**	**15% (15 van elke 100)**
**Behandelfrequentie**	**Tweewekelijks, langdurig (jaren)**	**Eenmalig**

Op basis van de informatie in bovenstaande tabel, welke behandeling heeft dan uw voorkeur?

- ○ Enzymtherapie
- ○ Gentherapie

Volgende

Bekijk de informatie in onderstaande tabel.

Bij enzymtherapie treden ernstige bijwerkingen op bij 15% (15 van de 100) van de mannen. Stel dat bij gentherapie bij 80% (80 van de 100) van de mannen ernstige bijwerkingen optreden.

Let op: u kunt met uw muis over de kenmerken aan de linkerkant van de tabel gaan om de uitleg hiervan opnieuw te lezen.

	Enzymtherapie	Gentherapie
**Werkzaamheid**	**Even goed als gentherapie**	**Even goed als enzymtherapie**
**Kans op milde bijwerkingen**	**60% (60 van elke 100)**	**60% (60 van elke 100)**
**Kans op ernstige bijwerkingen**	**15% (15 van elke 100)**	**80% (80 van elke 100)**
**Kans dat aanvullende medicatie nodig is vanwege de therapie**	**5% (5 van elke 100)**	**15% (15 van elke 100)**
**Behandelfrequentie**	**Tweewekelijks, langdurig (jaren)**	**Eenmalig**

Op basis van de informatie in bovenstaande tabel, welke behandeling heeft dan uw voorkeur?

- ○ Enzymtherapie
- ○ Gentherapie

Volgende

Bekijk de informatie in onderstaande tabel.

Bij enzymtherapie treden ernstige bijwerkingen op bij 15% (15 van de 100) van de mannen. Stel dat bij gentherapie bij 50% (50 van de 100) van de mannen ernstige bijwerkingen optreden.

Let op: u kunt met uw muis over de kenmerken aan de linkerkant van de tabel gaan om de uitleg hiervan opnieuw te lezen.

	**Enzymtherapie**	**Gentherapie**
**Werkzaamheid**	**Even goed als gentherapie**	**Even goed als enzymtherapie**
**Kans op milde bijwerkingen**	**60% (60 van elke 100)**	**60% (60 van elke 100)**
**Kans op ernstige bijwerkingen**	**15 (15 van elke 100)**	**50% (50 van elke 100)**
**Kans dat aanvullende medicatie nodig is vanwege de therapie**	**5% (5 van elke 100)**	**15% (15 van elke 100)**
**Behandelfrequentie**	**Tweewekelijks, langdurig (jaren)**	**Eenmalig**

Op basis van de informatie in bovenstaande tabel, welke behandeling heeft dan uw voorkeur?

- ◯ Enzymtherapie
- ◯ Gentherapie

Volgende

U heeft net aangegeven dat u voor enzymtherapie zou kiezen als de kans op ernstige bijwerkingen bij gentherapie 5% is.

Wat is de hoogste kans (in %) op ernstige bijwerkingen waarbij u toch gentherapie zou kiezen? (let op: dit is dus altijd lager dan 5, maar kan ook 0 zijn indien u bijvoorbeeld helemaal geen gentherapie wil)

Volgende

U heeft net aangegeven dat u voor gentherapie zou kiezen als de kans op ernstige bijwerkingen 80% is.

Wat is de hoogste kans (in %) op ernstige bijwerkingen waarbij u nog gentherapie zou kiezen? (let op: dit is dus altijd hoger dan 80, maar kan ook 100 zijn indien u altijd voor gentherapie zou kiezen)

Volgende

Heeft u op dit moment of in het verleden een of meerdere van de **ernstige bijwerkingen** (gehad) die bij uw huidige therapie kunnen voorkomen?

- ◯ Ja
- ◯ Nee

Volgende

Deel 3: gebruik van aanvullende medicatie

Bij dit onderdeel van de vragenlijst gaat het om het gebruik van aanvullende medicatie, wat nodig kan zijn om de bijwerkingen van enzymtherapie of gentherapie te onderdrukken. Dit kan zowel kortdurend als langdurig zijn.

Volgende

Bekijk de informatie in onderstaande tabel.

Bij enzymtherapie is het gebruik van aanvullende medicatie nodig bij 5% (5 van de 100) van de mannen. Stel dat bij gentherapie bij 15% (15 van de 100) van de mannen aanvullende medicatie nodig zou zijn.

Let op: u kunt met uw muis over de kenmerken aan de linkerkant van de tabel gaan om de uitleg hiervan opnieuw te lezen.

	**Enzymtherapie**	**Gentherapie**
**Werkzaamheid**	**Even goed als gentherapie**	**Even goed als enzymtherapie**
**Kans op milde bijwerkingen**	**60% (60 van elke 100)**	**60% (60 van elke 100)**
**Kans op ernstige bijwerkingen**	**15% (15 van elke 100)**	**20% (20 van elke 100)**
**Kans dat aanvullende medicatie nodig is vanwege de therapie**	**5% (5 van elke 100)**	**15% (15 van elke 100)**
**Behandelfrequentie**	**Tweewekelijks, langdurig (jaren)**	**Eenmalig**

Op basis van de informatie in bovenstaande tabel, welke behandeling heeft dan uw voorkeur?

- ◯ Enzymtherapie
- ◯ Gentherapie

Volgende

Bekijk de informatie in onderstaande tabel.

Bij enzymtherapie is het gebruik van aanvullende medicatie nodig bij 5% (5 van de 100) van de mannen. Stel dat bij gentherapie bij 5% (5 van de 100) van de mannen aanvullende medicatie nodig zou zijn.

Let op: u kunt met uw muis over de kenmerken aan de linkerkant van de tabel gaan om de uitleg hiervan opnieuw te lezen.

	**Enzymtherapie**	**Gentherapie**
**Werkzaamheid**	**Even goed als gentherapie**	**Even goed als enzymtherapie**
**Kans op milde bijwerkingen**	**60% (60 van elke 100)**	**60% (60 van elke 100)**
**Kans op ernstige bijwerkingen**	**15% (15 van elke 100)**	**20% (20 van elke 100)**
**Kans dat aanvullende medicatie nodig is vanwege de therapie**	**5% (5 van elke 100)**	**5% (5 van elke 100)**
**Behandelfrequentie**	**Tweewekelijks, langdurig (jaren)**	**Eenmalig**

Op basis van de informatie in bovenstaande tabel, welke behandeling heeft dan uw voorkeur?

- ○ Enzymtherapie
- ○ Gentherapie

Volgende

Bekijk de informatie in onderstaande tabel.

Bij enzymtherapie is het gebruik van aanvullende medicatie nodig bij 5% (5 van de 100) van de mannen. Stel dat bij gentherapie bij 10% (10 van de 100) van de mannen aanvullende medicatie nodig zou zijn.

Let op: u kunt met uw muis over de kenmerken aan de linkerkant van de tabel gaan om de uitleg hiervan opnieuw te lezen.

	**Enzymtherapie**	**Gentherapie**
**Werkzaamheid**	**Even goed als gentherapie**	**Even goed als enzymtherapie**
**Kans op milde bijwerkingen**	**60%<br>(60 van elke 100)**	**60%<br>(60 van elke 100)**
**Kans op ernstige bijwerkingen**	**15%<br>(15 van elke 100)**	**20%<br>(20 van elke 100)**
**Kans dat aanvullende medicatie nodig is vanwege de therapie**	**5%<br>(5 van elke 100)**	**10%<br>(10 van elke 100)**
**Behandelfrequentie**	**Tweewekelijks, langdurig (jaren)**	**Eenmalig**

Op basis van de informatie in bovenstaande tabel, welke behandeling heeft dan uw voorkeur?

- ◯ Enzymtherapie
- ◯ Gentherapie

Volgende

Bekijk de informatie in onderstaande tabel.

Bij enzymtherapie is het gebruik van aanvullende medicatie nodig bij 5% (5 van de 100) van de mannen. Stel dat bij gentherapie bij 50% (50 van de 100) van de mannen aanvullende medicatie nodig zou zijn.

Let op: u kunt met uw muis over de kenmerken aan de linkerkant van de tabel gaan om de uitleg hiervan opnieuw te lezen.

	**Enzymtherapie**	**Gentherapie**
**Werkzaamheid**	**Even goed als gentherapie**	**Even goed als enzymtherapie**
**Kans op milde bijwerkingen**	**60% (60 van elke 100)**	**60% (60 van elke 100)**
**Kans op ernstige bijwerkingen**	**15% (15 van elke 100)**	**20% (20 van elke 100)**
**Kans dat aanvullende medicatie nodig is vanwege de therapie**	**5% (5 van elke 100)**	**50% (50 van elke 100)**
**Behandelfrequentie**	**Tweewekelijks, langdurig (jaren)**	**Eenmalig**

Op basis van de informatie in bovenstaande tabel, welke behandeling heeft dan uw voorkeur?

- ◯ Enzymtherapie
- ◯ Gentherapie

Volgende

Bekijk de informatie in onderstaande tabel.

Bij enzymtherapie is het gebruik van aanvullende medicatie nodig bij 5% (5 van de 100) van de mannen. Stel dat bij gentherapie bij 30% (30 van de 100) van de mannen aanvullende medicatie nodig zou zijn.

Let op: u kunt met uw muis over de kenmerken aan de linkerkant van de tabel gaan om de uitleg hiervan opnieuw te lezen.

	**Enzymtherapie**	**Gentherapie**
**Werkzaamheid**	**Even goed als gentherapie**	**Even goed als enzymtherapie**
**Kans op milde bijwerkingen**	**60% (60 van elke 100)**	**60% (60 van elke 100)**
**Kans op ernstige bijwerkingen**	**15% (15 van elke 100)**	**20% (20 van elke 100)**
**Kans dat aanvullende medicatie nodig is vanwege de therapie**	**5% (5 van elke 100)**	**30% (30 van elke 100)**
**Behandelfrequentie**	**Tweewekelijks, langdurig (jaren)**	**Eenmalig**

Op basis van de informatie in bovenstaande tabel, welke behandeling heeft dan uw voorkeur?

- ◯ Enzymtherapie
- ◯ Gentherapie

Volgende

U heeft net aangegeven dat u voor enzymtherapie zou kiezen als de kans op aanvullende medicatie bij gentherapie 5% is.

Wat is de hoogste kans (in %) op aanvullende medicatie waarbij u toch gentherapie zou kiezen? (let op: dit is dus altijd lager dan 5, maar kan ook 0 zijn indien u bijvoorbeeld helemaal geen gentherapie wil)

Volgende

U heeft net aangegeven dat u voor gentherapie zou kiezen als de kans op aanvullende medicatie 50% is.

Wat is de hoogste kans (in %) op aanvullende medicatie waarbij u nog gentherapie zou kiezen? (let op: dit is dus altijd hoger dan 50, maar kan ook 100 zijn indien u altijd voor gentherapie zou kiezen)

Volgende

Gebruikt(e) u op dit moment of in het verleden een of meerdere aanvullende medicijnen die door uw therapie nodig zijn?

- ◯ Ja
- ◯ Nee

Volgende

Deel 4: onzekerheidsvraag

Bij de keuze om wel of niet mee te doen aan een studie naar een nieuw geneesmiddel kan ook de onzekerheid over werkzaamheid en bijwerkingen meespelen. Er moet dan gekozen worden tussen therapie waarvan de werkzaamheid (gedeeltelijk) bekend is (enzymtherapie) en een nieuwe therapie in studieverband met meer onzekerheden (gentherapie). Deze vraag gaat over die onzekerheid.

We gaan er in dit scenario van uit dat gentherapie – in ieder geval op korte termijn (tot ca. 2 jaar) - even goed werkt als uw huidige therapie.

Enzymtherapie is voldoende veilig bevonden om op recept voorgeschreven te mogen worden. Voor gentherapie is dat nog niet het geval, dus het wordt alleen nog in studieverband gegeven. Of het veilig en werkzaam is moet nog worden vastgesteld.

Voor de ziekte van Fabry kan pas na jaren worden geconcludeerd of een nieuwe therapie goed werkt omdat de ziekte zich langzaam ontwikkelt.

Volgende

Bekijk de informatie in onderstaande tabel.

Enzymtherapie is voldoende veilig bevonden om op recept voorgeschreven te mogen worden. Stel dat voor gentherapie de kans dat dit veilig en werkzaam blijkt 25% is.

Let op: u kunt met uw muis over de kenmerken aan de linkerkant van de tabel gaan om de uitleg hiervan opnieuw te lezen.

	**Enzymtherapie**	**Gentherapie**
**Werkzaamheid op korte termijn (ca. 2 jaar)**	**Even goed als gentherapie**	**Even goed als enzymtherapie**
**Kans dat het middel op langere termijn als veilig en effectief wordt beschouwd**	**75-100%***	**25%**
**Kans op milde bijwerkingen**	**60%<br>(60 van elke 100)**	**60%<br>(60 van elke 100)**
**Kans op ernstige bijwerkingen**	**15%<br>(15 van elke 100)**	**20%<br>(20 van elke 100)**
**Kans dat aanvullende medicatie nodig is vanwege de therapie**	**5%<br>(5 van elke 100)**	**15%<br>(15 van elke 100)**
**Behandelfrequentie**	**Tweewekelijks, langdurig (jaren)**	**Eenmalig**

*Enzymtherapie is sinds ongeveer 20 jaar beschikbaar en is veilig gebleken. Door de zeldzaamheid en het trage beloop van de ziekte van Fabry leren we nog steeds voor welke patiënten en op welk moment enzymtherapie werkzaam is. Daarom kan dit percentage per persoon iets verschillen.

Op basis van de informatie in bovenstaande tabel, welke behandeling heeft dan uw voorkeur?

- ◯ Enzymtherapie
- ◯ Gentherapie

Volgende

Bekijk de informatie in onderstaande tabel.

Enzymtherapie is voldoende veilig bevonden om op recept voorgeschreven te mogen worden. Stel dat voor gentherapie de kans dat dit veilig en werkzaam blijkt 5% is.

Let op: u kunt met uw muis over de kenmerken aan de linkerkant van de tabel gaan om de uitleg hiervan opnieuw te lezen.

	**Enzymtherapie**	**Gentherapie**
**Werkzaamheid op korte termijn (ca. 2 jaar)**	**Even goed als gentherapie**	**Even goed als enzymtherapie**
**Kans dat het middel op langere termijn als veilig en effectief wordt beschouwd**	**75%-100%***	**5%**
**Kans op milde bijwerkingen**	**60% (60 van elke 100)**	**60% (60 van elke 100)**
**Kans op ernstige bijwerkingen**	**15% (15 van elke 100)**	**20% (20 van elke 100)**
**Kans dat aanvullende medicatie nodig is vanwege de therapie**	**5% (5 van elke 100)**	**15% (15 van elke 100)**
**Behandelfrequentie**	**Tweewekelijks, langdurig (jaren)**	**Eenmalig**

*Enzymtherapie is sinds ongeveer 20 jaar beschikbaar en is veilig gebleken. Door de zeldzaamheid en het trage beloop van de ziekte van Fabry leren we nog steeds voor welke patiënten en op welk moment enzymtherapie werkzaam is. Daarom kan dit percentage per persoon iets verschillen.

Op basis van de informatie in bovenstaande tabel, welke behandeling heeft dan uw voorkeur?

- ◯ Enzymtherapie
- ◯ Gentherapie

Volgende

Bekijk de informatie in onderstaande tabel.

Enzymtherapie is voldoende veilig bevonden om op recept voorgeschreven te mogen worden. Stel dat voor gentherapie de kans dat dit veilig en werkzaam blijkt 10% is.

Let op: u kunt met uw muis over de kenmerken aan de linkerkant van de tabel gaan om de uitleg hiervan opnieuw te lezen.

	**Enzymtherapie**	**Gentherapie**
**Werkzaamheid op korte termijn (ca. 2 jaar))**	**Even goed als gentherapie**	**Even goed als enzymtherapie**
**Kans dat het middel op langere termijn als veilig en effectief wordt beschouwd**	**75%-100%***	**10%**
**Kans op milde bijwerkingen**	**60% (60 van elke 100)**	**60% (60 van elke 100)**
**Kans op ernstige bijwerkingen**	**15% (15 van elke 100)**	**20% (20 van elke 100)**
**Kans dat aanvullende medicatie nodig is vanwege de therapie**	**5% (5 van elke 100)**	**15% (15 van elke 100)**
**Behandelfrequentie**	**Tweewekelijks, langdurig (jaren)**	**Eenmalig**

*Enzymtherapie is sinds ongeveer 20 jaar beschikbaar en is veilig gebleken. Door de zeldzaamheid en het trage beloop van de ziekte van Fabry leren we nog steeds voor welke patiënten en op welk moment enzymtherapie werkzaam is. Daarom kan dit percentage per persoon iets verschillen.

Op basis van de informatie in bovenstaande tabel, welke behandeling heeft dan uw voorkeur?

- ○ Enzymtherapie
- ○ Gentherapie

Volgende

Bekijk de informatie in onderstaande tabel.

Enzymtherapie is voldoende veilig bevonden om op recept voorgeschreven te mogen worden. Stel dat voor gentherapie de kans dat dit veilig en werkzaam blijkt 50% is.

Let op: u kunt met uw muis over de kenmerken aan de linkerkant van de tabel gaan om de uitleg hiervan opnieuw te lezen.

	**Enzymtherapie**	**Gentherapie**
**Werkzaamheid op korte termijn (ca. 2 jaar))**	**Even goed als gentherapie**	**Even goed als enzymtherapie**
**Kans dat het middel op langere termijn als veilig en effectief wordt geschouwd**	**75%-100%**	**50%**
**Kans op milde bijwerkingen**	**60% (60 van elke 100)**	**60% (60 van elke 100)**
**Kans op ernstige bijwerkingen**	**15% (15 van elke 100)**	**20% (20 van elke 100)**
**Kans dat aanvullende medicatie nodig is vanwege de therapie**	**5% (5 van elke 100)**	**15% (15 van elke 100)**
**Behandelfrequentie**	**Tweewekelijks, langdurig (jaren)**	**Eenmalig**

*Enzymtherapie is sinds ongeveer 20 jaar beschikbaar en is veilig gebleken. Door de zeldzaamheid en het trage beloop van de ziekte van Fabry leren we nog steeds voor welke patiënten en op welk moment enzymtherapie werkzaam is. Daarom kan dit percentage per persoon iets verschillen.

Op basis van de informatie in bovenstaande tabel, welke behandeling heeft dan uw voorkeur?

- ○ Enzymtherapie
- ○ Gentherapie

Volgende

Bekijk de informatie in onderstaande tabel.

Enzymtherapie is voldoende veilig bevonden om op recept voorgeschreven te mogen worden. Stel dat voor gentherapie de kans dat dit veilig en werkzaam blijkt 30% is.

Let op: u kunt met uw muis over de kenmerken aan de linkerkant van de tabel gaan om de uitleg hiervan opnieuw te lezen.

	**Enzymtherapie**	**Gentherapie**
**Werkzaamheid op korte termijn (ca. 2 jaar))**	Even goed als gentherapie	Even goed als enzymtherapie
**Kans dat het middel op langere termijn als veilig en effectief wordt beschouwd**	75%-100%*	30%
**Kans op milde bijwerkingen**	60% (60 van elke 100)	
**Kans op ernstige bijwerkingen**	15% (15 van elke 100)	
**Kans dat aanvullende medicatie nodig is vanwege de therapie**	5% (5 van elke 100)	
**Behandelfrequentie**	Tweewekelijks, langdurig (jaren)	

**60% (60 van elke 100)**
**20% (20 van elke 100)**
**15% (15 van elke 100)**
**Eenmalig**

*Enzymtherapie is sinds ongeveer 20 jaar beschikbaar en is veilig gebleken. Door de zeldzaamheid en het trage beloop van de ziekte van Fabry leren we nog steeds voor welke patiënten en op welk moment enzymtherapie werkzaam is. Daarom kan dit percentage per persoon iets verschillen.

Op basis van de informatie in bovenstaande tabel, welke behandeling heeft dan uw voorkeur?

- ◯ Enzymtherapie
- ◯ Gentherapie

Volgende

U heeft net aangegeven dat u voor gentherapie zou kiezen als de kans dat gentherapie in de komende jaren als veilig en effectief wordt beschouwd 5% is.

Wat is de minimale kans (in %) dat het middel in de komende jaren als veilig en effectief wordt beschouwd waarbij u nog voor gentherapie zou kiezen? (let op: dit is dus altijd lager dan 5, maar kan ook 0 zijn indien u bijvoorbeeld helemaal geen gentherapie wil)

Volgende

U heeft net aangegeven dat u voor enzymtherapie zou kiezen als de kans dat gentherapie in de komende jaren als veilig en effectief wordt beschouwd 50% is.

Wat is de minimale kans (in %) dat het middel in de komende jaren als veilig en effectief wordt beschouwd waarbij u toch voor gentherapie zou kiezen? (let op: dit is dus altijd hoger dan 50, maar kan ook 100 zijn indien u altijd voor gentherapie zou kiezen)

Volgende

Deel 5: Stel gentherapie werkt beter

We gaan er in dit scenario van uit dat gentherapie **een beter effect** heeft dan enzymtherapie. Of gentherapie veilig en werkzaam is, moet uitgezocht worden in studieverband.

Volgende

Bekijk de informatie in onderstaande tabel.

Enzymtherapie is voldoende veilig bevonden om op recept voorgeschreven te mogen worden. Stel dat voor gentherapie de kans dat dit veilig en werkzaam blijkt 25% is.

Let op: u kunt met uw muis over de kenmerken aan de rechterkant van de tabel gaan om de uitleg hiervan opnieuw te lezen.

	Enzymtherapie	Gentherapie
**Werkzaamheid op korte termijn (ca. 2 jaar)**	**Minder goed dan gentherapie**	**Beter dan enzymtherapie**
**Kans dat het middel op langere termijn als veilig en effectief wordt beschouwd**	**75%-100%***	**25%**
**Kans op milde bijwerkingen**	**60% (60 van elke 100)**	**60% (60 van elke 100)**
**Kans op ernstige bijwerkingen**	**15% (15 van elke 100)**	**20% (20 van elke 100)**
**Kans dat aanvullende medicatie nodig is vanwege de therapie**	**5% (5 van elke 100)**	**15% (15 van elke 100)**
**Behandelfrequentie**	**Tweewekelijks, langdurig (jaren)**	**Eenmalig**

*Enzymtherapie is sinds ongeveer 20 jaar beschikbaar en is veilig gebleken. Door de zeldzaamheid en het trage beloop van de ziekte van Fabry leren we nog steeds voor welke patiënten en op welk moment enzymtherapie werkzaam is. Daarom kan dit percentage per persoon iets verschillen.

Op basis van de informatie in bovenstaande tabel, welke behandeling heeft dan uw voorkeur?

- ◯ Enzymtherapie
- ◯ Gentherapie

Volgende

Bekijk de informatie in onderstaande tabel.

Enzymtherapie is voldoende veilig bevonden om op recept voorgeschreven te mogen worden. Stel dat voor gentherapie de kans dat dit veilig en werkzaam blijkt 5% is.

Let op: u kunt met uw muis over de kenmerken aan de linkerkant van de tabel gaan om de uitleg hiervan opnieuw te lezen.

	Enzymtherapie	Gentherapie
**Werkzaamheid op korte termijn (ca. 2 jaar)**	**Minder goed dan gentherapie**	**Beter dan enzymtherapie**
**Kans dat het middel op langere termijn als veilig en effectief wordt beschouwd**	**75%-100%***	**5%**
**Kans op milde bijwerkingen**	**60% (60 van elke 100)**	**60% (60 van elke 100)**
**Kans op ernstige bijwerkingen**	**15% (15 van elke 100)**	**20% (20 van elke 100)**
**Kans dat aanvullende medicatie nodig is vanwege de therapie**	**5% (5 van elke 100)**	**15% (15 van elke 100)**
**Behandelfrequentie**	**Tweewekelijks, langdurig (jaren)**	**Eenmalig**

*Enzymtherapie is sinds ongeveer 20 jaar beschikbaar en is veilig gebleken. Door de zeldzaamheid en het trage beloop van de ziekte van Fabry leren we nog steeds voor welke patiënten en op welk moment enzymtherapie werkzaam is. Daarom kan dit percentage per persoon iets verschillen.

Op basis van de informatie in bovenstaande tabel, welke behandeling heeft dan uw voorkeur?

- ○ Enzymtherapie
- ○ Gentherapie

Volgende

Bekijk de informatie in onderstaande tabel.

Enzymtherapie is voldoende veilig bevonden om op recept voorgeschreven te mogen worden. Stel dat voor gentherapie de kans dat dit veilig en werkzaam blijkt 10% is.

Let op: u kunt met uw muis over de kenmerken aan de linkerkant van de tabel gaan om de uitleg hiervan opnieuw te lezen.

	Enzymtherapie	Gentherapie
**Werkzaamheid op korte termijn (ca. 2 jaar)**	**Minder goed dan gentherapie**	**Beter dan enzymtherapie**
**Kans dat het middel op langere termijn als veilig en effectief wordt beschouwd**	**75%-100%***	**10%**
**Kans op milde bijwerkingen**	**60%<br>(60 van elke 100)**	**60%<br>(60 van elke 100)**
**Kans op ernstige bijwerkingen**	**15%<br>(15 van elke 100)**	**20%<br>(20 van elke 100)**
**Kans dat aanvullende medicatie nodig is vanwege de therapie**	**5%<br>(5 van elke 100)**	**15%<br>(15 van elke 100)**
**Behandelfrequentie**	**Tweewekelijks, langdurig (jaren)**	**Eenmalig**

*Enzymtherapie is sinds ongeveer 20 jaar beschikbaar en is veilig gebleken. Door de zeldzaamheid en het trage beloop van de ziekte van Fabry leren we nog steeds voor welke patiënten en op welk moment enzymtherapie werkzaam is. Daarom kan dit percentage per persoon iets verschillen.

Op basis van de informatie in bovenstaande tabel, welke behandeling heeft dan uw voorkeur?

- ○ Enzymtherapie
- ○ Gentherapie

Volgende

Bekijk de informatie in onderstaande tabel.

Enzymtherapie is voldoende veilig bevonden om op recept voorgeschreven te mogen worden. Stel dat voor gentherapie de kans dat dit veilig en werkzaam blijkt 50% is.

Let op: u kunt met uw muis over de kenmerken aan de linkerkant van de tabel gaan om de uitleg hiervan opnieuw te lezen.

	**Enzymtherapie**	**Gentherapie**
**Werkzaamheid op korte termijn (ca. 2 jaar)**	**Minder goed dan gentherapie**	**Beter dan enzymtherapie**
**Kans dat het middel op langere termijn als veilig en effectief wordt beschouwd**	**75%-100%***	**50%**
**Kans op milde bijwerkingen**	**60% (60 van elke 100)**	**60% (60 van elke 100)**
**Kans op ernstige bijwerkingen**	**15% (15 van elke 100)**	**20% (20 van elke 100)**
**Kans dat aanvullende medicatie nodig is vanwege de therapie**	**5% (5 van elke 100)**	**15% (15 van elke 100)**
**Behandelfrequentie**	**Tweewekelijks, langdurig (jaren)**	**Eenmalig**

*Enzymtherapie is sinds ongeveer 20 jaar beschikbaar en is veilig gebleken. Door de zeldzaamheid en het trage beloop van de ziekte van Fabry leren we nog steeds voor welke patiënten en op welk moment enzymtherapie werkzaam is. Daarom kan dit percentage per persoon iets verschillen.

Op basis van de informatie in bovenstaande tabel, welke behandeling heeft dan uw voorkeur?

- Enzymtherapie
- Gentherapie

Volgende

Bekijk de informatie in onderstaande tabel.

Enzymtherapie is voldoende veilig bevonden om op recept voorgeschreven te mogen worden. Stel dat voor gentherapie de kans dat dit veilig en werkzaam blijkt 30% is.

Let op: u kunt met uw muis over de kenmerken aan de linkerkant van de tabel gaan om de uitleg hiervan opnieuw te lezen.

	**Enzymtherapie**	**Gentherapie**
**Werkzaamheid op korte termijn (ca. 2 jaar)**	**Minder goed dan gentherapie**	**Beter dan enzymtherapie**
**Kans dat het middel op langere termijn als veilig en effectief wordt beschouwd**	**75%-100%***	**30%**
**Kans op milde bijwerkingen**	**60% (60 van elke 100)**	**60% (60 van elke 100)**
**Kans op ernstige bijwerkingen**	**15% (15 van elke 100)**	**20% (20 van elke 100)**
**Kans dat aanvullende medicatie nodig is vanwege de therapie**	**5% (5 van elke 100)**	**15% (15 van elke 100)**
**Behandelfrequentie**	**Tweewekelijks, langdurig (jaren)**	**Eenmalig**

*Enzymtherapie is sinds ongeveer 20 jaar beschikbaar en is veilig gebleken. Door de zeldzaamheid en het trage beloop van de ziekte van Fabry leren we nog steeds voor welke patiënten en op welk moment enzymtherapie werkzaam is. Daarom kan dit percentage per persoon iets verschillen.

Op basis van de informatie in bovenstaande tabel, welke behandeling heeft dan uw voorkeur?

- ○ Enzymtherapie
- ○ Gentherapie

Volgende

U heeft net aangegeven dat u voor gentherapie zou kiezen als de kans dat gentherapie in de komende jaren als veilig en effectief wordt beschouwd 5% is.

Wat is de minimale kans (in %) dat het middel in de komende jaren als veilig en effectief wordt beschouwd waarbij u nog voor gentherapie zou kiezen? (let op: dit is dus altijd lager dan 5, maar kan ook 0 zijn indien u bijvoorbeeld helemaal geen gentherapie wil)

Volgende

U heeft net aangegeven dat u voor enzymtherapie zou kiezen als de kans dat gentherapie in de komende jaren als veilig en effectief wordt beschouwd 50% is.

Wat is de minimale kans (in %) dat het middel in de komende jaren als veilig en effectief wordt beschouwd waarbij u toch voor gentherapie zou kiezen? (let op: dit is dus altijd hoger dan 50, maar kan ook 100 zijn indien u altijd voor gentherapie zou kiezen)

Volgende

Heeft u ooit aan een studie naar een nieuwe (vorm van) therapie voor de ziekte van Fabry meegewerkt? Studies waar u op dit moment nog aan meewerkt tellen ook mee.

- ◯ Ja
- ◯ Nee

Volgende

Uw mening over de medicijnen die u gebruikt

Als laatste onderdeel van deze vragenlijst willen we u vragen om per vraag het antwoord te kiezen dat het beste uw mening weergeeft over medicatie die u voorgeschreven heeft gekregen of nog krijgt. Het gaat hierbij specifiek om medicatie die u krijgt vanwege de ziekte van Fabry, zoals enzymtherapie en chaperone therapie, maar ook om pijnstilling of middelen voor uw bloeddruk, hart of nieren. We vragen u om medicijnen die u eventueel in het kader van andere aandoeningen voorgeschreven krijgt hier niet mee te wegen in uw keuze.

U kunt per vraag een antwoord kiezen.

	Helemaal niet mee eens	Niet mee eens	Geen duidelijke mening	Mee eens	Helemaal mee eens
Op het moment hangt mijn gezondheid af van mijn medicijnen	◯	◯	◯	◯	◯
Ik maak met zorgen over het feit dat ik medicijnen moet nemen	◯	◯	◯	◯	◯
Mijn leven zou erg moeilijk zijn zonder medicijnen	◯	◯	◯	◯	◯
Soms maak ik me zorgen over de effecten die mijn medicijnen op de langere termijn kunnen hebben	◯	◯	◯	◯	◯
Zonder mijn medicijnen zou ik heel ziek zijn	◯	◯	◯	◯	◯
Ik ben onvoldoende op de hoogte van wat mijn medicijnen doen	◯	◯	◯	◯	◯
Mijn toekomstige gezondheid hangt af van mijn medicijnen	◯	◯	◯	◯	◯
Mijn medicijnen ontwrichten mijn leven	◯	◯	◯	◯	◯
Soms ben ik bang dat ik te afhankelijk zal worden van mijn medicijnen	◯	◯	◯	◯	◯
Mijn medicijnen voorkomen dat ik verder achteruit ga	◯	◯	◯	◯	◯
Deze medicijnen hebben onplezierige bijwerkingen	◯	◯	◯	◯	◯

Volgende

Afronding vragenlijst & contact

U bent bij het einde van de vragenlijst aangekomen. We willen u van harte bedanken voor uw tijd en het beantwoorden van de vragen. Een samenvatting van onze bevindingen uit dit onderzoek zal na het verwerken van de resultaten met alle deelnemers van dit vragenlijstonderzoek via email worden gedeeld.

Heeft u nog vragen of opmerkingen over de vragenlijst, dit onderzoek of het onderwerp?

Volgende

U gaf aan dat u Migalastat (Galafold©) gebruikt. Om de vragenlijst verder in te vullen en deel te nemen aan dit onderzoek vragen we u vriendelijk contact op te nemen met een van de onderzoekers, Ellie Corazolla (e.m.corazolla@amsterdamumc.nl).

Nogmaals hartelijk dank voor uw medewerking.

Als u aanvullende achtergrond informatie wenst over gentherapie voor stofwisselingsziekten willen we u wijzen op deze uitgebreide informatievideo van patientenvereniging VKS. Klik hier om deze video te bekijken.

PTT survey for participants with a child with MPS III

Volgende

Doel van dit onderzoek

Uit groepsgesprekken en interviews met (ouders van) patiënten met MPS III is gebleken dat er verschillende factoren zijn die maken of patiënten in de toekomst gentherapie zouden willen ontvangen. De belangrijkste factoren zijn eventuele bijwerkingen van therapie en de te verwachten werkzaamheid (effectiviteit).

Met deze vragenlijst willen wij – samen met VKS, de patiëntenvereniging voor patiënten met een erfelijke stofwisselingsziekte – onderzoeken wanneer ouders van patiënten hun kind eventueel gentherapie zouden willen laten ontvangen als ze hiervoor (in studieverband) in aanmerking zouden komen.

Om te kunnen onderzoeken in hoeverre deze factoren invloed hebben op de keuzes van ouders van patiënten stellen we u vragen over:
- de huidige situatie van uw kind
- gentherapie
- geneesmiddelen in het algemeen

Wij vragen u om aan te geven of u onder verschillende omstandigheden zou kiezen voor behandeling met gentherapie voor uw kind. Voor deze vragenlijst gaan we ervan uit dat uw kind behandeld kan worden met gentherapie. Of dit in de toekomst daadwerkelijk zo zal zijn is niet bekend.

U kunt de vragenlijst op elk moment sluiten en later op dezelfde plek verdergaan met invullen. Tijdens het invullen van de vragenlijst kunt u niet terug naar vorige vragen. Als u bij het invullen van de vragenlijst hulp wilt of tegen iets aanloopt, sluit de vragenlijst en stuur een mail naar een van de onderzoekers, Ellie Corazolla (e.m.corazolla@amsterdamumc.nl), met de volgende informatie:
- Waar loopt u tegenaan?
- Wilt u via de mail of telefonisch geholpen worden?
- Als u telefonisch contact wenst: Wanneer bent u in de komende dagen bereikbaar? En op welk telefoonnummer?

Amsterdam UMC
Universitair Medische Centra

Volgende

Achtergrondinformatie

Bij mucopolysaccharidose type III (MPS III) zit er een fout in het erfelijk (genetisch) materiaal (DNA) waardoor een bepaald eiwit (ook wel enzym) niet of niet goed werkt. Daardoor stapelt er een vetachtige stof in de cellen.

Gentherapie is een vorm van therapie waarbij 'nieuw' erfelijk materiaal in cellen wordt ingebracht. Het doel hiervan is om een stukje DNA toe te voegen zodat er een goed werkend enzym gevormd kan worden. Grofweg zijn er twee soorten gentherapie (zie figuur).

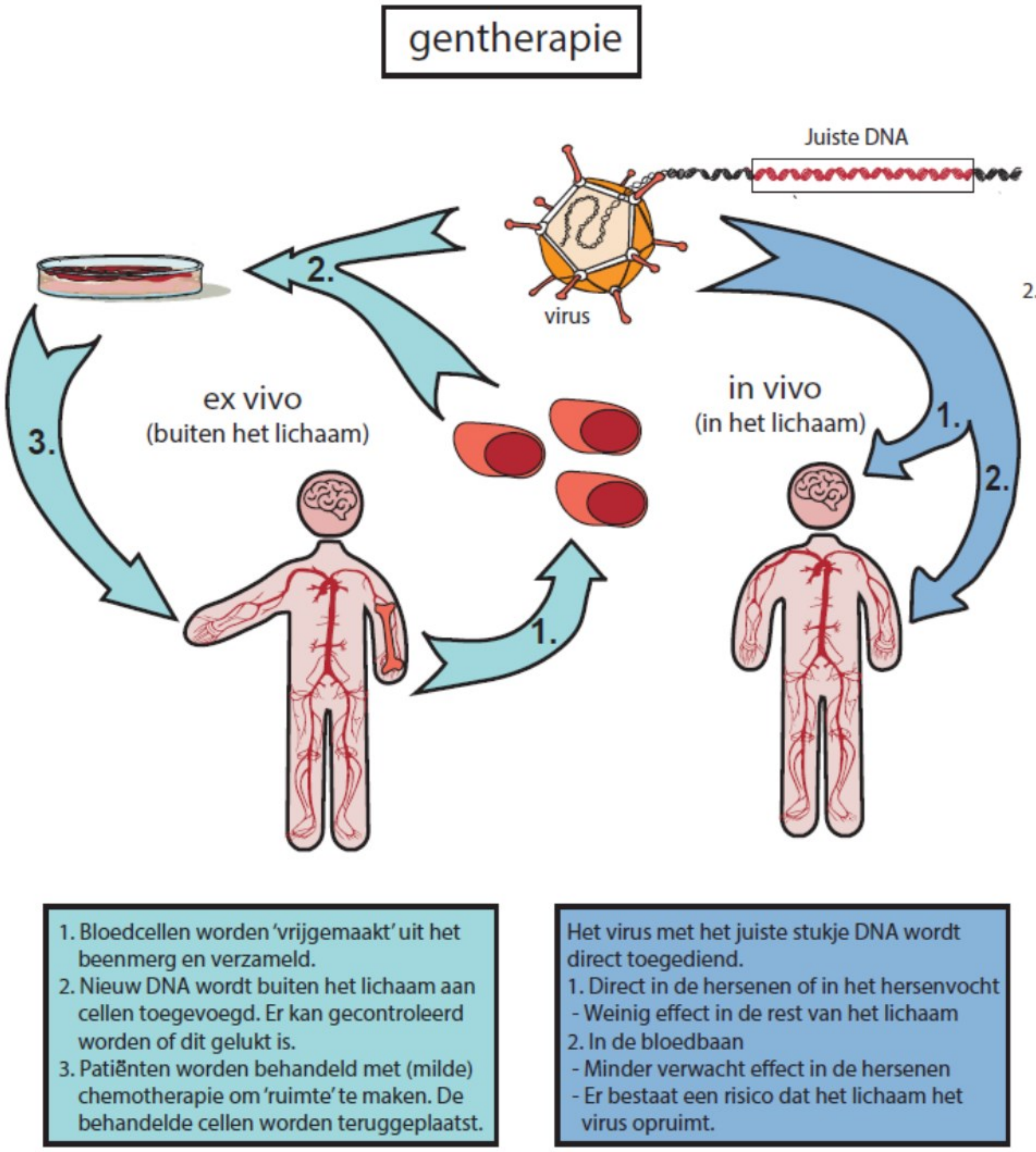

Volgende

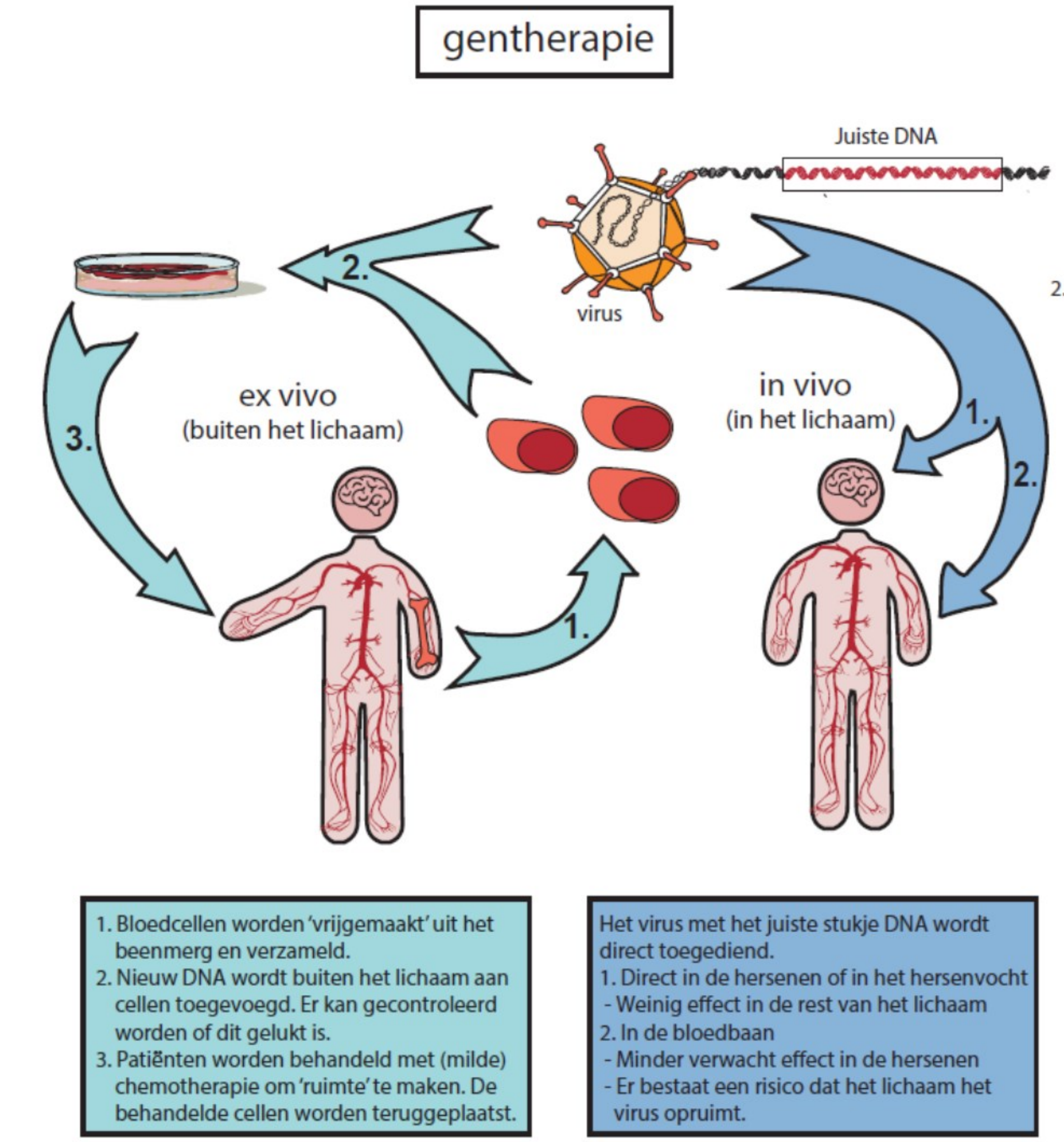

Bij de ene soort ("ex vivo") vindt in meerdere stappen plaats:
1. Er worden beenmergcellen uit het lichaam van patiënten gehaald via een bloedafname.
2. Buiten het lichaam worden de cellen behandeld met gentherapie.
3. Daarna worden de behandelde cellen teruggeplaatst in het lichaam.

- Voordelen van deze vorm van gentherapie: er is controle over de hoeveelheid gentherapie waar de specifieke cellen aan worden blootgesteld. Daarnaast kan het lichaam het DNA mogelijk minder makkelijk 'opruimen' omdat het al in de cellen zit.
- Nadelen van deze vorm van gentherapie: er moet eerst ruimte in het beenmerg komen om de 'nieuwe' cellen te laten uitgroeien voordat de cellen teruggeplaatst kunnen worden. Daarom moet er een milde vorm van chemotherapie worden gegeven. Deze behandeling is kortdurend.

Bij de andere soort ("in vivo") wordt een virusonderdeel waar je niet ziek van wordt in het lichaam gespoten (1.). Dit virusonderdeel heeft het 'nieuwe' DNA bij zich en bouwt dit in bepaalde lichaamscellen in.

- Voordeel van deze vorm van gentherapie: het is een behandeling die gericht in het brein gegeven wordt
- Nadeel van deze vorm van gentherapie: de therapie moet in de hersenen worden ingebracht, dit betekent dat een ingreep nodig is.

Op dit moment wordt er onderzoek gedaan naar beide soorten gentherapie voor MPS III. Bij de vragen die we u zullen stellen maken we geen onderscheid tussen de verschillende vormen.

Volgende

Achtergrondinformatie

In welke leeftijdcategorie valt uw kind?

- ◯ Jonger dan 18 jaar
- ◯ 18 jaar of ouder

Wat is het geslacht van uw kind?

- ◯ Man
- ◯ Vrouw
- ◯ Anders

Wie vult deze vragenlijst in namens uw kind

- ◯ Vader
- ◯ Moeder
- ◯ Vader en moeder
- ◯ Voogd
- ◯ Anders ______

Neemt uw kind op dit moment deel aan een studie waarbij therapie voor MPS III wordt onderzocht?

- ◯ Ja
- ◯ Nee

Volgende

Kunt u toelichten welke studie en/of therapie dit is?

Volgende

Keuzetaken

We vragen u straks steeds een keuze te maken uit twee behandelopties; geen therapie of gentherapie. De keuzes zullen erg op elkaar lijken, toch zijn er kleine verschillen. Het is belangrijk voor ons dat u de informatie op de volgende pagina's goed leest en alle keuzetaken invult, we zullen hier de verschillende kenmerken van de behandelingen toelichten.

We vragen bij de keuzetaken om uw persoonlijke mening, er zijn dus geen goede of foute antwoorden/keuzes.

Volgende

Uitleg over de werkzaamheid van de behandeling

Op dit moment is er geen therapie beschikbaar voor MPS III. De werkzaamheid van gentherapie is op dit moment nog onbekend en zal in studieverband onderzocht worden. We gaan er in deze vragenlijst van uit dat gentherapie ziekteverschijnselen vermindert en de ziekte vertraagt. Gentherapie is een eenmalige behandeling. Het ondergaan van gentherapie kan gepaard gaan met milde en/of ernstige bijwerkingen. Ook bestaat er een kans dat er extra medicatie gebruikt moet worden. Tot slot is er nog onzekerheid over of gentherapie goed blijft werken op de lange termijn. In de vragenlijst stellen we u vragen die steeds betrekking hebben op een van deze aspecten. We vragen u bij elke vraag een keuze te maken tussen gentherapie en geen therapie.

Let op: Indien uw kind op dit moment therapie in studieverband krijgt, vragen we u uit te gaan van de situatie van voordat uw kind aan deze studie meedeed.

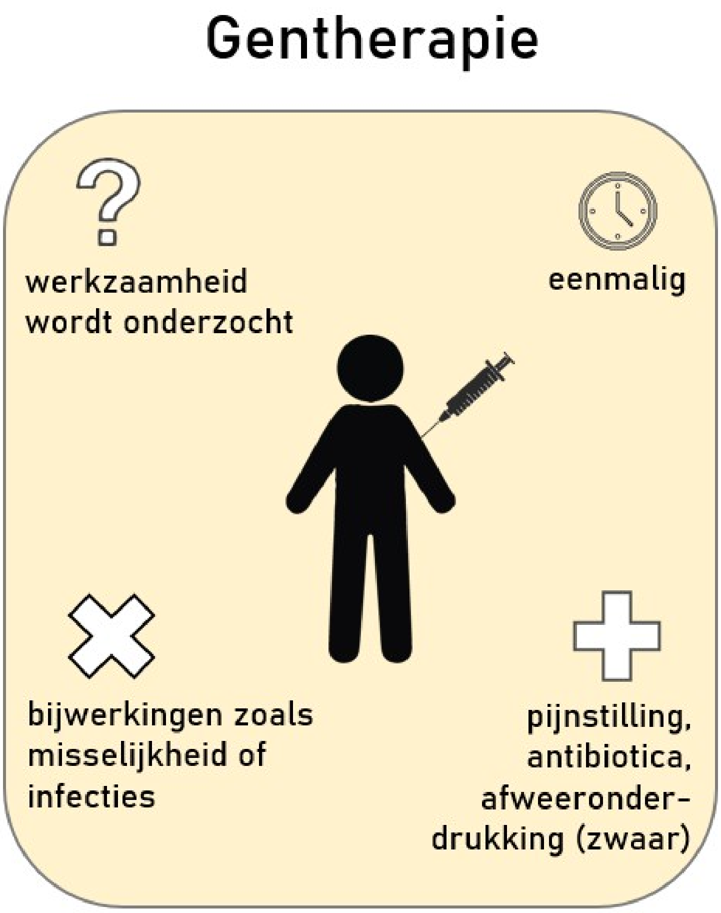

Volgende

Uitleg over mogelijke bijwerkingen van de behandelingen

Milde bijwerkingen: In de huidige situatie is geen therapie beschikbaar, daarom treden geen bijwerkingen op. Maar er is dus ook niets wat het verloop van de ziekte verandert. Bij het gebruik van gentherapie kunnen milde bijwerkingen optreden. Deze bijwerkingen duren kort, zijn zonder blijvende gevolgen en er is geen ziekenhuisopname nodig.

<u>Voor gentherapie</u>:
- het krijgen van een blauwe plek of bloeduitstorting
- tijdelijk algeheel niet lekker zijn (malaise)
- milde griepachtige klachten
- kortdurende misselijkheid of overgeven
- buikpijn of diarree
- ongevaarlijke infecties
- bloedarmoede
- pijnlijke ontstekingen van het mondslijmvlies
- koorts

Ernstige bijwerkingen: In de huidige situatie is geen therapie beschikbaar, daarom treden geen bijwerkingen op. Maar er is dus ook niets wat het verloop van de ziekte verandert. Bij het gebruik van gentherapie kunnen ernstige bijwerkingen optreden. Deze bijwerkingen hebben een ziekenhuisopname tot gevolg.

<u>Voor gentherapie</u>: ziekenhuisopnames vanwege bijvoorbeeld
- zeer hoge koorts
- ernstige infecties

Bij toediening van gentherapie in de hersenen of hersenvocht:
- overgeven
- bloeduitstortingen in het hoofd

Bij langdurige chronische ziektes zoals MPS III overlijden mensen over het algemeen niet aan de bijwerkingen van de gentherapiebehandeling. In de nog lopende gentherapie studies voor MPS III is één kind na behandeling overleden. Hierbij is niet duidelijk of dit te maken had met de behandeling of niet. De getallen in deze vragenlijst zijn grotendeels gebaseerd op studies met toediening van gentherapie via de bloedbaan, het zou kunnen dat de bijwerkingen vaker of minder vaak voorkomen bij toediening in de hersenen of hersenvocht maar dit is op dit moment niet bekend.

Gentherapie

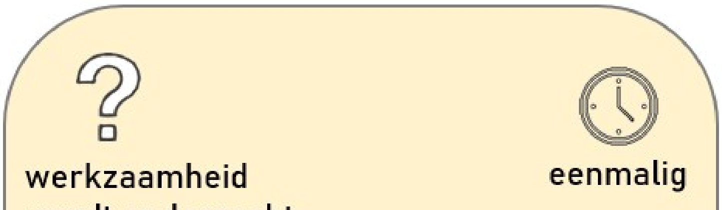

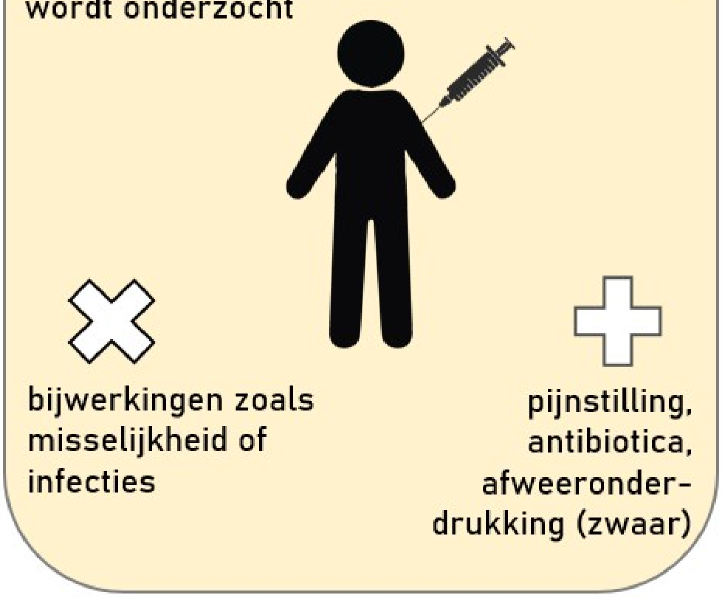
wordt onderzocht
bijwerkingen zoals misselijkheid of infecties
pijnstilling, antibiotica, afweeronder-drukking (zwaar)

Volgende

Uitleg over aanvullende medicatie en behandelfrequentie van de behandelingen.

Aanvullende medicatie: In de huidige situatie is geen therapie beschikbaar, daarom is er ook geen aanvullende medicatie nodig voor de bijwerkingen van een therapie. Het kan nodig zijn om de bijwerkingen van gentherapie te onderdrukken met medicatie. Dit kan zowel kortdurend als langdurig zijn. Met deze medicatie bedoelen we geen medicijnen die gebruikt worden om eventuele al bestaande problemen door de ziekte te behandelen, maar medicijnen die verschijnselen die de therapie veroorzaakt tegengaan (bijvoorbeeld misselijkheid door de gentherapie).

Voor gentherapie: Dit kan gaan om een immuunsysteem onderdrukkend middel, zoals prednison. Dat wordt kortdurend (weken tot maanden) gegeven om een afweerreactie van het lichaam tegen het (ongevaarlijke) virusonderdeel tegen te gaan. Daarnaast kan medicatie nodig zijn om bijwerkingen te verhelpen, zoals een antibioticakuur, pijnstilling, of middelen tegen misselijkheid.

Behandelfrequentie: In de huidige situatie is geen therapie beschikbaar. Voor gentherapie geldt dat de behandeling eenmalig is.

Gentherapie

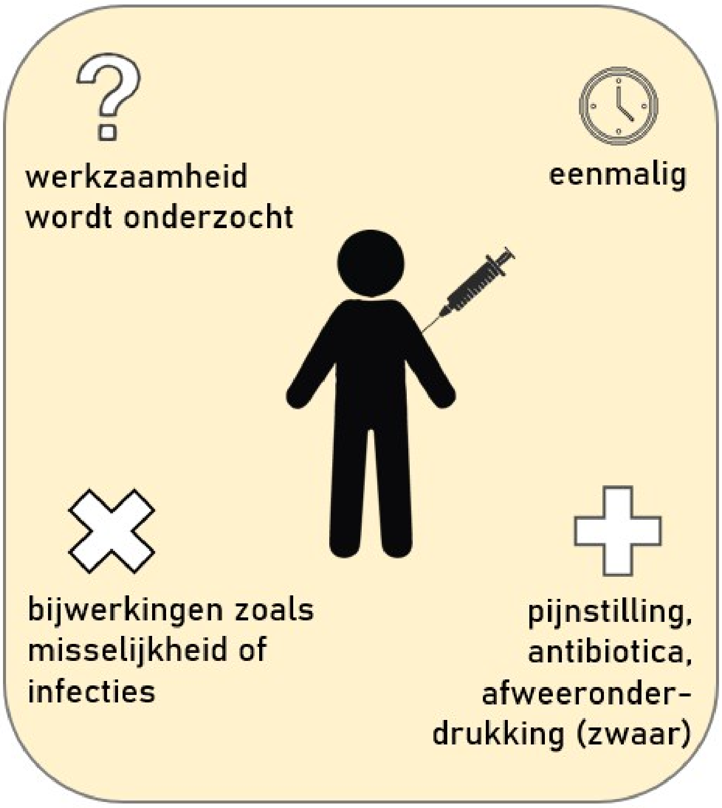

Volgende

Uitleg over waarschijnlijkheid en kansen

Voor verschillende kenmerken van behandelingen wordt straks geproken over 'waarschijnlijkheid'. Hiermee bedoelen we de kans dat de behandeling milde of ernstige bijwerkingen tot gevolg heeft of de kans dat uw kind aanvullende medicatie zou moeten nemen. Er worden verschillende kansen aan u gepresenteerd. Wanneer de waarschijnlijkheid (oftewel kans) op een bijwerking 40% is dan zullen 40 van elke 100 mensen die het medicijn nemen bijwerkingen krijgen terwijl 60 van de 100 mensen die het medicijn nemen geen bijwerkingen zullen ervaren. Dit ziet er als volgt uit:

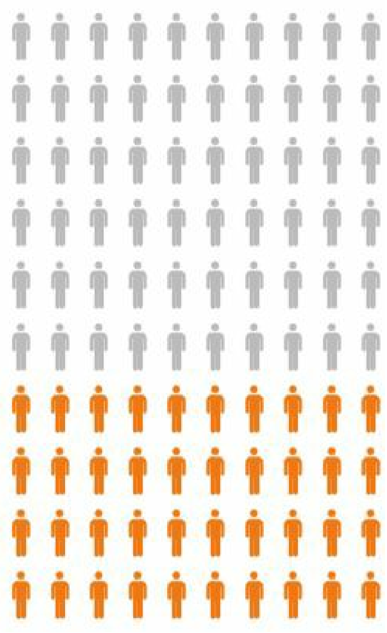

Volgende

Het invullen van de keuzetaken

We vragen u zo meerdere keren een keuze te maken uit twee behandelopties; geen behandeling en dus ook geen effect op het beloop van de ziekte zoals u die kent of gentherapie.

De keuzes zullen erg op elkaar lijken, toch zijn er kleine verschillen.

De keuzes zullen eruit zien zoals in het plaatje hieronder. Aan de linkerkant ziet u de eigenschappen van de behandelopties staan. Wanneer u straks de keuzetaken gaat invullen kunt u hier met uw muis op gaan staan, om de uitleg die hierbij hoort nogmaals lezen.

Bekijk de informatie in onderstaande tabel.

In de huidige situatie (of de situatie voordat uw kind therapie in studieverband ontving) is geen therapie beschikbaar, daarom treden ernstige bijwerkingen op bij 0% (0 van de 100 mensen). Stel dat bij gentherapie bij 20% (20 van de 100) van de mensen ernstige bijwerkingen optreden.

Let op: u kunt met uw muis over de kenmerken aan de linkerkant van de tabel gaan om de uitleg hiervan opnieuw te lezen.

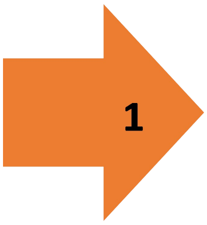

	Geen behandeling	Gentherapie
Werkzaamheid	-	Vermindering van ziekteverschijnselen en vertraging van ziekte
Kans op milde bijwerkingen	0% (0 van elke 100)	60% (60 van elke 100)
Kans op ernstige bijwerkingen	0% (0 van elke 100)	20% (20 van elke 100)
Kans dat aanvullende medicatie nodig is vanwege de therapie	0% (0 van elke 100)	15% (15 van elke 100)
Behandelfrequentie	-	Eenmalig

Op basis van de informatie in bovenstaande tabel, welke behandeling heeft dan uw voorkeur?

- ◯ Geen behandeling
- ◯ Gentherapie

Volgende

In het midden van de taak ziet u de behandelopties.

Beide behandelopties hebben bepaalde eigenschappen.

In het onderstaande voorbeeld heeft bij de gentherapie meer kans op ernstige bijwerkingen, maar gentherapie is een eenmalige behandeling die ziekteverschijnselen verminderd terwijl u bij geen behandeling geen effect zult zien.

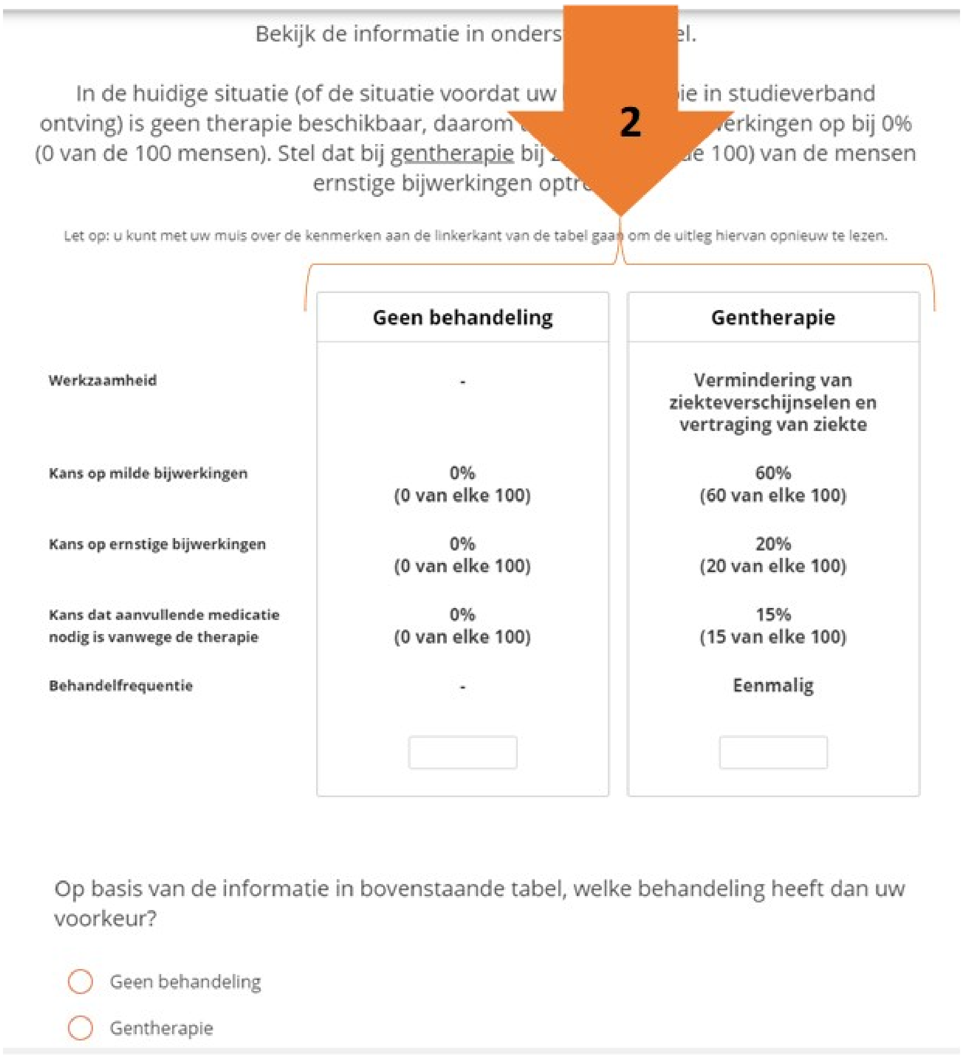

Bekijk de informatie in onders[illegible]el.

In de huidige situatie (of de situatie voordat uw [illegible]ie in studieverband ontving) is geen therapie beschikbaar, daarom [illegible]erkingen op bij 0% (0 van de 100 mensen). Stel dat bij gentherapie bij [illegible]e 100) van de mensen ernstige bijwerkingen optr[illegible]

Let op: u kunt met uw muis over de kenmerken aan de linkerkant van de tabel gaan om de uitleg hiervan opnieuw te lezen.

	Geen behandeling	Gentherapie
Werkzaamheid	-	Vermindering van ziekteverschijnselen en vertraging van ziekte
Kans op milde bijwerkingen	0% (0 van elke 100)	60% (60 van elke 100)
Kans op ernstige bijwerkingen	0% (0 van elke 100)	20% (20 van elke 100)
Kans dat aanvullende medicatie nodig is vanwege de therapie	0% (0 van elke 100)	15% (15 van elke 100)
Behandelfrequentie	-	Eenmalig

Op basis van de informatie in bovenstaande tabel, welke behandeling heeft dan uw voorkeur?

- Geen behandeling
- Gentherapie

Volgende

Vervolgens is het aan u om een afweging te maken tussen deze behandelopties en hun eigenschappen en een keuze te maken.

U kunt uw keuze bevestigen door:
1. In de tabel op de behandelopties van uw voorkeur te klikken
2. In de vraag onder de tabel dezelfde behandeloptie aan te klikken

Het is belangrijk dat u uw keuze in de tabel nogmaals bevestigt in de vraag onder elke tabel zoals in het voorbeeld hieronder waar de patient voor gentherapie heeft gekozen.

Bekijk de informatie in onderstaande tabel.

In de huidige situatie (of de situatie voordat uw kind therapie in studieverband ontving) is geen therapie beschikbaar, daarom treden ernstige bijwerkingen op bij 0% (0 van de 100 mensen). Stel dat bij gentherapie bij 20% (20 van de 100) van de mensen ernstige bijwerkingen optreden.

Let op: u kunt met uw muis over de kenmerken aan de linkerkant van de tabel gaan om de uitleg hiervan opnieuw te lezen.

	Geen behandeling	Gentherapie
Werkzaamheid	-	Vermindering van ziekteverschijnselen en vertraging van ziekte
Kans op milde bijwerkingen	0% (0 van elke 100)	60% (60 van elke 100)
Kans op ernstige bijwerkingen	0% (0 van elke 100)	20% (20 van elke 100)
Kans dat aanvullende medicatie nodig is vanwege de therapie	0% (0 van elke 100)	15% (15 van elke 100)
Behandelfrequentie	-	Eenmalig

3

Op basis van de informatie in bovenstaande tabel, welke behande[illegible]an uw voorkeur?

3

- ○ Geen behandeling
- ● Gentherapie

Volgende

Deel 1: Milde bijwerkingen

Bij dit onderdeel van de vragenlijst gaat het om milde bijwerkingen die kunnen optreden bij het gebruik van gentherapie. Deze bijwerkingen duren kort, zijn zonder blijvende gevolgen en er is geen ziekenhuisopname nodig.

Volgende

Bekijk de informatie in onderstaande tabel.

In de huidige situatie (of de situatie voordat uw kind therapie in studieverband ontving) is geen therapie beschikbaar, daarom treden milde bijwerkingen op bij 0% (0 van de 100 mensen). Stel dat bij gentherapie bij 60% (60 van de 100) van de mensen milde bijwerkingen optreden.

Let op: u kunt met uw muis over de kenmerken aan de linkerkant van de tabel gaan om de uitleg hiervan opnieuw te lezen.

	**Geen therapie**	**Gentherapie**
**Werkzaamheid**	-	Vermindering van ziekteverschijnselen en vertraging van ziekte
**Kans op milde bijwerkingen**	0%<br>(0 van elke 100)	60%<br>(60 van elke 100)
**Kans op ernstige bijwerkingen**	0%<br>(0 van elke 100)	50%<br>(50 van elke 100)
**Kans dat aanvullende medicatie nodig is vanwege de therapie**	0%<br>(0 van elke 100)	15%<br>(15 van elke 100)
**Behandelfrequentie**	-	Eenmalig

Op basis van de informatie in bovenstaande tabel, welke behandeling heeft dan uw voorkeur?

- ◯ Geen behandeling
- ◯ Gentherapie

Volgende

Bekijk de informatie in onderstaande tabel.

In de huidige situatie (of de situatie voordat uw kind therapie in studieverband ontving) is geen therapie beschikbaar, daarom treden milde bijwerkingen op bij 0% (0 van de 100 mensen). Stel dat bij gentherapie bij 40% (40 van de 100) van de mensen milde bijwerkingen optreden.

Let op: u kunt met uw muis over de kenmerken aan de linkerkant van de tabel gaan om de uitleg hiervan opnieuw te lezen.

	Geen behandeling	Gentherapie
**Werkzaamheid**	-	Vermindering van ziekteverschijnselen en vertraging van ziekte
**Kans op milde bijwerkingen**	0% (0 van elke 100)	40% (40 van elke 100)
**Kans op ernstige bijwerkingen**	0% (0 van elke 100)	50% (50 van elke 100)
**Kans dat aanvullende medicatie nodig is vanwege de therapie**	0% (0 van elke 100)	15% (15 van elke 100)
**Behandelfrequentie**	-	Eenmalig

Op basis van de informatie in bovenstaande tabel, welke behandeling heeft dan uw voorkeur?

- ◯ Geen behandeling
- ◯ Gentherapie

Volgende

Bekijk de informatie in onderstaande tabel.

In de huidige situatie (of de situatie voordat uw kind therapie in studieverband ontving) is geen therapie beschikbaar, daarom treden milde bijwerkingen op bij 0% (0 van de 100 mensen). Stel dat bij gentherapie bij 50% (50 van de 100) van de mensen milde bijwerkingen optreden.

Let op: u kunt met uw muis over de kenmerken aan de linkerkant van de tabel gaan om de uitleg hiervan opnieuw te lezen.

	**Geen behandeling**	**Gentherapie**
**Werkzaamheid**	-	**Vermindering van ziekteverschijnselen en vertraging van ziekte**
**Kans op milde bijwerkingen**	**0%<br>(0 van elke 100)**	**50%<br>(50 van elke 100)**
**Kans op ernstige bijwerkingen**	**0%<br>(0 van elke 100)**	**50%<br>(50 van elke 100)**
**Kans dat aanvullende medicatie nodig is vanwege de therapie**	**0%<br>(0 van elke 100)**	**15%<br>(15 van elke 100)**
**Behandelfrequentie**	-	**Eenmalig**

Op basis van de informatie in bovenstaande tabel, welke behandeling heeft dan uw voorkeur?

- ◯ Geen behandeling
- ◯ Gentherapie

Volgende

Bekijk de informatie in onderstaande tabel.

In de huidige situatie (of de situatie voordat uw kind therapie in studieverband ontving) is geen therapie beschikbaar, daarom treden milde bijwerkingen op bij 0% (0 van de 100 mensen). Stel dat bij gentherapie bij 80% (80 van de 100) van de mensen milde bijwerkingen optreden.

Let op: u kunt met uw muis over de kenmerken aan de linkerkant van de tabel gaan om de uitleg hiervan opnieuw te lezen.

	**Geen behandeling**	**Gentherapie**
**Werkzaamheid**	-	**Vermindering van ziekteverschijnselen en vertraging van ziekte**
**Kans op milde bijwerkingen**	**0%<br>(0 van elke 100)**	**80%<br>(80 van elke 100)**
**Kans op ernstige bijwerkingen**	**0%<br>(0 van elke 100)**	**50%<br>(50 van elke 100)**
**Kans dat aanvullende medicatie nodig is vanwege de therapie**	**0%<br>(0 van elke 100)**	**15%<br>(15 van elke 100)**
**Behandelfrequentie**	-	**Eenmalig**

Op basis van de informatie in bovenstaande tabel, welke behandeling heeft dan uw voorkeur?

- ◯ Geen behandeling
- ◯ Gentherapie

Volgende

Bekijk de informatie in onderstaande tabel.

In de huidige situatie (of de situatie voordat uw kind therapie in studieverband ontving) is geen therapie beschikbaar, daarom treden milde bijwerkingen op bij 0% (0 van de 100 mensen). Stel dat bij gentherapie bij 70% (70 van de 100) van de mensen milde bijwerkingen optreden.

Let op: u kunt met uw muis over de kenmerken aan de linkerkant van de tabel gaan om de uitleg hiervan opnieuw te lezen.

	Geen behandeling	Gentherapie
**Werkzaamheid**	-	**Vermindering van ziekteverschijnselen en vertraging van ziekte**
**Kans op milde bijwerkingen**	**0% (0 van elke 100)**	**70% (70 van elke 100)**
**Kans op ernstige bijwerkingen**	**0% (0 van elke 100)**	**50% (50 van elke 100)**
**Kans dat aanvullende medicatie nodig is vanwege de therapie**	**0% (0 van elke 100)**	**15% (15 van elke 100)**
**Behandelfrequentie**	-	**Eenmalig**

Op basis van de informatie in bovenstaande tabel, welke behandeling heeft dan uw voorkeur?

- ◯ Geen behandeling
- ◯ Gentherapie

Volgende

U heeft net aangegeven dat u geen behandeling zou kiezen als de kans op milde bijwerkingen bij gentherapie 40% is.

Wat is de hoogste kans (in %) op milde bijwerkingen waarbij u toch gentherapie zou kiezen? (let op: dit is dus altijd lager dan 40, maar kan ook 0 zijn indien u bijvoorbeeld helemaal geen gentherapie wil)

Volgende

U heeft net aangegeven dat u voor gentherapie zou kiezen als de kans op milde bijwerkingen 80% is.

Wat is de hoogste kans (in %) op milde bijwerkingen waarbij u nog gentherapie zou kiezen? (let op: dit is dus altijd hoger dan 80, maar kan ook 100 zijn indien u altijd voor gentherapie zou kiezen)

Volgende

Deel 2: Ernstige bijwerkingen

Bij dit onderdeel van de vragenlijst gaat het om ernstige bijwerkingen die kunnen optreden bij het gebruik van gentherapie. Deze bijwerkingen hebben een ziekenhuisopname, of in het meest ernstige geval overlijden tot gevolg.

Volgende

Bekijk de informatie in onderstaande tabel.

In de huidige situatie (of de situatie voordat uw kind therapie in studieverband ontving) is geen therapie beschikbaar, daarom treden ernstige bijwerkingen op bij 0% (0 van de 100 mensen). Stel dat bij gentherapie bij 50% (50 van de 100) van de mensen ernstige bijwerkingen optreden.

Let op: u kunt met uw muis over de kenmerken aan de linkerkant van de tabel gaan om de uitleg hiervan opnieuw te lezen.

	**Geen behandeling**	**Gentherapie**
**Werkzaamheid**	-	Vermindering van ziekteverschijnselen en vertraging van ziekte
**Kans op milde bijwerkingen**	0% (0 van elke 100)	60% (60 van elke 100)
**Kans op ernstige bijwerkingen**	0% (0 van elke 100)	50% (50 van elke 100)
**Kans dat aanvullende medicatie nodig is vanwege de therapie**	0% (0 van elke 100)	15% (15 van elke 100)
**Behandelfrequentie**	-	Eenmalig

Op basis van de informatie in bovenstaande tabel, welke behandeling heeft dan uw voorkeur?

- ◯ Geen behandeling
- ◯ Gentherapie

Volgende

Bekijk de informatie in onderstaande tabel.

In de huidige situatie (of de situatie voordat uw kind therapie in studieverband ontving) is geen therapie beschikbaar, daarom treden ernstige bijwerkingen op bij 0% (0 van de 100 mensen). Stel dat bij gentherapie bij 20% (20 van de 100) van de mensen ernstige bijwerkingen optreden.

Let op: u kunt met uw muis over de kenmerken aan de linkerkant van de tabel gaan om de uitleg hiervan opnieuw te lezen.

	**Geen behandeling**	**Gentherapie**
**Werkzaamheid**	**-**	**Vermindering van ziekteverschijnselen en vertraging van ziekte**
**Kans op milde bijwerkingen**	**0% (0 van elke 100)**	**60% (60 van elke 100)**
**Kans op ernstige bijwerkingen**	**0% (0 van elke 100)**	**20% (20 van elke 100)**
**Kans dat aanvullende medicatie nodig is vanwege de therapie**	**0% (0 van elke 100)**	**15% (15 van elke 100)**
**Behandelfrequentie**	**-**	**Eenmalig**

Op basis van de informatie in bovenstaande tabel, welke behandeling heeft dan uw voorkeur?

- ◯ Geen behandeling
- ◯ Gentherapie

Volgende

Bekijk de informatie in onderstaande tabel.

In de huidige situatie (of de situatie voordat uw kind therapie in studieverband ontving) is geen therapie beschikbaar, daarom treden ernstige bijwerkingen op bij 0% (0 van de 100 mensen). Stel dat bij gentherapie bij 35% (35 van de 100) van de mensen ernstige bijwerkingen optreden.

Let op: u kunt met uw muis over de kenmerken aan de linkerkant van de tabel gaan om de uitleg hiervan opnieuw te lezen.

	**Geen behandeling**	**Gentherapie**
**Werkzaamheid**	-	Vermindering van ziekteverschijnselen en vertraging van ziekte
**Kans op milde bijwerkingen**	0% (0 van elke 100)	60% (60 van elke 100)
**Kans op ernstige bijwerkingen**	0% (0 van elke 100)	35% (35 van elke 100)
**Kans dat aanvullende medicatie nodig is vanwege de therapie**	0% (0 van elke 100)	15% (15 van elke 100)
**Behandelfrequentie**	-	Eenmalig

Op basis van de informatie in bovenstaande tabel, welke behandeling heeft dan uw voorkeur?

- Geen behandeling
- Gentherapie

Volgende

Bekijk de informatie in onderstaande tabel.

In de huidige situatie (of de situatie voordat uw kind therapie in studieverband ontving) is geen therapie beschikbaar, daarom treden ernstige bijwerkingen op bij 0% (0 van de 100 mensen). Stel dat bij gentherapie bij 80% (80 van de 100) van de mensen ernstige bijwerkingen optreden.

Let op: u kunt met uw muis over de kenmerken aan de linkerkant van de tabel gaan om de uitleg hiervan opnieuw te lezen.

	Geen behandeling	Gentherapie
**Werkzaamheid**	-	Vermindering van ziekteverschijnselen en vertraging van ziekte
**Kans op milde bijwerkingen**	0%<br>(0 van elke 100)	60%<br>(60 van elke 100)
**Kans op ernstige bijwerkingen**	0%<br>(0 van elke 100)	80%<br>(80 van elke 100)
**Kans dat aanvullende medicatie nodig is vanwege de therapie**	0%<br>(0 van elke 100)	15%<br>(15 van elke 100)
**Behandelfrequentie**	-	Eenmalig

Op basis van de informatie in bovenstaande tabel, welke behandeling heeft dan uw voorkeur?

- ◯ Geen behandeling
- ◯ Gentherapie

Volgende

Bekijk de informatie in onderstaande tabel.

In de huidige situatie (of de situatie voordat uw kind therapie in studieverband ontving) is geen therapie beschikbaar, daarom treden ernstige bijwerkingen op bij 0% (0 van de 100 mensen). Stel dat bij gentherapie bij 65% (65 van de 100) van de mensen ernstige bijwerkingen optreden.

Let op: u kunt met uw muis over de kenmerken aan de linkerkant van de tabel gaan om de uitleg hiervan opnieuw te lezen.

	**Geen behandeling**	**Gentherapie**
**Werkzaamheid**	-	Vermindering van ziekteverschijnselen en vertraging van ziekte
**Kans op milde bijwerkingen**	0% (0 van elke 100)	60% (60 van elke 100)
**Kans op ernstige bijwerkingen**	0% (0 van elke 100)	65% (65 van elke 100)
**Kans dat aanvullende medicatie nodig is vanwege de therapie**	0% (0 van elke 100)	15% (15 van elke 100)
**Behandelfrequentie**	-	Eenmalig

Op basis van de informatie in bovenstaande tabel, welke behandeling heeft dan uw voorkeur?

- ○ Geen behandeling
- ○ Gentherapie

Volgende

U heeft net aangegeven dat u geen behandeling zou kiezen als de kans op ernstige bijwerkingen bij gentherapie 20% is.

Wat is de hoogste kans (in %) op ernstige bijwerkingen waarbij u toch gentherapie zou kiezen? (let op: dit is dus altijd lager dan 20, maar kan ook 0 zijn indien u bijvoorbeeld helemaal geen gentherapie wil)

Volgende

U heeft net aangegeven dat u voor gentherapie zou kiezen als de kans op ernstige bijwerkingen 80% is.

Wat is de hoogste kans (in %) op ernstige bijwerkingen waarbij u nog gentherapie zou kiezen? (let op: dit is dus altijd hoger dan 80, maar kan ook 100 zijn indien u altijd voor gentherapie zou kiezen)

Volgende

Deel 3: Aanvullende medicatie

Bij dit onderdeel van de vragenlijst gaat het om het gebruik van aanvullende medicatie, wat nodig kan zijn om de bijwerkingen van gentherapie te onderdrukken. Dit kan zowel kortdurend als langdurig zijn.

Volgende

Bekijk de informatie in onderstaande tabel.

In de huidige situatie (of de situatie voordat uw kind therapie in studieverband ontving) is geen therapie beschikbaar en daarom is er bij 0% (0 van de 100) van de mensen aanvullende medicatie nodig. Stel dat bij gentherapie bij 15% (15 van de 100) van de mensen aanvullende medicatie nodig hebben.

Let op: u kunt met uw muis over de kenmerken aan de linkerkant van de tabel gaan om de uitleg hiervan opnieuw te lezen.

	**Geen behandeling**	**Gentherapie**
**Werkzaamheid**	**-**	**Vermindering van ziekteverschijnselen en vertraging van ziekte**
**Kans op milde bijwerkingen**	**0%<br>(0 van elke 100)**	**60%<br>(60 van elke 100)**
**Kans op ernstige bijwerkingen**	**0%<br>(0 van elke 100)**	**50%<br>(50 van elke 100)**
**Kans dat aanvullende medicatie nodig is vanwege de therapie**	**0%<br>(0 van elke 100)**	**15%<br>(15 van elke 100)**
**Behandelfrequentie**	**-**	**Eenmalig**

Op basis van de informatie in bovenstaande tabel, welke behandeling heeft dan uw voorkeur?

- ◯ Geen behandeling
- ◯ Gentherapie

Volgende

Bekijk de informatie in onderstaande tabel.

In de huidige situatie (of de situatie voordat uw kind therapie in studieverband ontving) is geen therapie beschikbaar en daarom is er bij 0% (0 van de 100)van de mensen aanvullende medicatie nodig. Stel dat bij gentherapie bij 5% (5 van de 100) van de mensen aanvullende medicatie nodig hebben.

Let op: u kunt met uw muis over de kenmerken aan de linkerkant van de tabel gaan om de uitleg hiervan opnieuw te lezen.

	**Geen behandeling**	**Gentherapie**
**Werkzaamheid**	-	**Vermindering van ziekteverschijnselen en vertraging van ziekte**
**Kans op milde bijwerkingen**	**0%<br>(0 van elke 100)**	**60%<br>(60 van elke 100)**
**Kans op ernstige bijwerkingen**	**0%<br>(0 van elke 100)**	**50%<br>(50 van elke 100)**
**Kans dat aanvullende medicatie nodig is vanwege de therapie**	**0%<br>(0 van elke 100)**	**5%<br>(5 van elke 100)**
**Behandelfrequentie**	-	**Eenmalig**

Op basis van de informatie in bovenstaande tabel, welke behandeling heeft dan uw voorkeur?

- ○ Geen behandeling
- ○ Gentherapie

Volgende

Bekijk de informatie in onderstaande tabel.

In de huidige situatie (of de situatie voordat uw kind therapie in studieverband ontving) is geen therapie beschikbaar en daarom is er bij 0% (0 van de 100) van de mensen aanvullende medicatie nodig. Stel dat bij gentherapie bij 10% (10 van de 100) van de mensen aanvullende medicatie nodig hebben.

Let op: u kunt met uw muis over de kenmerken aan de linkerkant van de tabel gaan om de uitleg hiervan opnieuw te lezen.

	**Geen behandeling**	**Gentherapie**
**Werkzaamheid**	-	**Vermindering van ziekteverschijnselen en vertraging van ziekte**
**Kans op milde bijwerkingen**	**0%<br>(0 van elke 100)**	**60%<br>(60 van elke 100)**
**Kans op ernstige bijwerkingen**	**0%<br>(0 van elke 100)**	**50%<br>(50 van elke 100)**
**Kans dat aanvullende medicatie nodig is vanwege de therapie**	**0%<br>(0 van elke 100)**	**10%<br>(10 van elke 100)**
**Behandelfrequentie**	-	**Eenmalig**

Op basis van de informatie in bovenstaande tabel, welke behandeling heeft dan uw voorkeur?

- ◯ Geen behandeling
- ◯ Gentherapie

Volgende

Bekijk de informatie in onderstaande tabel.

In de huidige situatie (of de situatie voordat uw kind therapie in studieverband ontving) is geen therapie beschikbaar en daarom is er bij 0% (0 van de 100) van de mensen aanvullende medicatie nodig. Stel dat bij gentherapie bij 50% (50 van de 100) van de mensen aanvullende medicatie nodig hebben.

Let op: u kunt met uw muis over de kenmerken aan de linkerkant van de tabel gaan om de uitleg hiervan opnieuw te lezen.

	**Geen behandeling**	**Gentherapie**
**Werkzaamheid**	**-**	**Vermindering van ziekteverschijnselen en vertraging van ziekte**
**Kans op milde bijwerkingen**	**0%<br>(0 van elke 100)**	**60%<br>(60 van elke 100)**
**Kans op ernstige bijwerkingen**	**0%<br>(0 van elke 100)**	**50%<br>(50 van elke 100)**
**Kans dat aanvullende medicatie nodig is vanwege de therapie**	**0%<br>(0 van elke 100)**	**50%<br>(50 van elke 100)**
**Behandelfrequentie**	**-**	**Eenmalig**

Op basis van de informatie in bovenstaande tabel, welke behandeling heeft dan uw voorkeur?

- ◯ Geen behandeling
- ◯ Gentherapie

Volgende

Bekijk de informatie in onderstaande tabel.

In de huidige situatie (of de situatie voordat uw kind therapie in studieverband ontving) is geen therapie beschikbaar en daarom is er bij 0% (0 van de 100) van de mensen aanvullende medicatie nodig. Stel dat bij gentherapie bij 30% (30 van de 100) van de mensen aanvullende medicatie nodig hebben.

Let op: u kunt met uw muis over de kenmerken aan de linkerkant van de tabel gaan om de uitleg hiervan opnieuw te lezen.

	**Geen behandeling**	**Gentherapie**
**Werkzaamheid**	-	**Vermindering van ziekteverschijnselen en vertraging van ziekte**
**Kans op milde bijwerkingen**	**0% (0 van elke 100)**	**60% (60 van elke 100)**
**Kans op ernstige bijwerkingen**	**0% (0 van elke 100)**	**50% (50 van elke 100)**
**Kans dat aanvullende medicatie nodig is vanwege de therapie**	**0% (0 van elke 100)**	**30% (30 van elke 100)**
**Behandelfrequentie**	-	**Eenmalig**

Op basis van de informatie in bovenstaande tabel, welke behandeling heeft dan uw voorkeur?

- ◯ Geen behandeling
- ◯ Gentherapie

Volgende

U heeft net aangegeven dat u geen behandeling zou kiezen als de kans op aanvullende medicatie bij gentherapie 5% is.

Wat is de hoogste kans (in %) op aanvullende medicatie waarbij u toch gentherapie zou kiezen? (let op: dit is dus altijd lager dan 5, maar kan ook 0 zijn indien u bijvoorbeeld helemaal geen gentherapie wil)

Volgende

U heeft net aangegeven dat u voor gentherapie zou kiezen als de kans op aanvullende medicatie 50% is.

Wat is de hoogste kans (in %) op aanvullende medicatie waarbij u nog gentherapie zou kiezen? (let op: dit is dus altijd hoger dan 50, maar kan ook 100 zijn indien u altijd voor gentherapie zou kiezen)

Volgende

Deel 4: onzekerheidsvraag

Bij de keuze om wel of niet mee te doen aan een studie naar een nieuw geneesmiddel kan ook de onzekerheid over werkzaamheid en bijwerkingen meespelen. Deze vraag gaat over die onzekerheid.

Volgende

Bekijk de informatie in onderstaande tabel.

We gaan er in dit scenario van uit dat gentherapie een beter effect heeft op de situatie van uw kind dan eerder in deze vragenlijst. Of gentherapie daadwerkelijk veilig en werkzaam is, moet nog uitgezocht worden in studieverband. Stel dat de kans 25% is dat gentherapie veilig en werkzaam blijkt.

Let op: u kunt met uw muis over de kenmerken aan de linkerkant van de tabel gaan om de uitleg hiervan opnieuw te lezen.

	Geen behandeling	Gentherapie
**Werkzaamheid**	-	**Vermindering van ziekteverschijnselen en vertraging van ziekte**
**Kans dat het middel op langere termijn als veilig en effectief wordt beschouwd**	-	**25%**
**Kans op milde bijwerkingen**	**0% (0 van elke 100)**	**60% (60 van elke 100)**
**Kans op ernstige bijwerkingen**	**0% (0 van elke 100)**	**50% (50 van elke 100)**
**Kans dat aanvullende medicatie nodig is vanwege de therapie**	**0% (0 van elke 100)**	**15% (15 van elke 100)**
**Behandelfrequentie**	-	**Eenmalig**

Op basis van de informatie in bovenstaande tabel, welke behandeling heeft dan uw voorkeur?

- ◯ Geen behandeling
- ◯ Gentherapie

Volgende

Bekijk de informatie in onderstaande tabel.

We gaan er in dit scenario van uit dat gentherapie een beter effect heeft op de situatie van uw kind dan eerder in deze vragenlijst. Of gentherapie daadwerkelijk veilig en werkzaam is, moet nog uitgezocht worden in studieverband. Stel dat de kans 5% is dat gentherapie veilig en werkzaam blijkt.

Let op: u kunt met uw muis over de kenmerken aan de linkerkant van de tabel gaan om de uitleg hiervan opnieuw te lezen.

	**Uw huidige therapie**	**Gentherapie**
**Werkzaamheid**	-	Vermindering van ziekteverschijnselen en vertraging van ziekte
**Kans dat het middel op langere termijn als veilig en effectief wordt beschouwd**	-	5%
**Kans op milde bijwerkingen**	0%<br>(0 van elke 100)	60%<br>(60 van elke 100)
**Kans op ernstige bijwerkingen**	0%<br>(0 van elke 100)	50%<br>(50 van elke 100)
**Kans dat aanvullende medicatie nodig is vanwege de therapie**	0%<br>(0 van elke 100)	15%<br>(15 van elke 100)
**Behandelfrequentie**	-	Eenmalig

Op basis van de informatie in bovenstaande tabel, welke behandeling heeft dan uw voorkeur?

- ◯ Geen behandeling
- ◯ Gentherapie

Volgende

Bekijk de informatie in onderstaande tabel.

We gaan er in dit scenario van uit dat gentherapie een beter effect heeft op de situatie van uw kind dan eerder in deze vragenlijst. Of gentherapie daadwerkelijk veilig en werkzaam is, moet nog uitgezocht worden in studieverband. Stel dat de kans 10% is dat gentherapie veilig en werkzaam blijkt.

Let op: u kunt met uw muis over de kenmerken aan de linkerkant van de tabel gaan om de uitleg hiervan opnieuw te lezen.

	**Geen behandeling**	**Gentherapie**
**Werkzaamheid**	**-**	**Vermindering van ziekteverschijnselen en vertraging van ziekte**
**Kans dat het middel op langere termijn als veilig en effectief wordt beschouwd**	**-**	**10%**
**Kans op milde bijwerkingen**	**0%<br>(0 van elke 100)**	**60%<br>(60 van elke 100)**
**Kans op ernstige bijwerkingen**	**0%<br>(0 van elke 100)**	**50%<br>(50 van elke 100)**
**Kans dat aanvullende medicatie nodig is vanwege de therapie**	**0%<br>(0 van elke 100)**	**15%<br>(15 van elke 100)**
**Behandelfrequentie**	**-**	**Eenmalig**

Op basis van de informatie in bovenstaande tabel, welke behandeling heeft dan uw voorkeur?

- ◯ Geen behandeling
- ◯ Gentherapie

Volgende

Bekijk de informatie in onderstaande tabel.

We gaan er in dit scenario van uit dat gentherapie een beter effect heeft op de situatie van uw kind dan eerder in deze vragenlijst. Of gentherapie daadwerkelijk veilig en werkzaam is, moet nog uitgezocht worden in studieverband. Stel dat de kans 50% is dat gentherapie veilig en werkzaam blijkt.

Let op: u kunt met uw muis over de kenmerken aan de linkerkant van de tabel gaan om de uitleg hiervan opnieuw te lezen.

	**Geen behandeling**	**Gentherapie**
**Werkzaamheid**	-	**Vermindering van ziekteverschijnselen en vertraging van ziekte**
**Kans dat het middel op langere termijn als veilig en effectief wordt beschouwd**	-	**50%**
**Kans op milde bijwerkingen**	**0%<br>(0 van elke 100)**	**60%<br>(60 van elke 100)**
**Kans op ernstige bijwerkingen**	**0%<br>(0 van elke 100)**	**50%<br>(50 van elke 100)**
**Kans dat aanvullende medicatie nodig is vanwege de therapie**	**0%<br>(0 van elke 100)**	**15%<br>(15 van elke 100)**
**Behandelfrequentie**	-	**Eenmalig**

Op basis van de informatie in bovenstaande tabel, welke behandeling heeft dan uw voorkeur?

- ◯ Geen behandeling
- ◯ Gentherapie

Volgende

Bekijk de informatie in onderstaande tabel.

We gaan er in dit scenario van uit dat gentherapie een beter effect heeft op de situatie van uw kind dan eerder in deze vragenlijst. Of gentherapie daadwerkelijk veilig en werkzaam is, moet nog uitgezocht worden in studieverband. Stel dat de kans 30% is dat gentherapie veilig en werkzaam blijkt.

Let op: u kunt met uw muis over de kenmerken aan de linkerkant van de tabel gaan om de uitleg hiervan opnieuw te lezen.

	**Geen behandeling**	**Gentherapie**
**Werkzaamheid**	-	Vermindering van ziekteverschijnselen en vertraging van ziekte
**Kans dat het middel op langere termijn als veilig en effectief wordt beschouwd**	-	30%
**Kans op milde bijwerkingen**	0%<br>(0 van elke 100)	60%<br>(60 van elke 100)
**Kans op ernstige bijwerkingen**	0%<br>(0 van elke 100)	50%<br>(50 van elke 100)
**Kans dat aanvullende medicatie nodig is vanwege de therapie**	0%<br>(0 van elke 100)	15%<br>(15 van elke 100)
**Behandelfrequentie**	-	Eenmalig

Op basis van de informatie in bovenstaande tabel, welke behandeling heeft dan uw voorkeur?

◯ Geen behandeling

◯ Gentherapie

Volgende

U heeft net aangegeven dat u voor gentherapie zou kiezen als de kans dat gentherapie in de komende jaren als veilig en effectief wordt beschouwd 5% is.

Wat is de minimale kans (in %) dat het middel in de komende jaren als veilig en effectief wordt beschouwd waarbij u nog voor gentherapie zou kiezen? (let op: dit is dus altijd lager dan 5, maar kan ook 0 zijn indien u altijd gentherapie zou kiezen)

Volgende

U heeft net aangegeven dat u geen behandeling zou kiezen als de kans dat gentherapie in de komende jaren als veilig en effectief wordt beschouwd 50% is.

Wat is de minimale kans (in %) dat het middel in de komende jaren als veilig en effectief wordt beschouwd waarbij u toch voor gentherapie zou kiezen? (let op: dit is dus altijd hoger dan 50, maar kan ook 100 zijn indien u nooit voor gentherapie zou kiezen)

Volgende

Deel 5: Stel gentherapie werkt beter

We gaan er in dit scenario van uit dat gentherapie een beter effect heeft dan eerder in deze vragenlijst.

Volgende

Bekijk de informatie in onderstaande tabel.

We gaan er in dit scenario van uit dat gentherapie een beter effect heeft op de situatie van uw kind dan eerder in deze vragenlijst. Of gentherapie daadwerkelijk veilig en werkzaam is, moet nog uitgezocht worden in studieverband. Stel dat de kans 25% is dat gentherapie veilig en werkzaam blijkt.

Let op: u kunt met uw muis over de kenmerken aan de rechterkant van de tabel gaan om de uitleg hiervan opnieuw te lezen.

	Geen behandeling	Gentherapie
**Werkzaamheid**	-	Beter effect dan eerder in de vragenlijst: er komen geen (nieuwe) ziekteverschijnselen meer bij, de ziekte stabiliseert.
**Kans dat het middel op langere termijn als veilig en effectief wordt beschouwd**	-	25%
**Kans op milde bijwerkingen**	0% (0 van elke 100)	60% (60 van elke 100)
**Kans op ernstige bijwerkingen**	0% (0 van elke 100)	50% (50 van elke 100)
**Kans dat aanvullende medicatie nodig is vanwege de therapie**	0% (0 van elke 100)	15% (15 van elke 100)
**Behandelfrequentie**	-	Eenmalig

Op basis van de informatie in bovenstaande tabel, welke behandeling heeft dan uw voorkeur?

- ◯ Geen behandeling
- ◯ Gentherapie

Volgende

Bekijk de informatie in onderstaande tabel.

We gaan er in dit scenario van uit dat gentherapie een beter effect heeft op de situatie van uw kind dan eerder in deze vragenlijst. Of gentherapie daadwerkelijk veilig en werkzaam is, moet nog uitgezocht worden in studieverband. Stel dat de kans 5% is dat gentherapie veilig en werkzaam blijkt.

Let op: u kunt met uw muis over de kenmerken aan de linkerkant van de tabel gaan om de uitleg hiervan opnieuw te lezen.

	**Geen behandeling**	**Gentherapie**
**Werkzaamheid**	-	**Beter effect dan eerder in de vragenlijst: er komen geen (nieuwe) ziekteverschijnselen meer bij, de ziekte stabiliseert.**
**Kans dat het middel op langere termijn als veilig en effectief wordt beschouwd**	-	**5%**
**Kans op milde bijwerkingen**	**0%<br>(0 van elke 100)**	**60%<br>(60 van elke 100)**
**Kans op ernstige bijwerkingen**	**0%<br>(0 van elke 100)**	**50%<br>(50 van elke 100)**
**Kans dat aanvullende medicatie nodig is vanwege de therapie**	**0%<br>(0 van elke 100)**	**15%<br>(15 van elke 100)**
**Behandelfrequentie**	-	**Eenmalig**

Op basis van de informatie in bovenstaande tabel, welke behandeling heeft dan uw voorkeur?

- Geen behandeling
- Gentherapie

Volgende

Bekijk de informatie in onderstaande tabel.

We gaan er in dit scenario van uit dat gentherapie een beter effect heeft op de situatie van uw kind dan eerder in deze vragenlijst. Of gentherapie daadwerkelijk veilig en werkzaam is, moet nog uitgezocht worden in studieverband. Stel dat de kans 10% is dat gentherapie veilig en werkzaam blijkt.

Let op: u kunt met uw muis over de kenmerken aan de linkerkant van de tabel gaan om de uitleg hiervan opnieuw te lezen.

	Geen behandeling	Gentherapie
**Werkzaamheid**	-	**Beter effect dan eerder in de vragenlijst: er komen geen (nieuwe) ziekteverschijnselen meer bij, de ziekte stabiliseert.**
**Kans dat het middel op langere termijn als veilig en effectief wordt beschouwd**	-	**10%**
**Kans op milde bijwerkingen**	**0%<br>(0 van elke 100)**	**60%<br>(60 van elke 100)**
**Kans op ernstige bijwerkingen**	**0%<br>(0 van elke 100)**	**50%<br>(50 van elke 100)**
**Kans dat aanvullende medicatie nodig is vanwege de therapie**	**0%<br>(0 van elke 100)**	**15%<br>(15 van elke 100)**
**Behandelfrequentie**	-	**Eenmalig**

Op basis van de informatie in bovenstaande tabel, welke behandeling heeft dan uw voorkeur?

- ○ Geen behandeling
- ○ Gentherapie

Volgende

Bekijk de informatie in onderstaande tabel.

We gaan er in dit scenario van uit dat gentherapie een beter effect heeft op de situatie van uw kind dan eerder in deze vragenlijst. Of gentherapie daadwerkelijk veilig en werkzaam is, moet nog uitgezocht worden in studieverband. Stel dat de kans 50% is dat gentherapie veilig en werkzaam blijkt.

Let op: u kunt met uw muis over de kenmerken aan de linkerkant van de tabel gaan om de uitleg hiervan opnieuw te lezen.

	**Geen behandeling**	**Gentherapie**
**Werkzaamheid**	-	**Beter effect dan eerder in de vragenlijst: er komen geen (nieuwe) ziekteverschijnselen meer bij, de ziekte stabiliseert.**
**Kans dat het middel op langere termijn als veilig en effectief wordt beschouwd**	-	**50%**
**Kans op milde bijwerkingen**	**0% (0 van elke 100)**	**60% (60 van elke 100)**
**Kans op ernstige bijwerkingen**	**0% (0 van elke 100)**	**50% (50 van elke 100)**
**Kans dat aanvullende medicatie nodig is vanwege de therapie**	**0% (0 van elke 100)**	**15% (15 van elke 100)**
**Behandelfrequentie**	-	**Eenmalig**

Op basis van de informatie in bovenstaande tabel, welke behandeling heeft dan uw voorkeur?

- ◯ Geen behandeling
- ◯ Gentherapie

Volgende

Bekijk de informatie in onderstaande tabel.

We gaan er in dit scenario van uit dat gentherapie een beter effect heeft op de situatie van uw kind dan eerder in deze vragenlijst. Of gentherapie daadwerkelijk veilig en werkzaam is, moet nog uitgezocht worden in studieverband. Stel dat de kans 30% is dat gentherapie veilig en werkzaam blijkt.

Let op: u kunt met uw muis over de kenmerken aan de linkerkant van de tabel gaan om de uitleg hiervan opnieuw te lezen.

	**Geen behandeling**	**Gentherapie**
**Werkzaamheid**	-	**Beter effect dan eerder in de vragenlijst: er komen geen (nieuwe) ziekteverschijnselen meer bij, de ziekte stabiliseert.**
**Kans dat het middel op langere termijn als veilig en effectief wordt beschouwd**	-	**30%**
**Kans op milde bijwerkingen**	**0%<br>(0 van elke 100)**	**60%<br>(60 van elke 100)**
**Kans op ernstige bijwerkingen**	**0%<br>(0 van elke 100)**	**50%<br>(50 van elke 100)**
**Kans dat aanvullende medicatie nodig is vanwege de therapie**	**0%<br>(0 van elke 100)**	**15%<br>(15 van elke 100)**
**Behandelfrequentie**	-	**Eenmalig**

Op basis van de informatie in bovenstaande tabel, welke behandeling heeft dan uw voorkeur?

- ◯ Geen behandeling
- ◯ Gentherapie

Volgende

U heeft net aangegeven dat u voor gentherapie zou kiezen als de kans dat gentherapie in de komende jaren als veilig en effectief wordt beschouwd 5% is.

Wat is de minimale kans (in %) dat het middel in de komende jaren als veilig en effectief wordt beschouwd waarbij u nog voor gentherapie zou kiezen? (let op: dit is dus altijd lager dan 5, maar kan ook 0 zijn indien u altijd gentherapie zou kiezen)

Volgende

U heeft net aangegeven dat u geen behandeling zou kiezen als de kans dat gentherapie in de komende jaren als veilig en effectief wordt beschouwd 50% is.

Wat is de minimale kans (in %) dat het middel in de komende jaren als veilig en effectief wordt beschouwd waarbij u toch voor gentherapie zou kiezen? (let op: dit is dus altijd hoger dan 50, maar kan ook 100 zijn indien u nooit voor gentherapie zou kiezen)

Volgende

Heeft uw kind in het verleden ooit aan een studie naar een nieuwe therapie voor MPS III meegedaan? Studies waar uw kind op dit moment nog aan meedoet tellen ook mee.

- ◯ Ja
- ◯ Nee

Volgende

Welke studie(s) was/waren dit?

Volgende

Gebruikt uw kind medicijnen voor klachten die komen door MPS III (zoals epilepsie of slaapproblemen)?

- ◯ Ja
- ◯ Nee

Volgende

Uw mening over de medicijnen die uw kind gebruikt

Als laatste onderdeel van deze vragenlijst willen we u vragen om per vraag het antwoord te kiezen dat het beste uw mening weergeeft over medicatie die uw kind voorgeschreven heeft gekregen of nog krijgt. Het gaat hierbij specifiek om medicatie die uw kind krijgt vanwege de ziekteverschijnselen van MPS III (zoals medicatie tegen epileptische aanvallen). We vragen u om medicijnen die uw kind eventueel in het kader van andere aandoeningen voorgeschreven krijgt hier niet mee te wegen in uw keuze.

U kunt per vraag een antwoord kiezen.

	Helemaal niet mee eens	Niet mee eens	Geen duidelijke mening	Mee eens	Helemaal mee eens
Op het moment hangt de gezondheid van mijn kind af van zijn/haar medicijnen	◯	◯	◯	◯	◯
Ik maak me zorgen over het feit dat mijn kind medicijnen moet nemen	◯	◯	◯	◯	◯
Het leven van mijn kind zou erg moeilijk zijn zonder medicijnen	◯	◯	◯	◯	◯
Soms maak ik me zorgen over de effecten die de medicijnen van mijn kind op de langere termijn kunnen hebben	◯	◯	◯	◯	◯
Zonder zijn/haar medicijnen zou mijn kind heel ziek zijn	◯	◯	◯	◯	◯
Ik ben onvoldoende op de hoogte van wat de medicijnen van mijn kind doen	◯	◯	◯	◯	◯
De toekomstige gezondheid van mijn kind hangt af van zijn/haar medicijnen	◯	◯	◯	◯	◯
De medicijnen van mijn kind ontwrichten zijn/haar leven	◯	◯	◯	◯	◯
Soms ben ik bang dat mijn kind te afhankelijk zal worden van zijn/haar medicijnen	◯	◯	◯	◯	◯
De medicijnen van mijn kind voorkomen dat hij/zij verder achteruitgaat	◯	◯	◯	◯	◯
Deze medicijnen hebben onplezierige bijwerkingen	◯	◯	◯	◯	◯

Volgende

Afronding vragenlijst & contact

U bent bij het einde van de vragenlijst aangekomen. We willen u van harte bedanken voor uw tijd en het beantwoorden van de vragen. Een samenvatting van onze bevindingen uit dit onderzoek zal na het verwerken van de resultaten met alle deelnemers van dit vragenlijstonderzoek via email worden gedeeld.

Heeft u nog vragen of opmerkingen over de vragenlijst, dit onderzoek of het onderwerp?

Volgende

Nogmaals hartelijk dank voor uw medewerking.

Als u aanvullende achtergrond informatie wenst over gentherapie voor stofwisselingsziekten willen we u wijzen op deze uitgebreide informatievideo van patientenvereniging VKS. Klik hier om deze video te bekijken.
