# Supplementary material for: Different diseases, different needs: Patient preferences for gene therapy in lysosomal storage disorders, a probabilistic threshold technique survey
Source: Orphanet J Rare Dis. 2024 Oct 3;19:367. doi: 10.1186/s13023-024-03371-y (PMC11451020; doi:10.1186/s13023-024-03371-y)
Supplement: Supplementary file 2 — Additional file 2. [file 13023_2024_3371_MOESM2_ESM.docx]

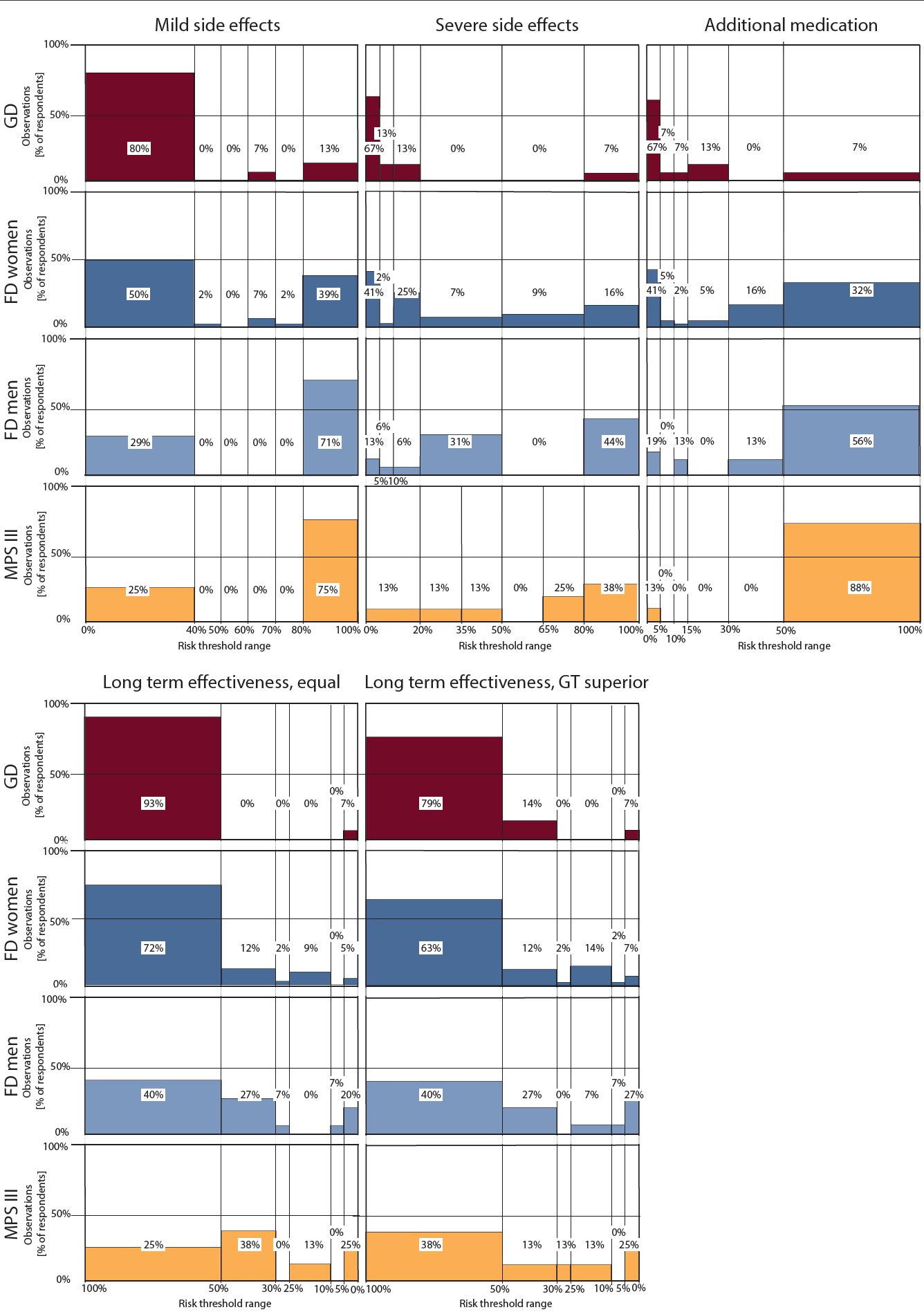


**Supplemental Fig 1 – Risk threshold ranges per task.** The risk threshold range of participants depicted per attribute and per disease group. On the x-axis the risk threshold ranges per attribute are depicted, on the y-axis the percentage of participants that ended within that specific range. The risk threshold ranges differ for MPS III in the severe side effects attribute, because intracerebral GT approaches were also considered in MPS III, which involve a higher risk of severe side effects. For the attributes long term effectiveness assuming equal or superior effectiveness of GT, the risk threshold ranges are depicted from high to low on the x-axis, since conversely to the other attributes a high percentage equals low risk tolerance.

Abbreviations: *FD* Fabry disease, *GD* Gaucher disease type 1, *GT* gene therapy, *MPS III* Mucopolysaccharidosis type III A/B
