# Supplementary material for: Different diseases, different needs: Patient preferences for gene therapy in lysosomal storage disorders, a probabilistic threshold technique survey
Source: Orphanet J Rare Dis. 2024 Oct 3;19:367. doi: 10.1186/s13023-024-03371-y (PMC11451020; doi:10.1186/s13023-024-03371-y)
Supplement: Supplementary file 3 — Additional file 3. [file 13023_2024_3371_MOESM3_ESM.docx]

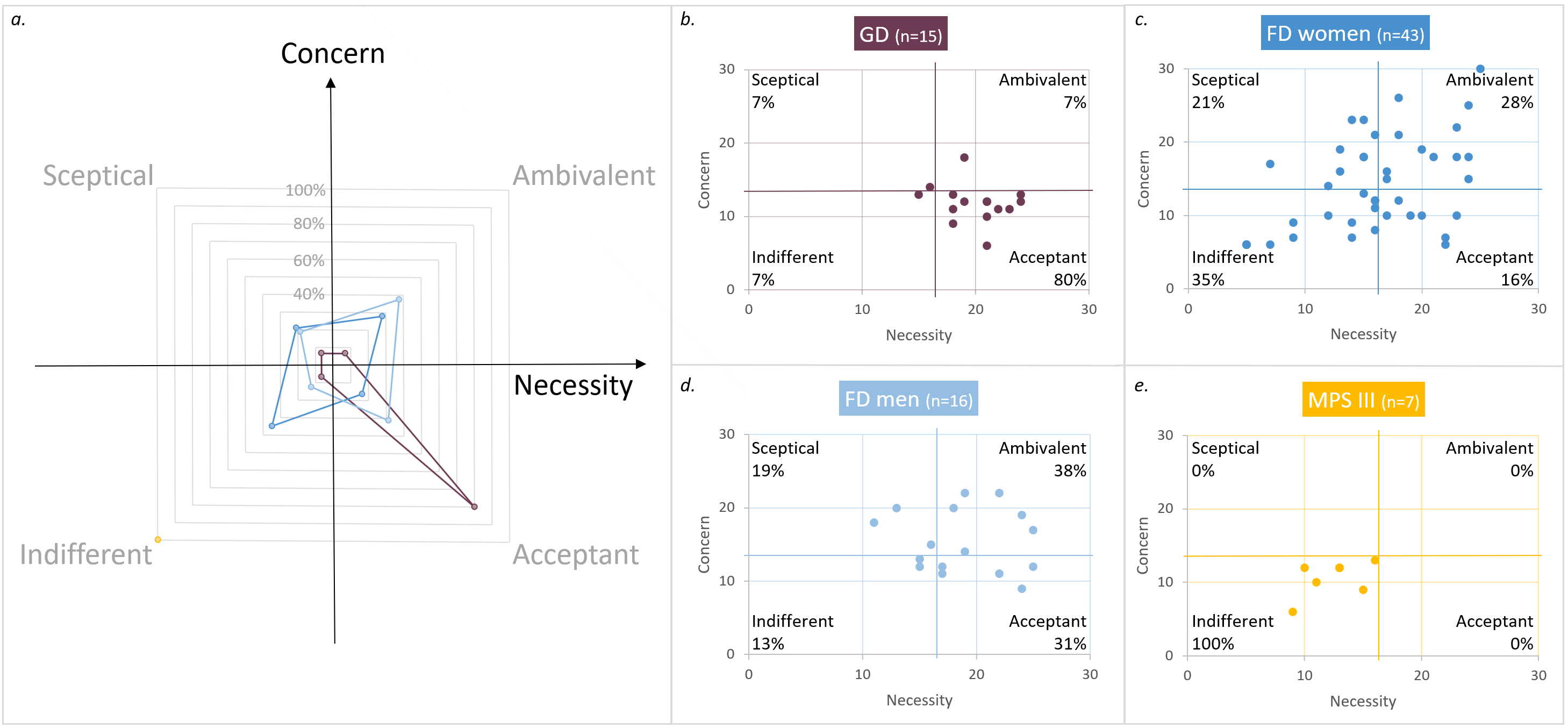


**Supplemental Fig 2 – BMQ-S scores per disease group.** Percentage of respondents in each category of the BMQ-S.

Abbreviations: *BMQ-S* Beliefs in Medicine Questionnaire specific-subscale, *FD* Fabry disease, *GD* Gaucher disease, *MPS III* Mucopolysaccharidosis type III A/B
