# Supplementary material for: Different diseases, different needs: Patient preferences for gene therapy in lysosomal storage disorders, a probabilistic threshold technique survey
Source: Orphanet J Rare Dis. 2024 Oct 3;19:367. doi: 10.1186/s13023-024-03371-y (PMC11451020; doi:10.1186/s13023-024-03371-y)
Supplement: Supplementary file 4 — Additional file 4. [file 13023_2024_3371_MOESM4_ESM.docx]

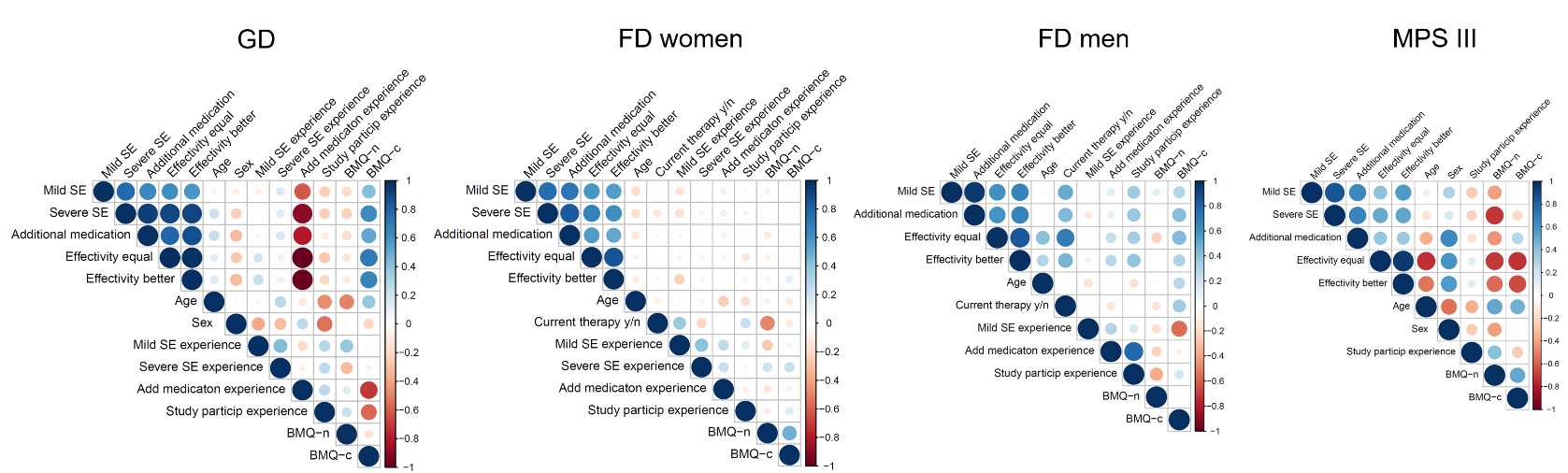


**Supplemental Fig 3 – Correlation matrices of variables per disease group.** Variables that were identical in all respondents of a group were removed from this analysis. The variable sex was coded as 1 (low) for “woman” and 2 (high) for “man”. The variables regarding experience with attributes were coded as 1 (low) for “yes” and 2 (high) for “no”.

Abbreviations: *BMQ-c* Beliefs in Medicine questionnaire concern score, *BMQ-n* Beliefs in Medicine questionnaire necessity score, *FD* Fabry disease, *GD* Gaucher disease type 1, *MPS III* Mucoplysaccharidosis type III A/B, *SE* side effects
