# Supplementary material for: Different diseases, different needs: Patient preferences for gene therapy in lysosomal storage disorders, a probabilistic threshold technique survey
Source: Orphanet J Rare Dis. 2024 Oct 3;19:367. doi: 10.1186/s13023-024-03371-y (PMC11451020; doi:10.1186/s13023-024-03371-y)
Supplement: Supplementary file 5 — Additional file 5. [file 13023_2024_3371_MOESM5_ESM.docx]

Supplemental Table 1:

| **Therapeutic options** | |  | **Administration route and frequency** | **Most common side effects** | | **Potential severe side effects** |
| --- | --- | --- | --- | --- | --- | --- |
| *Gaucher disease* | | | | | | |
|  | velaglucerase alfa | ERT | Intravenously, once every two weeks | Rare: headache, dizziness, GI symptoms, fatigue | | Rare: infusion reaction |
|  | imiglucerase alfa | ERT | Intravenously, once every two weeks | Rare: headache, dizziness, GI symptoms, fatigue | | Rare: infusion reaction |
|  | eliglustat | SRT | Orally, two times per day | Common: Headache, dizziness, GI symptoms, fatigue | | Common: Allergic reaction  Frequently: Syncope |
| *Fabry disease* | |  | | |  | |
|  | agalsidase alfa | ERT | Intravenously, once every two weeks | Common: infusion reaction (specifically in classically affected males), dizziness, syncope, GI symptoms, fatigue | | Common: Infusion reaction (specifically in classically affected males) |
|  | agalsidase beta | ERT | Orally, once every two weeks | Frequent: infusion reaction (specifically in classically affected males), dizziness, syncope, GI symptoms, fatigue | | Frequent: Infusion reaction (specifically in classically affected males) |
|  | lucerastat | SRT | Orally, two times per day | Common: flatulence, hot flushes, urinary tract infection, dizziness | | NA |
|  | migalastat | CT | Orally, every other day | Common: headache, GI symptoms, fatigue, nasopharyngitis | | NA |

**Supplemental Table 1:** An overview of the current therapeutic options for Gaucher and Fabry disease. The initial administration frequency is presented, some patients require different frequencies due to individual therapeutic needs. Common is defined as a frequency of 10% or more; frequent as 1-10% and rare as up to 1%. Reported side effects are based on literature as reported in supplemental table 2. In addition www.farmacotherapeutischkompas.nl was accessed at June 27^th^ 2024. Abbreviations: *CT* chaperone therapy, *ERT* enzyme replacement therapy, *GI symptoms* gastrointestinal symptoms (including diarrhoea, constipation, abdominal pain), *NA* not applicable, *SRT* substrate reduction therapy.
