# Supplementary material for: Different diseases, different needs: Patient preferences for gene therapy in lysosomal storage disorders, a probabilistic threshold technique survey
Source: Orphanet J Rare Dis. 2024 Oct 3;19:367. doi: 10.1186/s13023-024-03371-y (PMC11451020; doi:10.1186/s13023-024-03371-y)
Supplement: Supplementary file 6 — Additional file 6. [file 13023_2024_3371_MOESM6_ESM.docx]

|  | **Current situation** | | | **Gene therapy** | | |
| --- | --- | --- | --- | --- | --- | --- |
|  | **GD** | **FD** | **MPS III** | **GD** | **FD** | **MPS III** |
| References for tasks 1-3 | 61-69 | 70-77 | Not applicable | 18, 34, 78-86 | | 18, 34, 78-87 |
| References for tasks 4&5 | Not applicable | | | 88,89 | *18,* 32, 33, 36-38 | 35, 39-45 |

**Supplemental Table 2: References used to determine base case levels** **depicted in Fig 2.**
